# Optimizing nanopore adaptive sampling for pneumococcal serotype surveillance in complex samples using the graph-based GNASTy algorithm

**DOI:** 10.1101/gr.279435.124

**Published:** 2025-04

**Authors:** Samuel T. Horsfield, Basil C.T. Fok, Yuhan Fu, Paul Turner, John A. Lees, Nicholas J. Croucher

**Affiliations:** 1 MRC Centre for Global Infectious Disease Analysis, Department of Infectious Disease Epidemiology, Imperial College London, London W12 0BZ, United Kingdom;; 2 European Molecular Biology Laboratory, European Bioinformatics Institute, Wellcome Genome Campus, Hinxton CB10 1SA, United Kingdom;; 3 Centre for Tropical Medicine and Global Health, University of Oxford, Oxford OX3 7LG, United Kingdom

## Abstract

Serotype surveillance of *Streptococcus pneumoniae* (the pneumococcus) is critical for understanding the effectiveness of current vaccination strategies. However, existing methods for serotyping are limited in their ability to identify co-carriage of multiple pneumococci and detect novel serotypes. To develop a scalable and portable serotyping method that overcomes these challenges, we employed nanopore adaptive sampling (NAS), an on-sequencer enrichment method that selects for target DNA in real-time, for direct detection of *S. pneumoniae* in complex samples. Whereas NAS targeting the whole *S. pneumoniae* genome was ineffective in the presence of nonpathogenic streptococci, the method was both specific and sensitive when targeting the capsular biosynthetic locus (CBL), the operon that determines *S. pneumoniae* serotype. NAS significantly improved coverage and yield of the CBL relative to sequencing without NAS and accurately quantified the relative prevalence of serotypes in samples representing co-carriage. To maximize the sensitivity of NAS to detect novel serotypes, we developed and benchmarked a new pangenome-graph algorithm, named GNASTy. We show that GNASTy outperforms the current NAS implementation, which is based on linear genome alignment, when a sample contains a serotype absent from the database of targeted sequences. The methods developed in this work provide an improved approach for novel serotype discovery and routine *S. pneumoniae* surveillance that is fast, accurate, and feasible in low-resource settings. Although NAS facilitates whole-genome enrichment under ideal circumstances, GNASTy enables targeted enrichment to optimize serotype surveillance in complex samples.

*Streptococcus pneumoniae* (also known as pneumococcus) is a human nasopharyngeal commensal that can cause severe diseases, such as pneumonia, bacteremia, and meningitis, disproportionately affecting young children and the elderly (Weiser et al. 2018). *S. pneumoniae* infections cause a significant global health burden, being associated with more than 800,000 deaths annually (Ikuta et al. 2022), and are the leading cause of death in children under 5 years of age (Wang et al. 2016; Wahl et al. 2018). The species can be divided into >100 serotypes (Ganaie et al. 2020), each of which expresses an immunologically distinct polysaccharide capsule that enables the bacterium to evade the host's immune response (Hyams et al. 2010).

Polysaccharide conjugate vaccines (PCVs) target a subset of *S. pneumoniae* serotypes that cause a substantial proportion of invasive pneumococcal disease (IPD) (Croucher et al. 2018), driving a reduction in the global IPD burden (Wahl et al. 2018). This is achieved through a significant perturbation of the pneumococcal population carried in the nasopharynx. Consequently, vaccine-targeted serotypes have been replaced through the expansion of already common serotypes not included in current formulations, and the emergence of previously rare or unknown serotypes, changing the frequency of antimicrobial resistance (AMR) and incidence of disease in *S. pneumoniae* (Ladhani et al. 2018; Lo et al. 2019; van Tonder et al. 2019). Ongoing serotype surveillance is critical to identify significant increases in nonvaccine serotype prevalence, particularly if a serotype is associated with AMR or high invasiveness (Lo et al. 2022). Such dynamics can be monitored through analysis of nasopharyngeal samples, although the frequent carriage of multiple serotypes within a single individual, known as “co-carriage” or “co-colonization,” makes identification of all circulating serotypes challenging (Huebner et al. 2000). This problem is exacerbated by the recent discovery that minority serotypes are often present at low frequency (<25% of pneumococcal cells within an individual), but are still responsible for a notable proportion of transmission events (Tonkin-Hill et al. 2022). Therefore, scalable high-sensitivity serotype assays that can deconvolute mixed samples, and identify novel serotypes, are necessary to update vaccine formulations and public health strategies in response to pneumococcal epidemiological dynamics (Colijn et al. 2020).

The original methods for serotyping pneumococci assay the ability of an unknown isolate to agglutinate in the presence of different antisera that recognize known serotypes (Turner et al. 2011; Habib et al. 2014). Agglutination assays have high specificity when applied by experts, but have extensive training requirements, as precise typing requires a succession of tests with different antisera (Satzke et al. 2015). When applied to individual colonies, such methods have a low sensitivity for detecting co-carriage, although this can be improved using latex agglutination of plate sweeps (Turner et al. 2011). Nevertheless, these methods cannot identify novel serotypes. These may be discovered through whole-genome sequencing (WGS) approaches, which detect specific sequence variants of the capsular polysaccharide (*cps*) biosynthesis locus (herein called CBL) (Kapatai et al. 2016; Epping et al. 2018; Sheppard et al. 2022), the operon that defines pneumococcal serotype (Bentley et al. 2006). Yet WGS of individual colonies is difficult to deploy at scale in resource-limited settings as it is expensive and time-consuming, requiring specific expertise and access to specialist laboratory equipment (Jauneikaite et al. 2015). The limited number of colonies from a single patient that can be feasibly sequenced limits the ability of WGS to detect co-carriage, unless a sample is subjected to deep sequencing (Tonkin-Hill et al. 2022). However, this reduces the number of samples that can be analyzed, and therefore lowers overall throughput. Additionally, both agglutination assays and WGS rely on prior selective culture of *S. pneumoniae* as means of enrichment to improve sensitivity. Selective culture adds additional time, resource, and expertise requirements to already complex laboratory workflows, limiting throughput, and potentially resulting in false negatives if cells fail to grow (Ricketson et al. 2021). Purely genotypic approaches, such as PCR and DNA microarrays, target CBL DNA sequences directly present in the sample and therefore do not require selective culture. These methods can identify co-carriage, and are less laborious and expensive than agglutination assays or WGS, and can therefore be used in high-throughput settings (Jauneikaite et al. 2015). However, these methods require target CBL sequences to be specified a priori, and so cannot detect novel serotypes. Overall, no current serotyping method can scalably and sensitively identify both known and novel serotypes, as well as co-carriage.

Novel nucleotide sequencing approaches have the potential to allow accurate, simple, and relatively inexpensive culture-free *S. pneumoniae* surveillance. Nanopore sequencing, developed by Oxford Nanopore Technologies (ONT), is a portable long-read nucleotide sequencing technology in which DNA or RNA molecules are sequenced as they move across an impermeable membrane through protein nanopores (Ip et al. 2015; Quick et al. 2016). Reads are generated in real-time, enabling on-flowcell enrichment of sequences of interest, referred to as “target” DNA, via rejection of all other sequences, referred to as “nontarget” DNA. These methods, known collectively as nanopore adaptive sampling or “NAS,” align the first segment of DNA fragments as they pass through a nanopore to a reference database, before sending a signal back to the sequencer to either “accept,” where the fragment is sequenced to completion, or “reject,” where voltage across the nanopore is reversed, ejecting the fragment (Payne et al. 2021). NAS increases target sequence yield by rejecting nontarget DNA, increasing the sensitivity for detecting of sequences of interest (Payne et al. 2021; Weilguny et al. 2023). This makes NAS well-suited for metagenomics, the culture-free DNA sequencing-based analysis of mixed samples (Ye et al. 2019), such as nasopharyngeal communities. NAS has been shown to increase target yield approximately fourfold (Marquet et al. 2022; Su et al. 2023), and by extension enabling multiplexing of samples on ONT devices to increase throughput. Furthermore, increased target yield has been shown to improve the accuracy of downstream analyses such as variant calling and assembly when analyzing metagenomes (Martin et al. 2022; Weilguny et al. 2023; Wrenn and Drown 2023).

NAS is available as part of the standard ONT sequencing software platform. However, there has been limited quantification of its accuracy, particularly in metagenomics. It has been previously shown that NAS sensitivity is highest when a target present in a metagenome is closely related to a sequence in the reference database (Martin et al. 2022; Viehweger et al. 2023). However, high genetic relatedness between nontarget and target taxa in the same sample has the potential to negatively impact NAS specificity, as target and nontarget reads will be more difficult to distinguish between during the rejection process. The sequence similarity between *S. pneumoniae* and other members of the *Streptococcus* genus (Marttinen et al. 2015; D'Aeth et al. 2021), which are also present as part of the upper respiratory tract microbiome (Bek-Thomsen et al. 2008), is comparable with the error rate of individual ONT reads (Delahaye and Nicolas 2021). Hence, attempts to enrich for a whole *S. pneumoniae* genome may be limited by the challenge of resolving pneumococcal DNA from that of nonpathogenic streptococci. Alternatively, targeting loci that are specific to *S. pneumoniae* will improve target enrichment, as such sequences are typically absent from benign commensals. Hence, CBL sequences are a promising candidate for targeted metagenomics enrichment (Bentley et al. 2006; Croucher et al. 2018; Løchen et al. 2022).

Here, we benchmark the sensitivity and specificity of NAS for detection of *S. pneumoniae* in mixed samples, and assess the ability of NAS to quantify serotype prevalence in co-carriage samples using different target databases. To improve performance when detecting novel serotypes, we develop a graph-based bioinformatic method for NAS, named GNASTy (Graph-based Nanopore Adaptive Sampling Typing, pronounced “nasty”), and benchmark it against the current NAS implementation, which uses linear alignment. Overall, we demonstrate the advantages and caveats of NAS for application in metagenome-based *S. pneumoniae* surveillance, and introduce a new method for detection and discovery of novel serotypes in metagenomes.

## Results

### NAS performance depends on microbiome composition

We first set out to determine the taxonomic range across which NAS can effectively enrich for a target sequence, while still correctly rejecting nontarget sequences. We hypothesized that NAS would fail to enrich for target loci when the sequence similarity between target and nontarget genomes was comparable to the single-strand error rate of ONT reads (∼6% [Delahaye and Nicolas 2021]), resulting in incorrect selection of nontarget DNA that ultimately reduces target enrichment.

**Figure 1.**
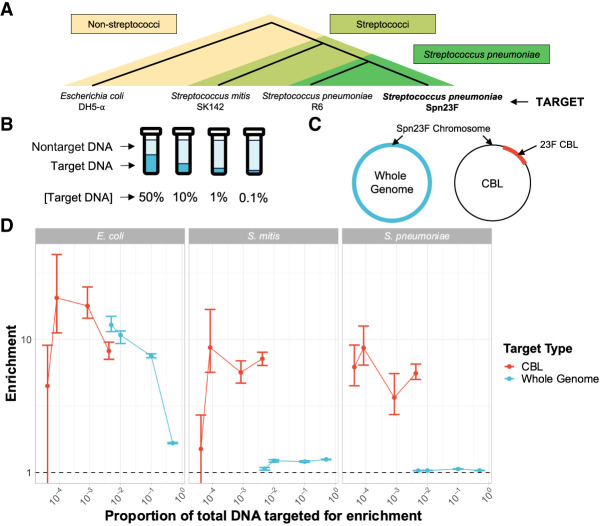
Enrichment of *S. pneumoniae* Spn23F in samples containing closely and distantly related nontarget species. (*A*) Representation of evolutionary relatedness of nontarget species and the target, *S. pneumoniae* Spn23F. (*B*) Experimental setup of target DNA dilution series with nontarget DNA. (*C*) Representation of two enrichment experiments, either targeting the whole Spn23F genome (blue) or the 23F CBL sequence present on the Spn23F Chromosome. (*D*) Enrichment results of Spn23F whole genome or 23F CBL at different concentrations of target DNA. Bar ranges are an interquartile range of enrichment from 100 bootstrap samples of reads. Data points connected by lines are observed enrichment values for each library, with solid lines connecting target DNA diluted at different concentrations with nontarget DNA. Columns describe the nontarget species within each mixture. To plot on a log scale, all enrichment values had 0.01 added to them. Horizontal dashed line describes enrichment = 1 (i.e., no enrichment has occurred).

To test this hypothesis, we generated mock communities containing mixtures of genomic DNA from *S. pneumoniae*, the serotype 23F pneumococcal isolate ATCC 700669 (Croucher et al. 2009), referred to as “Spn23F,” with that of closely and distantly related nontarget species. Spn23F DNA was mixed with DNA from species from a different phylum, represented by *Escherichia coli* DH5-α; the same genus but different species, represented by *Streptococcus mitis* SK142; and the same species but different strain, represented by *S. pneumoniae* R6 (Fig. 1A). To test NAS sensitivity at low target DNA concentrations, Spn23F DNA was titrated from a proportion of 0.5–0.001 (50% − 0.1%) (Fig. 1B) in nontarget DNA. These proportions describe the ratio of total DNA bases within a sample that belong to target DNA. The choices of alignment parameters used for NAS performance comparisons are detailed in the Supplemental Material (Section C.1; Supplemental Table 3; Supplemental Figs. 15, 16). All libraries were size-selected to remove DNA fragments <10 kb in length, as this was shown to improve enrichment (Supplemental Material; Section C.2; Supplemental Tables 4–6; Supplemental Figs. 17–21). NAS was carried out using Readfish (Payne et al. 2021), targeting either the whole Spn23F genome or 23F CBL (Fig. 1C). All samples were multiplexed into a combined sequencing library and run on a single flow cell to control for batch effects. Half of the “channels” (a group of four pores, of which only one is sequencing at one time) sequenced the library using NAS, whereas the other half sequenced the same library normally without NAS (termed “control”). Splitting the flow cell in this way provides an internal control, which is used for the calculation of enrichment by composition (referred to further as “enrichment,” see Methods) (Martin et al. 2022). Using enrichment allows direct comparison of NAS performance across sequencing runs, which may otherwise be confounded by between-run variability. Enrichment >1 indicates that a target was successfully enriched, with a greater proportion of target bases generated using NAS relative to the control.

Comparison of NAS performance based on enrichment is shown in Figure 1D (blue). Spn23F whole-genome enrichment was highest in mixtures containing *E. coli* for all target proportions. Conversely, mixture with *S. mitis* and *S. pneumoniae* resulted in notably lower enrichment, although enrichment was slightly higher in mixtures with *S. mitis*. For example, for the 0.1 target dilution, enrichment of the Spn23F genome was 7.51, 1.20, and 1.05 for the *E. coli*, *S. mitis*, and *S. pneumoniae* mixtures, respectively. Additionally, enrichment increased monotonically as Spn23F DNA proportions decreased in *E. coli* mixtures, as observed previously (Martin et al. 2022), whereas for mixtures with *S. mitis* and *S. pneumoniae* enrichment remained relatively constant between dilutions. These results indicate that the NAS alignment process is not able to effectively reject sequences from nontarget species when their divergence is similar to the read error rate. This result has particular significance for the use of NAS in *S. pneumoniae* surveillance, as the presence of commonly co-occurring streptococci in the nasopharynx greatly impacts NAS performance.

**Figure 2.**
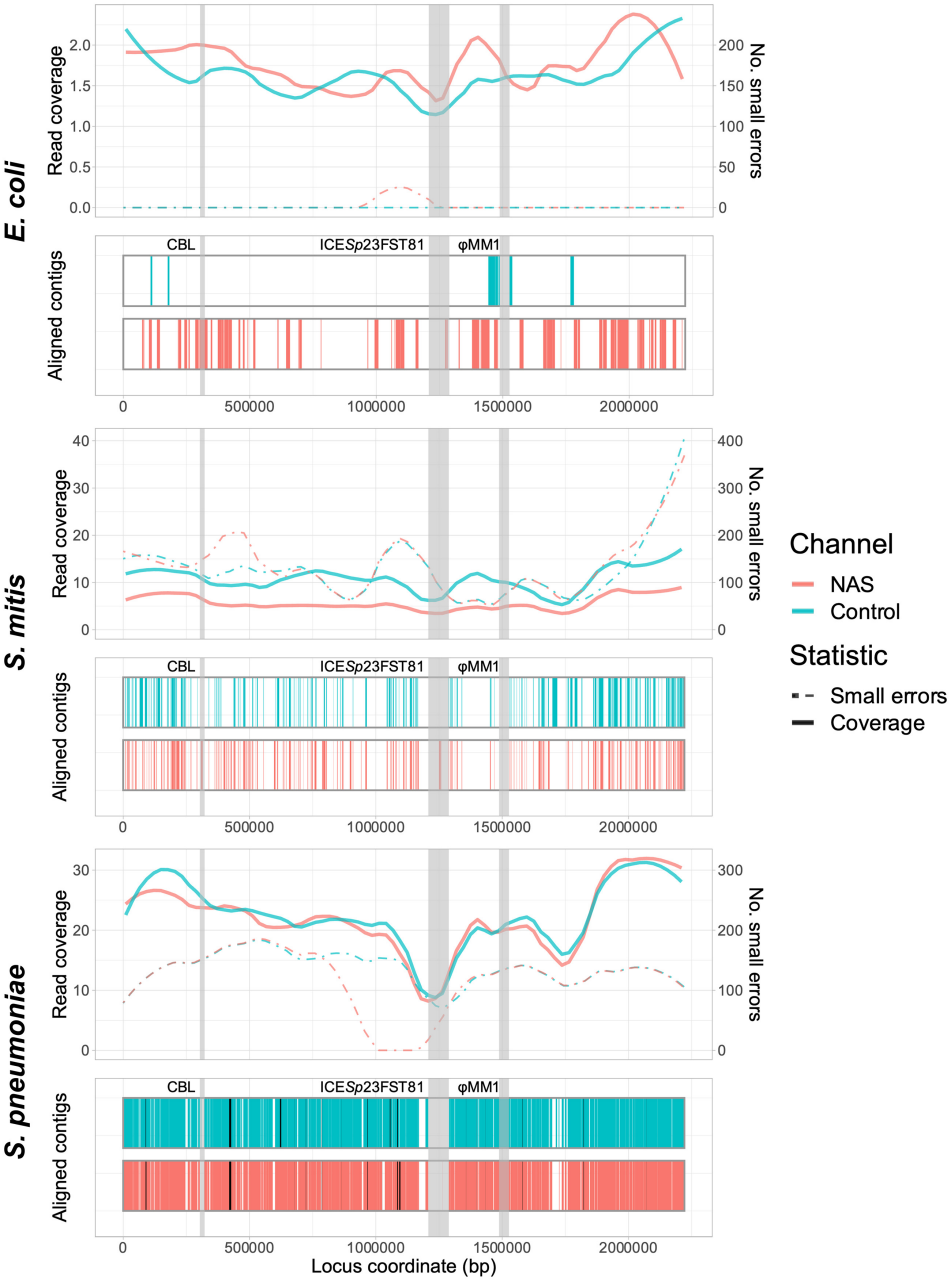
Spn23F whole-genome enrichment assembly comparison. Each panel describes an Spn23F assembly generated from 0.1 Spn23F dilutions with each nontarget organism. For each panel, the *top* plot shows the read coverage (solid), defined as the absolute number of bases aligning to a locus, and number of small errors (≤50 bp, dashed), and the *bottom* plot shows aligned contigs (colors) and large errors (black bars, >50 bp) in each assembly. Loci of interest are annotated by gray bars; CBL, as well as ICE*Sp*23FST81 and ψMM1 prophage, which are missing in this isolate of Spn23F (Croucher et al. 2012).

To determine the effect of nonspecific enrichment on downstream analyses, we then assembled reads using metaFlye (Kolmogorov et al. 2020) and analyzed assembly quality using Inspector (Chen et al. 2021), overlaying results on the Spn23F reference genome for the 0.1 target DNA dilutions. We compared the relative read coverage and aligned contig coverage of the reference genome, as well as the presence of small (<50 bp) and large (≥50 bp) assembly errors (Fig. 2). Greater coverage by aligned contigs indicates that read coverage, and therefore target yield, was sufficiently high to generate a contiguous assembly, whereas the presence of small or large errors suggests problems with the assembly process, such as insufficient read coverage or integration of nontarget reads into assemblies.

For the *E. coli* mixture, the Spn23F whole-genome assembly contained very few errors, although read coverage was low and the assembly covered only a small portion of the Spn23F genome (Fig. 2, top). The resulting assembly from NAS channels had a greater coverage of aligned contigs than that from control channels, coupled with higher read coverage across the Spn23F genome. The assemblies from the dilution with *S. mitis* had greater overall genome coverage than the equivalent *E. coli* mixture, although the respective aligned contigs were short and contained larger numbers of small errors (Fig. 2, middle). Based on read coverage, which was higher in the *S. mitis* mixture over *E. coli* despite Spn23F being at equivalent concentrations, these errors are likely due to the incorporation of nontarget *S. mitis* reads into Spn23F assemblies, ultimately resulting in mismatches with the reference sequence. The dilution of Spn23F with *S. pneumoniae* R6 produced assemblies with the greatest coverage of aligned contigs, although the assemblies also had large numbers of both small and large errors (Fig. 2, bottom). There was also a gap in assemblies at the 23F CBL; *S. pneumoniae* R6 is unencapsulated and so does not possess a CBL, meaning that these assemblies likely contained a large number of nontarget *S. pneumoniae* R6 reads. Comparing NAS and control assemblies across all mixtures, both read and assembly coverage were similar for the *S. mitis* and *S. pneumoniae* mixtures between control and NAS channels, whereas for *E. coli* the NAS channels outperformed the control channels. These results highlight the inability of NAS to distinguish between closely related target and nontarget sequences, resulting in lowered enrichment and chimeric assemblies.

### NAS can effectively enrich for pneumococcal CBL

To improve enrichment by NAS, we specifically enriched for the pneumococcal CBL, which is generally absent from streptococci other than *S. pneumoniae* (Bentley et al. 2006). We sequenced the same library as described in Figure 1, targeting 106 distinct pneumococcal CBL sequences using NAS (see Methods for details of sequences), measuring the enrichment of the 23F CBL present in the Spn23F genome. Targeting all known CBL sequences using NAS would be the best approach when serotyping a novel isolate in the field, as this practice would provide the highest probability of detecting and enriching for a previously observed serotype. Most CBL are ∼20 kb, with a 2.2 Mb genome, and therefore the enrichment values were scaled by 8 × 10^−3^ to account for the smaller target sequence size.

**Figure 3.**
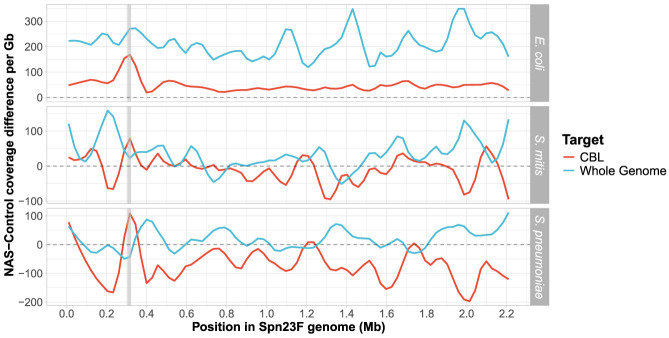
Difference in normalized coverage per locus between NAS and control channels across the Spn23F Chromosome when targeting whole genome (blue) or CBL (red) from 0.1 Spn23F dilutions with each nontarget organism. NAS-control coverage difference per gigabase (Gb) was calculated by normalizing the read coverage for each locus by the amount of data generated (in Gb) for each respective sample and channel, and then negating the normalized coverage for control channels from NAS channels for each locus. The gray dashed line at 0 indicates equivalent coverage at a given locus between NAS and control channels; >0 indicates NAS channels generated greater coverage, <0 indicates control channels generated greater coverage. Data are shown for 0.1 dilutions of Spn23F only. Gray column in each plot highlights the 23F CBL locus. Rows show different species for the nontarget, which was mixed with Spn23F in each sample.

We observed a notable improvement in enrichment when only targeting the 23F CBL, particularly in mixtures containing *S. mitis* and *S. pneumoniae* (Fig. 1D, red). For example, for the 4 × 10^−3^ target dilution, CBL enrichment was 7.16 and 5.46, whereas for the 5 × 10^−3^ target dilution, whole-genome enrichment was 1.06 and 1.02 for *S. mitis* and *S. pneumoniae* mixtures, respectively. For the *E. coli* mixture with Spn23F at 0.1 proportion, the coverage difference between NAS and control channels at the 23F CBL locus was greater when enriching for the whole Spn23F genome than for the 23F CBL, whereas the reverse was true for the *S. mitis* and *S. pneumoniae* mixtures (Fig. 3). These results indicate that when a nontarget species is sufficiently divergent from target species, both whole-genome and CBL enrichment are viable means of serotyping, exemplified by the *E. coli* mixture. However, directly targeting the CBL boosts NAS performance when nontarget species are closely related to the target, exemplified by the *S. mitis* and *S. pneumoniae* mixtures. Therefore, CBL sequences are sufficiently divergent from the rest of the *S. pneumoniae* genome, as well as other closely related genomes, to be differentiated and enriched for. We did not observe the same monotonic increase in CBL enrichment with decreasing target concentration, as observed with whole-genome enrichment. Additionally, bootstrap interquartile ranges were wider for CBL samples compared to whole-genome samples. This is consistent with Martin et al. (2022), in which a predictive model of target enrichment was less accurate at lower target concentrations, indicating that low target concentrations produce more noisy enrichment measures. Overall, CBL enrichment works consistently, independent of the population composition, whereas whole-genome enrichment is dependent on concomitant nontarget species. Furthermore, 23F DNA was still detectable at the lowest concentration tested, meaning that NAS can enrich for target DNA at concentrations as low as 1 in 10,000 bases (targeting 20 kb of 2.2 Mb pneumococcal genome (∼ 1%) in a 0.01 dilution) in a mixed sample. Taken together, these results indicate that targeting CBL for the identification and serotyping of *S. pneumoniae* is a viable alternative to whole-genome enrichment in complex microbial samples.

**Figure 4.**
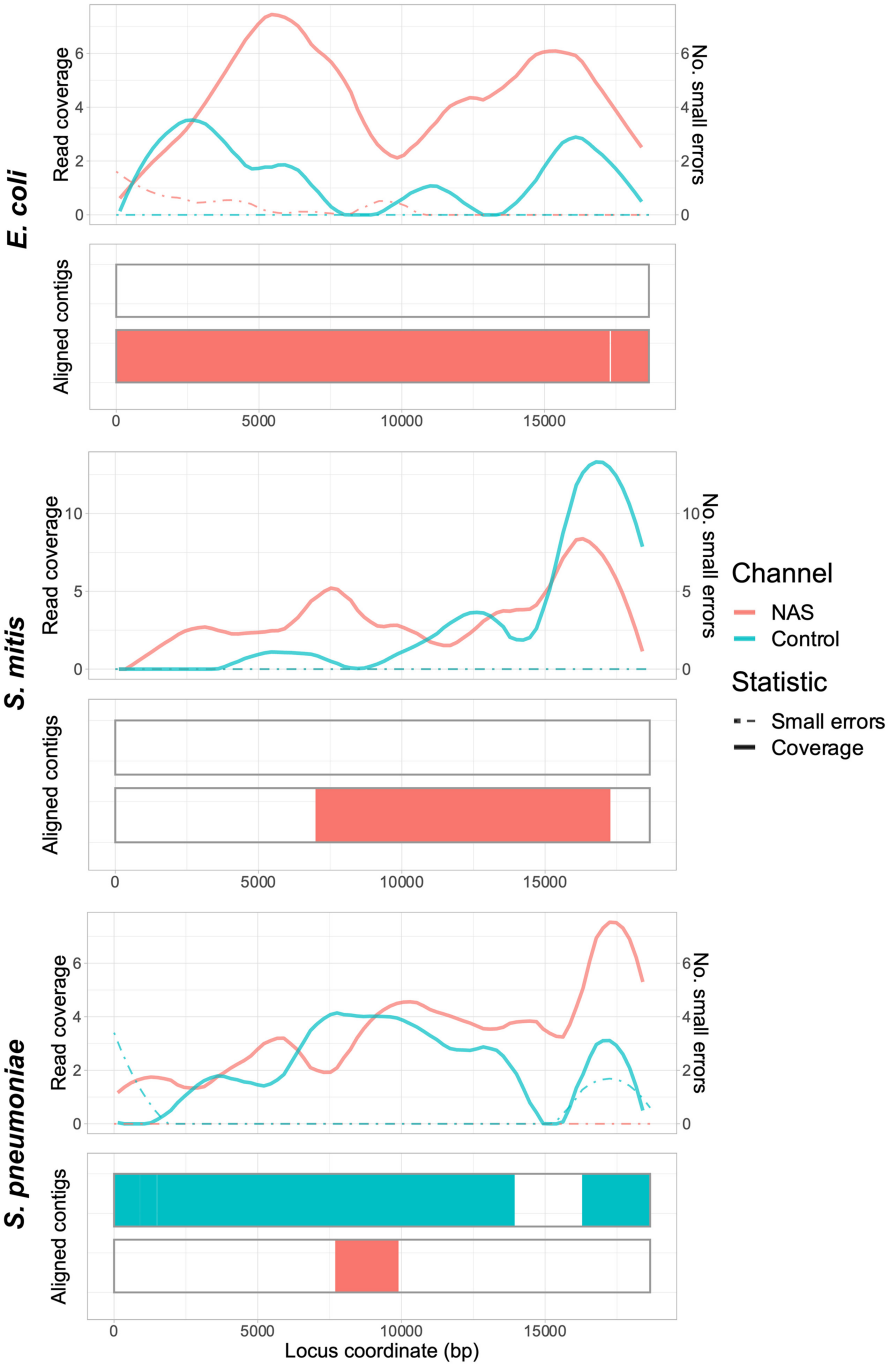
Spn23F CBL enrichment assembly comparison. Each panel describes a 23F CBL assembly generated from 0.1 Spn23F dilutions (8 × 10^−4^ 23F CBL proportion) with each nontarget organism. For each panel, the *top* plot shows the read coverage (solid), defined as the absolute number of bases aligning to a locus, and the number of small errors (≤50 bp, dashed), and the *bottom* plot shows the aligned contigs (colors) and large errors (black bars >50 bp) in each assembly.

We then generated and compared 23F CBL assemblies as before, focusing on 0.1 Spn23F dilutions, equating to 8 × 10^−4^ 23F CBL DNA (Fig. 4). For mixtures containing *E. coli* and *S. mitis*, NAS channels generated more read coverage than control channels, resulting in more complete 23F CBL assemblies containing very few errors. For both whole-genome and CBL assemblies for mixtures containing *E. coli*, we observed slightly higher numbers of small errors for NAS over control channels (Figs. 2, top, 4, top). However, the numbers of small errors for *E. coli* mixtures are relatively low in comparison to the other mixtures, where we additionally observed similar or lower numbers of small errors for NAS compared to control channels, meaning these small errors are likely random noise. For the mixture containing *S. pneumoniae* R6, the 23F CBL assembly was conversely more complete for control channels, likely due to low read counts making the assembly process noisy and leading to patchy coverage for both NAS and control channels (Supplemental Table 2). Overall, NAS improved assembly quality at lower target DNA concentrations over normal sequencing, although low read count made assembly accuracy more variable between samples.

Previous studies have shown that although NAS increases the proportion of target bases within the read data set, it may reduce the absolute yield for an equivalent sequencing time (Payne et al. 2021; Martin et al. 2022). In these instances, normal sequencing would give increased coverage of the target genome, and therefore NAS should be avoided. To determine whether this was the case with enrichment of the whole Spn23F genome and 23F CBL, we compared the total number of bases aligning to target sequences across control and NAS channels (Supplemental Fig. 1). For whole-genome enrichment, the absolute yield was lower for NAS channels on average; however, this difference was not significant. For CBL enrichment, there was a significant increase in absolute yield using NAS (2.17-fold on average, *P* = 0.0049). Furthermore, mean read lengths for aligned reads were 4.2-fold higher (*P* = 7 × 10^−6^) on average in NAS channels than unaligned reads for CBL enrichment, whereas there was no difference for whole-genome enrichment (*P* = 0.37) (Supplemental Fig. 2, results for unselected libraries in Supplemental Fig. 3). Greater difference in read length indicates better performance of NAS, as short unaligned reads and long aligned reads suggest correct rejection of nontarget sequences and acceptance of target reads, respectively (Payne et al. 2021). Therefore, when targeting sequences that are divergent from nontarget DNA, NAS increases both proportional and absolute yield due to better distinction between target and nontarget reads.

### NAS can simultaneously enrich for multiple pneumococcal CBL in the same mixture

**Figure 5.**
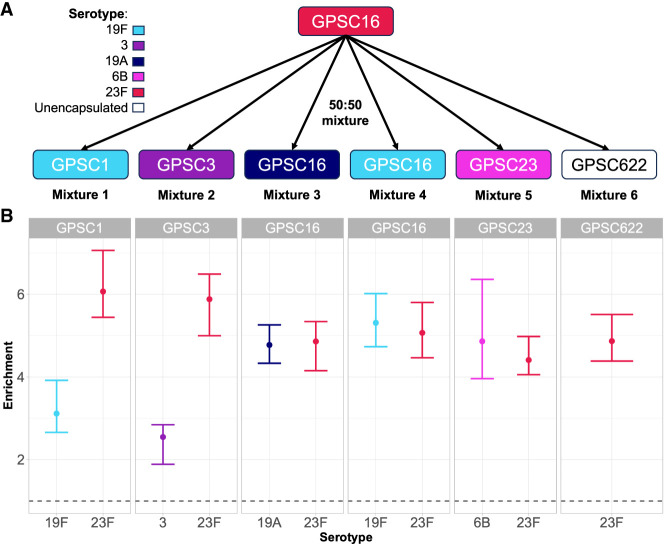
CBL enrichment in mixtures of multiple pneumococci. (*A*) Experimental setup. Spn23F DNA (GPSC16, serotype 23F, red) was mixed in 50:50 proportions with other *S. pneumoniae* isolates with different serotypes (given by color) and genotypes (given by global pneumococcal sequence cluster [GPSC]). (*B*) Enrichment of multiple CBL in mixtures. Bar ranges are interquartile range of enrichment from 100 bootstrap samples of reads. Data points are observed enrichment values for each CBL per library. *x*-axis and color describe the serotype combination of the *S. pneumoniae* isolate mixed with Spn23F; columns describe the GPSC. Dashed line describes enrichment = 1 (i.e., no enrichment has occurred). (GPSC) Global pneumococcal sequence cluster.

NAS is therefore capable of distinguishing encapsulated *S. pneumoniae* from other streptococci, but effective serotype surveillance requires the identification of multiple serotypes in cases of co-carriage. CBL are highly structurally diverse (Bentley et al. 2006), potentially allowing differentiation of multiple CBL in co-carriage by phasing contiguous structural variants using long reads (Cretu Stancu et al. 2017). To determine whether NAS can differentiate and enrich for multiple CBL sequences, we generated a set of mock communities where Spn23F was mixed in 50:50 proportions with other *S. pneumoniae* strains with different genotypes and serotypes (Fig. 5A). We then targeted CBL sequences using NAS; however, we increased the number of times a read can be realigned to the reference sequence before it is rejected (“maxchunks” = 4, rather than 0) to determine whether this would improve enrichment of poorly aligned short reads.

All CBL sequences were enriched across all mixtures, independent of serotype or genotype (Fig. 5B), with NAS significantly increasing the yield of reads aligning to the CBL locus relative to control channels by 1.9-fold on average (*P* = 9.5 × 10^−7^) (Supplemental Fig. 4). Therefore, NAS can be used for targeted sequencing in cases of co-carriage, regardless of respective *S. pneumoniae* serotypes or genotypes. However, CBL enrichment was slightly lower than that observed in mixtures containing a single encapsulated isolate at equivalent concentrations. Comparing the enrichment of the 23F CBL in the 50:50 mixture with the unencapsulated strain, R6, (Fig. 5B, GPSC622), with that observed previously (Fig. 1D, 4 × 10^−3^ target dilution with *S. pneumoniae*), enrichment was reduced (4.9 vs. 5.6). Therefore, increasing the “maxchunks” had a detrimental impact on enrichment and should be kept at zero.

To determine whether NAS improves serotype prediction accuracy in mixed samples, we then analyzed reads from mixed samples using PneumoKITy (Sheppard et al. 2022), a tool for pneumococcal serotype prediction from read data. Serotype predictions were correct for all but mixture 1 using reads from NAS channels, which missed a prediction of 19F (Table 1); however, this serotype was successfully identified in mixture 3. Although we did observe enrichment of the 19F CBL in this mixture (Fig. 5B) the number of bases generated by NAS (53.4 kb) and control (32.4 kb) channels was not sufficient for PneumoKITy to confidently assign 19F as being present in the mixture. For control channels, three mixtures had incorrect serotype predictions, where either one or both serotypes were missed. For samples where two serotypes were correctly predicted, estimated proportions did not deviate substantially from 50%, the expected values for these mixtures, and were similar between NAS and control channels. Therefore, NAS improved the accuracy of co-carriage detection over normal sequencing.

**Table 1.**
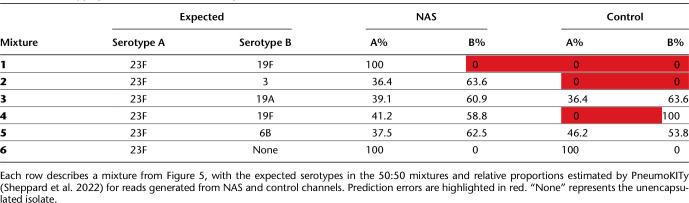
Serotype predictions from mixed samples

### Optimizing serotyping sensitivity from metagenomes with graph-based alignments using GNASTy

We have shown that pneumococcal CBL sequences are a more suitable target for metagenome-based serotype surveillance than whole genomes. However, high sequence divergence between CBL may limit NAS application in the discovery of previously unobserved serotypes. Although SNPs and short variants can usually be aligned to a divergent reference, larger structural variation, present between CBL of different pneumococcal serotypes, can hinder read alignment when variants are not captured in a reference database (Garrison et al. 2018). Such variation can be captured using a pangenome graph, which is a compact representation of multiple linear DNA sequences. Pangenome graphs are constructed by merging similar sequences into nodes, with variation between genomes represented by edges. Pangenome graphs provide a means of recapitulating unobserved structural variation, enabling greater flexibility in alignment to capture novel recombinants (Dilthey et al. 2015) and alignment across assembly gaps (Horsfield et al. 2023). We therefore explored the application of graph alignment in NAS to enrich for novel *S. pneumoniae* CBL.

**Figure 6.**
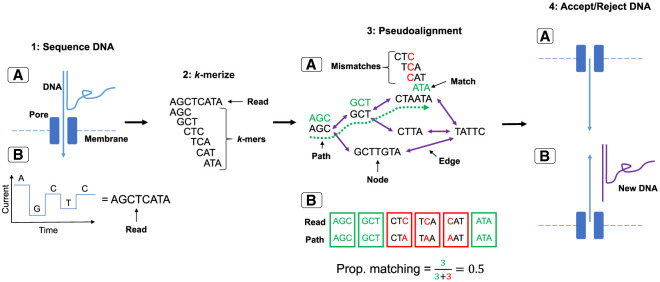
NAS using graph pseudoalignment in GNASTy. (1*A*) The start of a DNA fragment passes through a nanopore, disrupting the movement of ions and causing a change in current determined by the base passing through the pore. (1*B*) This current change is used to basecall the read. (2) The read is *k*-merized, depending on the *k*-mer size used to build the DBG. (3*A*) The *k*-mers are matched to those in the graph via pseudoalignment, analogous to traversing a hypothetical path (dotted green line). (3*B*) The number of matches (green) and mismatches (red) are used to calculate the proportional number of *k*-mer matches between the read and the hypothetical path in the graph. (4*A*) If the read surpasses the predefined identity threshold, the remainder of the DNA is sequenced. (4*B*) If not, the voltage is reversed across the membrane, pushing the read in the reverse direction and freeing the pore to sequence a new DNA fragment.

We developed and implemented a read-to-graph alignment method to replace the linear alignment method currently used in NAS methods (Fig. 6). Our method employs “pseudoalignment,” whereby short overlapping nucleotide sequences, known as “*k*-mers,” are matched between a read and a de Bruijn graph (DBG), a type of pangenome graph built from matching shared *k*-mers between reference sequences (Iqbal et al. 2012; Bray et al. 2016). Pseudoalignment is faster than conventional graph alignment, which uses a seed-and-extend approach between a query and reference sequence, and has been used previously in metagenomic read classification (Mäklin et al. 2021; Alanko et al. 2023). We implemented graph psuedoalignment using Bifrost (Holley and Melsted 2020), which builds colored compacted DBGs, whereby *k*-mers are “colored” by their source genomes, with nonbranching paths of *k*-mers “compacted” into sequences known as “unitigs,” reducing graph size. We named this method “GNASTy.” A detailed description of the GNASTy method is available in the Supplemental Material (Section C.3; Supplemental Fig. 22).

To benchmark the accuracy of GNASTy against the current linear alignment used during NAS, we generated a simulated data set of nanopore reads from the Spn23F and *E. coli* DH5-α reference genomes. Reads originating from the 23F CBL were classified as target reads, and all others were classified as nontarget. We compared the classification accuracy of graph pseudoalignment, implemented in GNASTy, against minimap2 (Li 2018), the linear aligner used in Readfish (Payne et al. 2021). The alignment index for both methods was generated from the 106 CBL sequences used previously. Graph pseudoalignment using GNASTy was carried out with a *k*-mer size of 19 bp based on simulation sequencing run performance (Supplemental Material; Sections C.4, C.5; Supplemental Figs. 23, 34), with a percentage identity, defined by minimum read-graph identity or “S*,*” of 75% between a read and a path within the DBG for a read to be classified as a target. Additionally, minimum read length was set to 50 bp, with unaligned reads below this length being rejected.

We found that alignment sensitivity was higher for graph pseudoalignment than minimap2, whereas specificity was similar between the two methods (Table 2). Therefore, minimap2 had a greater tendency to incorrectly reject target reads than graph pseudoalignment, whereas correct rejection of nontarget reads by graph pseudoalignment was only slightly lower than minimap2. Higher graph pseudoalignment sensitivity is likely due to two factors; graph pseudoalignment does not rely on mapping contiguous blocks of sequence to identify read matches unlike minimap2, allowing more sensitive alignment of reads with structural variants introduced by the read simulator (Yang et al. 2017; Břinda et al. 2018). Furthermore, graph pseudoalignment enables the alteration of alignment identity parameters, which is not possible in the implementation of minimap2 in ReadFish, where these are locked to default values. Both of these factors will likely contribute to the increased sensitivity of graph pseudoalignment over minimap2.

**Table 2.** Alignment accuracy comparison between graph pseudoalignment in GNASTy (referred to as “graph”) and minimap2

Tool	No. TP	No. FN	No. TN	No. FP	Sensitivity	Specificity
Graph	1149	495	494790	3566	0.699	0.993
minimap2	713	931	497514	842	0.434	0.998

Sensitivity is defined as *TP*/(*TP* + *FN*), specificity is defined as *TN*/(*TN* + *FP*).

When comparing computation speed between the two methods, minimap2 outperformed Bifrost/GNASTy during index generation and read alignment. Minimap2 was 30-fold faster at index generation than Bifrost and used 4.5-fold less memory, although Bifrost generated an index twofold smaller than minimap2 (Supplemental Table 1). This is an upfront cost not relevant during sequencing. Per-read alignment times for graph pseudoalignment were notably higher than those for minimap2 (Supplemental Fig. 5). For graph pseudoalignment, all reads were individually aligned in <1/8th of a millisecond, equivalent to sequencing 0.056 bases assuming a rate of 450 bases sequenced per second (Payne et al. 2021). If 512 reads were aligned in a single chunk (the maximum number of reads that could be generated at once on a MinION), this would be equivalent to an additional 29 bases being sequenced per pore before a decision is made to accept or reject each read. Therefore, we tested whether GNASTy's greater sensitivity for detecting target reads, at the cost of slower rejection of nontarget reads, would increase the enrichment of CBL sequences.

### Graph-based alignment facilitates the discovery of novel CBL

We investigated whether GNASTy's graph representation of CBL variation would enable it to discover and enrich novel CBL variants more accurately than conventional NAS. To evaluate this, we tested whether graph pseudoalignment in GNASTy could outperform linear alignment when the target sequence was not present in a reference database. We used the 106 CBL sequences used previously as a reference database, removing the 23F CBL, along with all closely related CBL (cluster two from Mavroidi et al. [2007]). We then sequenced the same samples used previously (Fig. 1A,B), this time using V14 rather than V12 Nanopore chemistry, and calculated the enrichment of the 23F CBL. These experiments used V14 sequencing chemistry, which generates reads faster than the now-discontinued V12 chemistry, but has similar read quality (Sinclair Dokos 2022). We conducted sequencing runs using three different alignment methods. Minimap2 was compared with graph psuedoalignment in GNASTy with *k* = 19 and minimum read length of 50 bp as before. We tested two percentage identity thresholds for graph pseudoalignment, *S* = 75% and *S* = 90%, to understand the effect of increasing graph pseudoalignment stringency on enrichment.

**Figure 7.**
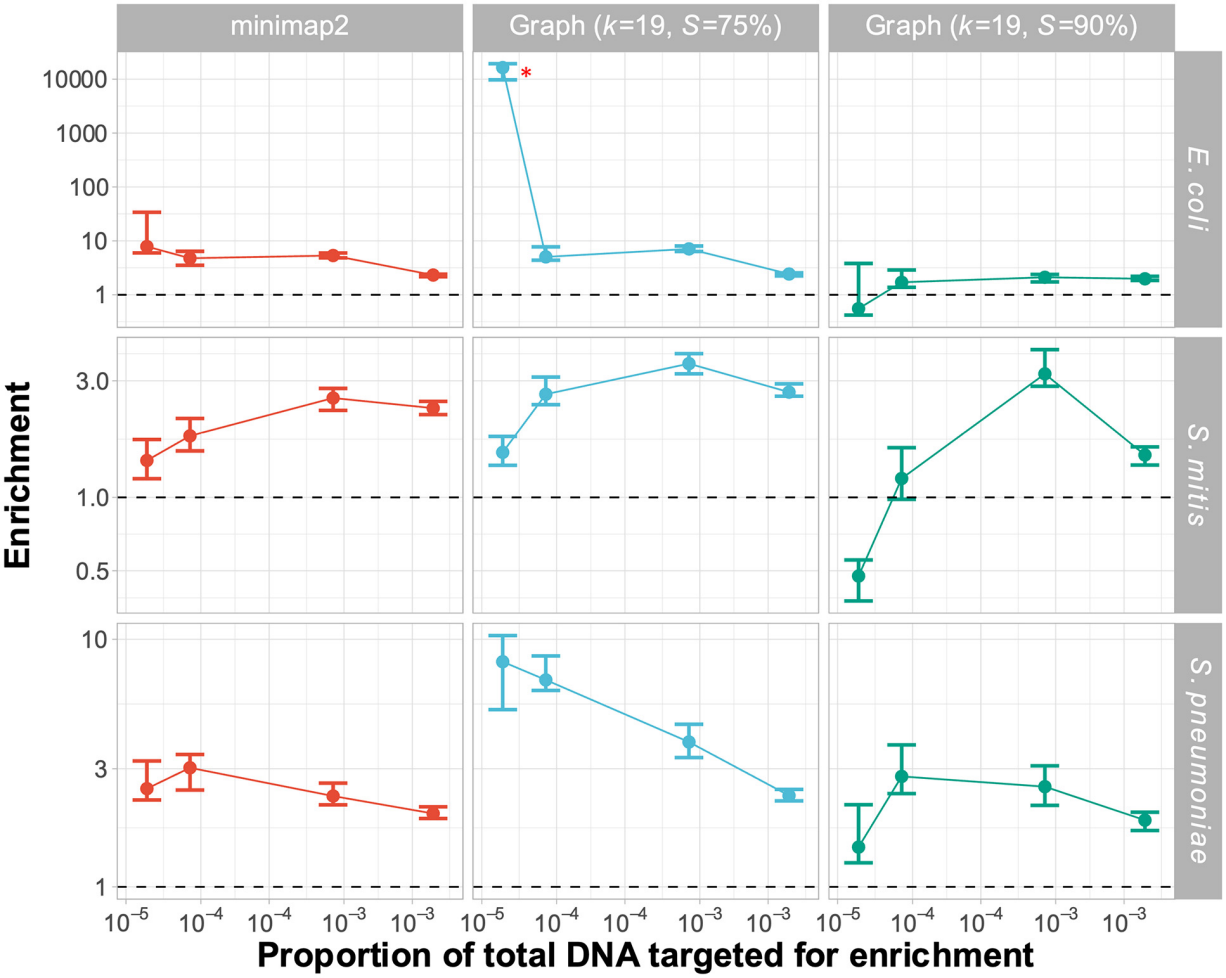
Enrichment comparison of 23F CBL at different concentrations of target between minimap2 and graph pseudoalignment in GNASTy when aligning to a partial CBL reference database. Bar ranges are interquartile range of enrichment from 100 bootstrap samples of reads. Data points connected by lines are observed enrichment values for each library, with solid lines connecting the same genome diluted at different concentrations. Rows describe the nontarget species in the mixture; columns describe the alignment method used. Each column represents data from a single flow cell. To plot on a log scale, all enrichment values had 0.01 added to them. Horizontal dashed line describes enrichment = 1 (i.e., no enrichment has occurred). Red asterisk marks high enrichment observed using graph pseudoalignment (*k* = 19, *S* = 75%) in the *E. coli* mixture at 4 × 10^−5^ target proportion.

Results showed that enrichment could be achieved by all NAS methods and parameter values, although graph pseudoalignment (*S* = 75%) performed best, with equivalent or higher enrichment than minimap2 across all samples (Fig. 7). The highest observation of 23F CBL enrichment exceeded 10,000 for graph pseudoalignment (*S* = 75%) in the *E. coli* mixture (identified by a red asterisk), which was due to no 23F CBL control reads being generated for this sample, while NAS enabled detection of target DNA. As observed in Martin et al. (2022), targets at low concentration produce more variable enrichment values due to the low numbers of reads detected by both NAS and control channels. Overall, the slower read alignment speed of graph pseudoalignment compared to minimap2 did not have a large enough effect to negatively impact enrichment. In addition to enrichment, absolute yield of 23F bases was significantly increased using graph pseudoalignment (*S* = 75%) relative to control channels (Supplemental Fig. 6). Graph pseudoalignment (*S* = 75%) achieved a mean yield increase of 2.75-fold (*P* = 9.8 × 10^−4^), which was greater than for minimap2, which achieved a mean yield increase of 2.0-fold (*P* = 2.4 × 10^−3^). Furthermore, graph pseudoalignment (*S* = 75%) performed similarly to minimap2 when the 23F CBL was included in the reference database, meaning that graph pseudoalignment in GNASTy can be used as a direct replacement for minimap2 for NAS (Supplemental Material; Section C.6; Supplemental Figs. 35–38). Graph pseudoalignment (*S* = 90%) performed worst of the three methods, resulting in lower enrichment and reduced absolute yield, which was not significantly different from control channels. Enrichment fell below 1 at the lowest target concentrations in *E. coli* and *S. mitis* mixtures, indicating target depletion. This result highlights that *S* = 90% is too stringent for graph pseudoalignment, resulting in incorrect rejection of target reads.

The increased enrichment we observed for graph pseudoalignment in GNASTy over minimap2 may be due to biased over-sequencing of a specific position in the 23F CBL, rather than even coverage of the entire 23F CBL. To enable accurate assembly of the full 23F CBL, coverage should ideally be increased evenly across the target sequence, rather than over-represented in specific regions. Comparison of normalized coverage across the full 23F CBL for minimap2 and graph pseudoalignment showed similar read coverage variability across the 23F CBL all three methods (Supplemental Fig. 7). For example, at the highest target proportion (4 × 10^−3^), there were coverage spikes at both ends of the CBL locus for all three methods. Therefore, enrichment achieved by linear alignment can be explained by mapping of the start of read to shared regions at the ends of CBL (Supplemental Material; Section C.5; Supplemental Fig. 32). Coverage for both minimap2 and graph pseudoalignment fell in the center of the CBL, which is particularly notable at 4 × 10^−3^ target dilutions. At the lowest target dilutions (4 × 10^−5^), a spike in coverage can be observed at the 18 kb position in the 23F CBL for mixtures containing *S. mitis* and *S. pneumoniae*. As the nontarget isolates *S. mitis* SK142 and *S. pneumoniae* R6 both contain *aliA* homologs (Hoskins et al. 2001; Sørensen et al. 2016), which is also found at the end boundary of all *S. pneumoniae* CBL (Bentley et al. 2006), this peak can be attributed to nonspecific enrichment of a gene common to streptococci. However, coverage was equivalent or higher for graph pseudoalignment (*S* = 75%) over minimap2 across all target concentrations and nontarget species. In summary, although NAS can enrich for novel serotypes using linear alignment, using GNASTy increases NAS sensitivity.

Next, we compared the ability for minimap2 and GNASTy to correctly identify the 23F serotype in the mixtures using PneumoKITy (Supplemental Fig. 8). We compared the proportion of the 23F CBL reference sequence covered by the reads, which is used by PneumoKITy as a proxy for serotype prediction confidence (Sheppard et al. 2022). Minimap2 and graph pseudoalignment (*S* = 75%) performed similarly, with reads from NAS channels providing more support for the 23F CBL call than for controls in all cases. Even at low target concentrations (≤8 × 10^−5^ target proportion), these alignment methods were still able to identify 23F as the most likely serotype, with the exception of the mixture with *S. pneumoniae* R6, where serotype 2 was predicted to be the most likely serotype. *S. pneumoniae* R6 is derived from a serotype 2 strain via deletion of its respective CBL (Iannelli et al. 1999); however, the presence of CBL flanking sequences in *S. pneumoniae* R6, as described above, likely lead to false detection of serotype 2 CBL.

We then compared assemblies of the 23F CBL across the three alignment methods. We chose samples containing 0.1 Spn23F dilution with *S. mitis* to mimic carriage of a single isolate (Supplemental Fig. 9). For all alignment methods, read coverage was higher for NAS channels than for control channels, although graph pseudoalignment (*S* = 90%) had the lowest absolute coverage for both channel types. Despite variation in coverage, all assemblies covered a majority of the CBL with minimal errors of any kind. Assembly completeness was similar between control and NAS channels, except at the right end of the CBL, where minimap2 and graph pseudoalignment (*S* = 90%) were unable to generate an aligning contig. This effect was also observed when using a full CBL database for enrichment (Supplemental Material; Section C.6), and may be due to uneven local read coverage affecting assembly contiguity, as metaFlye expects uniform coverage for individual strain genomes (Kolmogorov et al. 2020). Additionally, two central regions (∼7.5 kb and ∼12 kb), and a small region in the 18 kb end of the CBL, were missing in the control assembly for graph pseudoalignment (*S* = 75%). However, these were correctly identified when reads were enriched with graph pseudoalignment. When graph pseudoalignment was run with the suboptimally high alignment specificity parameter (*S* = 90%), the NAS assembly was missing a single region (∼7.5 kb) present in the control assembly. Therefore, although assemblies were largely similar between NAS and control channels, these small differences indicate higher graph pseudoalignment stringency slightly lowered assembly quality compared to the control, whereas greater sensitivity for CBL reads improved assembly quality.

### Graph-based alignment enriches CBL in complex samples mimicking the nasopharynx microbiome

Previous experiments demonstrated graph pseudoalignment in GNASTy was capable of enriching for CBL from simple mixtures. Therefore, we tested whether the method was also effective with more realistic microbial compositions that would be observed in the nasopharynx or oral cavity. We used samples containing a mixed culture generated from nasopharyngeal swabs, spiking in Spn23F as before. As there was no ground truth for these samples, it was unknown whether *S. pneumoniae* strains were already present prior to spiking. Spn23F DNA was added to give a final proportion of 0.1 of total DNA in each sample, reflecting typically observed *S. pneumoniae* prevalences in the nasopharynx (Salter et al. 2017), resulting in a final 23F CBL DNA proportion of 8 × 10^−4^. Libraries were run without size selection, as we observed a detrimental effect on extracted DNA yield with mixed culture samples, which did not affect single isolate samples (Supplemental Fig. 10). NAS was conducted using graph pseudoalignment (*k* = 19, *S* = 75%) in GNASTy using a database containing all 106 CBL sequences, including the 23F CBL. As a control, a sample containing Spn23F mixed with *S. pneumoniae* R6 without size selection at 0.1 and 0.5 proportions was also run, and compared with equivalent samples with size selection.

**Figure 8.**
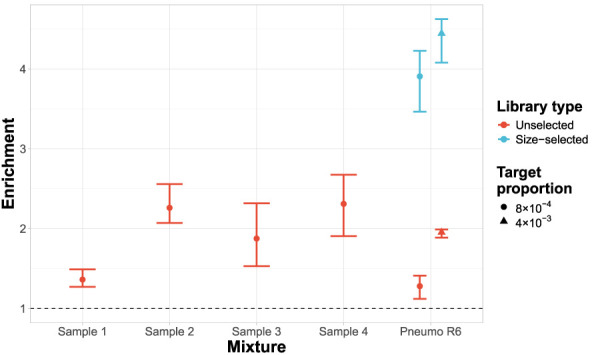
Enrichment of 23F CBL across samples containing mixed cultures from nasopharyngeal swabs. All nasopharyngeal samples (denoted “Sample X”) were run without size selection, with control samples containing Spn23F mixed with *S. pneumoniae* R6 (denoted “Pneumo R6”) without size selection at 0.1 and 0.5 proportions (8 × 10^−4^ and 4 × 10^−3^ 23F CBL DNA proportions, respectively) run alongside. Equivalent control samples from a run with size selection are plotted for comparison. Bar ranges are interquartile range of enrichment from 100 bootstrap samples of reads. Data points are observed enrichment values for each library. The dashed line describes enrichment = 1 (i.e., no enrichment has occurred).

Enrichment of the 23F CBL was achieved for all mixed culture samples (Fig. 8), with all samples performing equivalently to or better than the unselected *S. pneumoniae* R6 sample at the equivalent concentration (8 × 10^−4^). Size selection had a notable positive impact on enrichment in the *S. pneumoniae* R6 mixtures, increasing enrichment from 1.3-1.9 fold to 3.9-4.4 fold for 8 × 10^−4^ and 4 × 10^−3^ target proportions, respectively. This was consistent with the method's performance with the simpler DNA mixtures (Supplemental Material C.2). Therefore, the lowered performance of graph pseudoalignment in GNASTy with these complex samples relative to the simpler mixtures was a consequence of the lack of size selection during DNA sample preparation. This factor also explains the similar target yield between NAS and control channels (Supplemental Fig. 11). Therefore, we advise size selection be used where possible to boost NAS efficiency, although it may not be suitable in all cases due to high DNA yield loss.

The 23F CBL was identified as the most likely CBL in samples 1 and 2, as well as the *S. pneumoniae* R6 mixtures by PneumoKITy, although samples 1, 3, and 4 had evidence of co-carriage (Supplemental Fig. 12), with graph pseudoalignment able to enrich for multiple CBL identified as present by PneumoKITy (Supplemental Fig. 13). In samples 3 and 4, the 23F CBL was not identified as the most likely serotype, although the proportion of the reference 23F CBL sequence matched was above PneumoKITy's confidence cutoff (70%) for reads originating from NAS channels, meaning that these samples were identified as containing a mixture of serotypes. Notably, the prediction for the 23F CBL did not meet the confidence cutoff for reads from control channels in sample 4, meaning that graph pseudoalignment in GNASTy enabled the detection of a low-level secondary serotype that would have otherwise been missed. Assembly quality was similar between NAS and control channels, although NAS channel read coverage was equivalent or higher for most samples (Supplemental Fig. 14). Nevertheless, the read coverage was sufficiently high (>20) to enable assembly in principle, which likely could be achieved by algorithms capable of correcting for the uneven read coverage across the 23F CBL in these data sets. Overall, we have shown that NAS, employing graph pseudoalignment with our tool GNASTy, can be used to enrich for *S. pneumoniae* CBL in mock communities resembling real nasopharyngeal samples.

## Discussion

The complex population dynamics of *S. pneumoniae* reflect the antigenic and genetic diversity of the species, and the serotype replacement that has been driven by the widespread use of serotype-specific vaccines (Ladhani et al. 2018; Lo et al. 2019; Tonkin-Hill et al. 2022). Current methods for *S. pneumoniae* serotype surveillance are limited either by their requirement for application to individual colonies (e.g., WGS or antiserum agglutination), reducing their sensitivity for detecting co-carriage, or by being restricted to only the serotypes known at the time the assay was designed (e.g., PCR genotyping, microarrays). Deep sequencing has revealed the need for sensitive sequencing-based assays that can detect novel serotypes (van Tonder et al. 2019; Tonkin-Hill et al. 2022). However, such data sets are resource-intensive to produce, and necessitate substantial pre-existing infrastructure for library generation, sequencing, and data processing. Therefore, simpler, quicker and cheaper surveillance methods are required to provide a comprehensive view of pneumococcal serotype diversity and prevalence to inform public health strategies.

In this work, we explored the application of NAS to pneumococcal serotype surveillance, which has the potential to fulfill all the above criteria. We found that when targeting a whole *S. pneumoniae* genome in the presence of closely related species, enrichment was reduced, as alignment lacks the specificity to distinguish between target and nontarget reads. However, we showed that targeting the *S. pneumoniae* CBL increases both enrichment and yield of NAS due to the strong association of the sequence with *S. pneumoniae*. We showed this enables the detection, enrichment, and serotyping of multiple CBL simultaneously, and can detect a serotype that only comprises 1% of the sample.

Direct detection of pneumococcal CBL using NAS promises to be a simple, scalable surveillance method. NAS does not rely on culture, reducing the time required to generate a result compared to WGS or agglutination assays. Library construction takes a few hours, and NAS required 1 day of sequencing on a portable MinION device, which can be shortened if sufficient read coverage is reached during sequencing. Although NAS is slower than PCR, which takes a few hours, this time is comparable to microarrays (Jauneikaite et al. 2015). Unlike NAS and microarrays, however, PCR of multiple serotypes cannot be conducted in a single reaction (Pai et al. 2006), increasing workflow complexity despite its shorter runtime. Additionally, NAS provides increased resolution over these genotypic methods, enabling the distinction of serotypes separated by few single or multinucleotide polymorphisms (Mauffrey et al. 2017). NAS is also fully portable and simple enough to be run with limited equipment, requiring only bench-top apparatus such as a centrifuge, thermocycler (Quick et al. 2016), and laptop with a suitable GPU, as used here. NAS is more expensive than PCR and microarrays due to the cost of sequencing reagents (Jauneikaite et al. 2015). However, NAS has lower entry costs compared to other sequencing technologies, such as Illumina or PacBio, with higher target yield enabling sample multiplexing to reduce per-sample costs. We observed greater than twofold increases in target yield compared to standard sequencing using NAS, enabling twice the number of samples to be run on the same flow cell to achieve the same target coverage, therefore halving per-sample costs. Lowered costs, coupled with the portability of Nanopore sequencing, make NAS attractive for applications in low-resource settings where pneumoccocal disease burdens are highest (Troeger et al. 2018).

Despite the potential for NAS to be used in serotype surveillance, the extensive structural variation distinguishing different CBL caused us to hypothesize that the standard linear alignment employed by NAS would have limited sensitivity when applied to novel or variant loci. To address this issue, we developed a pangenome graph-based alignment method for NAS, GNASTy. We showed that GNASTy enables greater enrichment of novel CBL over linear alignment, and is therefore capable of discovering rare or previously unknown serotypes. Therefore, GNASTy combines the advantages of NAS described above with the added benefit of increased sensitivity to enrich for novel serotypes. Unlike PCR and microarrays, GNASTy is capable of identifying novel serotypes and easily adding new targets, with any updates to the serotyping panel achieved through simply extending the reference database, without alterations to the laboratory protocol. Therefore, GNASTy is well suited for surveillance of diverse pathogen biomarkers, such as CBL, where novel variants are discovered frequently (Ganaie et al. 2020, 2023), necessitating repeated panel updates which would otherwise be time-consuming and expensive using PCR or microarrays. Overall, GNASTy provides a balance of accuracy, simplicity, and cost-effectiveness, making it well-suited for routine pneumococcal surveillance in both high- and low-resource settings.

A key improvement of targeted sequencing over shotgun metagenomic sequencing is the improved limit of detection, meaning more rare sequences can be identified. We showed that NAS can increase the proportion of target DNA more than 10-fold over that of the control channels, based on the normalized measure; enrichment by composition, when applied to CBL sequences at concentrations < 0.01%, in line with previous evaluations of NAS efficiency (Martin et al. 2022; Weilguny et al. 2023). We also showed that NAS significantly increased absolute yield of target reads, which improved assembly coverage and accuracy, and increased the sensitivity of DNA-based serotyping in samples mimicking co-carriage. Finally, we showed that GNASTy can enrich for CBL DNA in samples mimicking the complexity of the nasopharyngeal microbiome, and improves serotyping accuracy over normal sequencing. We note that these conclusions are based on single sequencing runs, which is common practice when analyzing NAS performance due to the cost of nanopore sequencing (Payne et al. 2021; Martin et al. 2022). Although we used the normalized measure of enrichment to negate effects of variability between experiments, and bootstrapping to account for noise in data generation, this lack of replicate data sets should motivate future additional validation of this method with different sample types. Furthermore, in the mock communities used throughout this work, our measures of *S. pneumoniae* abundance were relative proportions based on measures from observed nasopharyngeal microbiomes (Salter et al. 2017). We did not convert target concentrations into absolute concentration values (e.g., in ng/µL of target DNA), as sequencing sensitivity will be dependent on the number of bases generated per sample, which itself is contingent on multiplexing and variability in DNA loading onto the flowcell.

Multiplexing is key to enabling the batch processing necessary for NAS-based methods to be viable for use in routine surveillance. Based on our experience, we recommend sequencing between 12 and 24 samples on a single flowcell to provide sufficient coverage to detect *S. pneumoniae* DNA, while reducing the cost per sample through multiplexing. Such relatively small batches are practical in routine local surveillance applications where small clusters of samples are available, contrasting with the hundreds of samples that need be multiplexed for higher-throughput sequencing methods to be maximally cost-effective.

The current limitations of NAS and GNASTy primarily represent the challenges of optimizing DNA sample preparation. The mock communities tested did not contain human reads; however, oral and nasopharyngeal samples often contain substantial host DNA, which will ultimately impact target yield. Therefore, GNASTy will require further optimization to include host DNA depletion, for which suitable laboratory methods are available (Charalampous et al. 2019; Nelson et al. 2019). One potential solution is to use NAS to deplete human DNA as off-target reads, which is possible if the human genome were supplied as a database of unwanted sequence. Such depletion sequencing is more effective than targeted sequencing when sampling the full bacterial diversity of a sample (Marquet et al. 2022). Hence, GNASTy may have additional utility when applied to culture-free nasopharyngeal samples using depletion sequencing. Additionally, when targeting pneumococcal CBL sequences, GNASTy will not be able to distinguish similar or identical sequences found in nonpneumococcal species that coinhabit the nasopharynx, such as *S. mitis* or *Streptococcus oralis* (Gertz et al. 2021). This issue can be addressed by identifying species-specific flanking regions present in reads that start within the CBL but end outside of it (D'Aeth et al. 2021). Here, GNASTy generated up to 41 kb of flanking sequence for reads aligning to target CBL, which can be used to assign a detected CBL to a given species.

NAS has the potential to enable accurate, direct, and relatively inexpensive *S. pneumoniae* surveillance. However, this work highlights the current limitations of enriching for low-abundance species with NAS in mixtures containing closely related taxa, and the suboptimal sensitivity for identifying loci that are not present in the target database. We have developed and tested NAS for the detection and serotyping of *S. pneumoniae* in complex samples, providing methodological recommendations and a novel pangenome graph-based method, GNASTy, for use by public health researchers, which we hope will improve access to accurate *S. pneumoniae* surveillance in low-resource settings. GNASTy promises to be a powerful method both for routine epidemiology, and novel serotype discovery.

## Methods

### Isolate and sample acquisition

All isolate bacterial strains used in this work included: *E. coli* DH5-α, *Moraxella catarrhalis* 0193-3, *Haemophilus influenzae* 0456-2, *S. mitis* SK142, *S. oralis* SK23, *S. pneumoniae* ATCC 706669 (referred to as “Spn23F,” GPSC16, serotype 23F), *S. pneumoniae* R6 (GPSC622, unencapsulated), *S. pneumoniae* 110.58 (GPSC81, unencapsulated), *S. pneumoniae* MalM6 (GPSC16, serotype 19F), *S. pneumoniae* 8140 (GPSC16, serotype 19A), *S. pneumoniae* Tw01-0057 (GPSC1, serotype 19F), *S. pneumoniae* K13-0810 (GPSC23, serotype 6B), and *S. pneumoniae* 99-4038 (GPSC3, serotype 3).

Nasopharyngeal swab samples were chosen from a collection originating from a study of mother–infant pairs in the Maela camp for refugees in Thailand (Turner et al. 2012, 2013). This research complied with all relevant ethical regulations, and was approved by the Ethics Committee of The Faculty of Tropical Medicine, Mahidol University, Thailand (MUTM-2009-306), and by the Oxford Tropical Research Ethics Committee, Oxford University (OXTREC-031-06). All women gave written informed consent to participate in the study. Individuals did not receive monetary compensation for their participation.

### Bacterial culture and DNA extraction

For culture, glycerol stocks containing bacterial isolates and nasopharyngeal swab (referred to as “mixed culture”) samples were inoculated in 10 mL of Todd-Hewitt broth (Oxoid) and 2% yeast extract (Sigma-Aldrich) and cultured overnight at 35°C in 5% CO_2_ atmosphere. For the culture of *M. catarrhalis* and *H. influenzae*, 3 mM hemin (X factor) and 22.5 mM nicotinamide-adenine-dinucleotide (V factor) were also added to respective inocula. Liquid cultures of *M. catarrhalis*, *H. influenzae*, and *E. coli* and mixed cultures were incubated with shaking at 150 rpm. Following incubation, the inocula were centrifuged at 16,000*g* for 10 min, with supernatant being discarded to obtain cell pellets.

DNA was extracted from cell pellets using the Wizard Genomic DNA Extraction Kit (Promega A1120). For *S. pneumoniae* isolates and mixed culture samples, cell pellets were resuspended in 480 µL 50 mM EDTA, before the addition of 120 µL freshly prepared lysozyme (30 mg/mL). The solution was incubated at 37°C for 60 min, before centrifugation at 16,000*g* for 2 min, with the supernatant being discarded. For all isolates, 600 µL nuclei lysis solution was added to pellets and incubated at 80°C for 5 min. Three microliters RNase solution was then added and incubated at 37°C for 15 min, before cooling to room temperature. Fifty microliters of 20 mg/mL recombinant Proteinase K solution (Invitrogen AM2548) was then added, with the sample being incubated at 55°C for 1 h. Two hundred microliters protein precipitation solution was then added and incubated on ice for 5 min, before solutions were centrifuged at 16,000*g*, and the supernatant transferred to a clean tube. Six hundred microliters of room temperature 100% isopropanol was then added to the supernatant and centrifuged at 16,000*g*, with the supernatant being discarded. Six hundred microliters of room temperature 70% ethanol was then added to the pellet and mixed to resuspend the pellet. The solutions were centrifuged at 16,000*g*, with the supernatant being discarded, and the pellets were allowed to air-dry for 15 min. DNA pellets were then resuspended in 150 µL DNA rehydration solution.

Extracted DNA was size selected using the SRE XS kit (PacBio SKU 102-208-200) following manufacturer's instructions to remove fragments <10 kb in length.

### DNA quality control

Extracted DNA was quantified using a dsDNA broad-range assay kit (Q32850) on the Qubit 3 fluorimeter (Thermo Fisher Scientific) following manufacturer's instructions. DNA was also sized using a Genomic DNA ScreenTape Assay (5067-5366 [Reagents], 5067-5365 [Screentape]) on the TapeStation 2200 system (Agilent) following manufacturer's instructions. DNA samples with modal peaks >45 kb were carried forward for library construction and sequencing.

### Library construction

Library construction was conducted using the native barcoding kits (ONT SQK-NBD112.24 [V12 chemistry], SQK-NBD114.24 [V14 chemistry]) following manufacturer's instructions. Briefly, 400 ng DNA was aliquoted per barcoded sample for end and single-strand nick repair using NEBNext Ultra II End repair/dA-tailing Module and NEBNext FFPE Repair Mix (New England Biolabs M6630S, E7546S), with samples then being cleaned using AMPure XP Beads (Beckman Coulter) and 70% or 80% ethanol for V12 and V14 chemistry, respectively. Barcode ligation followed, using the barcodes provided and the NEB Blunt/TA Ligase Master Mix (New England Biolabs M0367L), with samples then being pooled together and cleaned as before. Finally, adapter ligation was conducted using the NEBNext Quick Ligation Module (New England Biolabs E6056S), with the library cleaned using AMPure XP Beads and the long-fragment buffer provided with the ONT library construction kit. Libraries were loaded onto MIN112 or MIN114 flowcells for V12 and V14 chemistries, respectively.

### Sequencing and adaptive sampling

All analysis scripts and CBL reference sequences used in this work are available on Zenodo (Horsfield 2024b). NCBI GenBank (https://www.ncbi.nlm.nih.gov/genbank/) reference sequence accession numbers for whole-genome assemblies include: *E. coli* DH5-α (NZ_JRYM01000009.1), *M. catarrhalis* (NZ_CP018059.1), *H. influenzae* (NZ_CP007470.1), *S. mitis* SK142 (NZ_JYGP01000001.1), *S. oralis* SK23 (NZ_LR134336.1), *S. pneumoniae* Spn23F (FM211187.1), *S. pneumoniae* R6 (NC_003098.1), and *S. pneumoniae* 110.58 (CP007593.1).

Sequencing was conducted using a MinION Mk1B instrument and a Dell Mobile Precision 7560 with an Intel Xeon processor and 128 GB RAM, and an NVIDIA RTX A5000 GPU with 16 GB GPU RAM running MinKNOW v22.12.7 (ONT UK) and MinKNOW core v5.4.3 (ONT UK). Local GPU base-calling was conducted using Guppy v6.4.6 (ONT UK) with the fast base-calling model and reads were rejected immediately if they did not align to the reference genome by setting “maxchunks” to 0 in the Readfish “.toml” file. For each new library, a control sequencing run was conducted for 1 h with no adaptive sampling with bulk capture, providing a “recording” for simulation playback.

Adaptive sampling was carried out using Readfish v0.0.10dev2 (Payne et al. 2021). Graph pseudoalignment was carried out using a custom fork from the Readfish GitHub repository (Horsfield 2024a). Readfish was installed using the “readfish.yml” file present in the GitHub repository by running the command “conda create -f readfish.yml.” During sequencing, Readfish was run using the command “sudo runuser minknow –c” /path/to/readfish targets ‐‐device [device] ‐‐experiment-name [name] ‐‐channels 1–256 ‐‐toml /path/to/toml ‐‐logfile [logfile] ‐‐port 9502 ‐‐graph [True/False] ‐‐align_threshold [threshold] ‐‐len_cutoff [cutoff].”

Adaptive sampling was used on channels 1–256 of the flowcell, with the remaining 256 channels run as controls without adaptive sampling. Linear alignment for adaptive sampling was carried out using Mappy v2.24 (https://pypi.org/project/mappy/). Sequencing was carried out for 24 h for each experiment, based on the sequencing time used in Payne et al. (2021), after which the run was terminated. No flowcell flushing or library reloading was conducted. Each sequencing experiment was run once. Metadata for all sequencing runs and samples, including the number of bases generated and aligned, the number of reads generated and aligned, and calculated enrichment, are available in Supplemental Data S1. This file also links each sequencing run archived on the European Nucleotide Archive (ENA; https://www.ebi.ac.uk/ena/browser/home) (Horsfield et al. 2024) to individual barcoded samples.

### Enrichment analysis

Enrichment analysis was based on analysis performed by Martin et al. (2022). Enrichment by the composition of the target *x*, denoted by *E*_*x*_, was calculated as described in Equation 1. Each flowcell was bioinformatically split into two halves; one half contained channels (a segment of a flowcell containing a nanopore) that were “adaptive” (using NAS), the other half contained channels that were “controls” (not using NAS). *E*_*x*_ was calculated as the fold increase in the proportion of read bases aligning to target sequence *x* in NAS channels, *a*, versus control channels, *c*:

(1) $$E_{x}=\frac{\left(\frac{N_{x,a}}{N_{\mathrm{total},a}}\right)}{\left(\frac{N_{x,c}}{N_{\mathrm{total},c}}\right)},$$

where *N*_*x*_ is the number of bases aligning to target sequence *x*, and *N*_total_ is the total bases sequenced in either adaptive (*a*) or control (*c*) channels. Using enrichment by composition enables results to be compared across sequencing runs, which may vary in the amount of data generated. If no aligning control reads were generated for a given library, *N*_*x*,*c*_ was set to 1 to avoid division by 0. A merged table of values used to calculated enrichment is present in Supplemental Table S1 (sheet “enrichment_calculation”), where enrichment is calculated as (bases_mapped_adaptive/bases_total_adaptive)/(bases_mapped_control/bases_total_control).

To calculate enrichment post-sequencing, all reads, including those passing and failing the Phred-score filter (Q-score ≥ 8), were aligned to a reference sequences using Mappy v2.24 using the custom script “analyse_RU.py.” Reads were aligned to specific reference sequences based on known isolates present within each sample (-t). All reads were used to avoid any potential biases introduced by read filtering, such as flow cell spatial effects, in the calculation of enrichment. Reads were split by channel (-c 1–256) to identify which reads were sequenced under NAS (channels 1–256) or control (channels 257–512) conditions. Reads aligning above a specified minimum identity threshold (84% identity within the aligned block, “-p 0.84”) were assigned as target reads, with the highest-identity alignment for multimapping reads being taken as the only alignment. Only regions of reads aligning to a reference sequence were included in enrichment calculations. Quartiles were generated from 100 bootstrapped samples of aligned reads (-bs 100).

### Serotype prediction

Serotype prediction was conducted using a customized version of PneumoKITy, which can be run using a single FASTQ file, as opposed to paired FASTQ files as in the original version, available at Zenodo (https://doi.org/10.5281/zenodo.10590659) (Horsfield 2024c). Reads were split using a custom script (split_by_channel.py) to generate files for reads sequenced under adaptive (channels 1–256) and control (channels 257–512) conditions (‐‐channels 1–256). PneumoKITy was run in “mix” mode using a minimum median-multiplicity value of 4 (-n 4) and a minimum *k*-mer percentage of 85% (-p 85) for reference CBL sequence matching.

### Assembly and quality control

All reads were first re-basecalled using Guppy v6.4.6 with the super-high accuracy model using the following command: “guppy_basecaller ‐‐compress_fastq ‐‐input_path [input_path] ‐‐save_path [output_path] ‐‐config dna_r10.4.1_e8.2_400bps_sup.cfg ‐‐device cuda:0 ‐‐recursive ‐‐barcode_kits SQK-NBD114-24 ‐‐enable_trim_barcodes ‐‐trim_adapters ‐‐trim_primers.” Reads were then assembled using metaFlye v2.9.2 (Kolmogorov et al. 2020) in “‐‐nano-raw” mode. We did not use the high accuracy “‐‐nano-hq” mode, as testing showed this was too stringent and resulted in no assembly being generated for some samples. Assembly quality was then analyzed using Inspector v1.2 (Chen et al. 2021), with reads mapped to respective assemblies, and assembly contigs mapped to respective reference sequences to identify errors. Errors were identified in contigs ≥50 bp in length (‐‐min_ contig_length_assemblyerror 50, ‐‐min_contig_length 50). BED files generated by Inspector, containing contig alignment and error positions on respective reference sequences, were visualized using a custom script (plotting_scripts/generate_linear_assembly_plot.R). Read alignment for coverage analysis was conducted using the custom script, “analyse_coverage.py,” using the original reads basecalled using Guppy's fast basecalling model. Alignment and read parsing settings were the same as “analyse_RU.py” described above. All alignment was carried out using Mappy v2.24. Assembly statistics are available in Supplemental Data S2.

### Nanopore sequencing simulation and analysis

Simulations of nanopore sequencing runs were conducted using bulk capture recordings from previous sequencing runs, as described on the Readfish GitHub repository (https://github.com/LooseLab/readfish). Results were analyzed using a custom script (analyse_unblocks.py). This script aligns reads to a specified target sequence using Mappy v2.24 and classifies them as either accepted or rejected by the adaptive sampling process. Reads that align to a target sequence and were accepted or rejected are classified as true positives and false negatives, respectively. Reads that did not align to a target sequence and were accepted or rejected are classified as false positives and true negatives, respectively. For all experiments described here, the reference sequence was the Spn23F Chromosome (‐‐ref data/cps/sequences/SP_ATCC700669.fasta) and the target sequence was the 23F CBL sequence (‐‐loci data/cps/split_cps/23F.fa).

For benchmarking of alignment speed, a bespoke simulation model was generated using NanoSim-H v1.1.0.2 (Yang et al. 2017; Břinda et al. 2018). Model training used FASTQ files from a V14 chemistry nanopore sequencing run containing 50%–50% dilutions of *S. pneumoniae* Spn23F and *E. coli* DH5-α, and their respective reference sequences (nanosim-h-train -i training_reads.fasta reference/genome.fasta output). Using this model, 500,000 simulated nanopore reads were generated (nanosim-h -n 500000 -p output reference_genome.fasta). Simulated reads were then split into true positive and true negative reads based on whether they originated from the 23F CBL using the custom script split_simulated.py, which parses reads simulated by NanoSim-H based on their original locus. Reads overlapping by at least 50 bp with the 23F CBL (position 303558–322212 bp within the Spn23F Chromosome) were classified as true positives (‐‐pos 303558–322212 ‐‐min-overlap 50). CBL sequences (updated_cps.fasta, N = 106) were indexed using minimap2 v2.26 (Li 2018) and Bifrost v1.2.0 (Holley and Melsted 2020) with *k* = 19. The time taken to align all 500,000 simulated reads for Mappy v2.24 and graph pseudoalignment was measured using a custom script (simulate_readuntil.py), which parses the start of each read, with length defined by Poisson sampling (“‐‐avg-poi 180,” based on Payne et al. [2021]). This fragment is then aligned using both Mappy and graph pseudoalignment, with alignment timed using the Python “timeit” module. Graph pseudoalignment was run using minimum read identity 75% (‐‐id 0.75) and minimum read length 50 bp (‐‐min-len 50). Mappy was run with default parameters. Alignment accuracy was measured based on whether a read was accepted or rejected, depending on whether it originated from the 23F CBL or not. Comparisons were carried out on a server cluster with dual processor x86-64 nodes, running CentOS v8.2.

### Pseudoalignment simulation

Pseudoalignment simulations proceed as follows. A specified number of target and nontarget sequences of given lengths are generated by random sampling of DNA bases. Constituent *k*-mers of these sequences are then generated, and reads with specified mutation rates are simulated from target sequences. Read *k*-mers are then matched back to the respective target and nontarget *k*-mer sets, enabling calculation of recall and precision, respectively. The code for this process can be found in the “kmer_simulation.R” script.

### Software availability

Code for GNASTy is available at Zenodo (https://zenodo.org/records/13358697) (Horsfield 2024a) and GitHub (https://github.com/bacpop/readfish/tree/graph_alignment_bifrost) under the GPL-3.0 license. All analysis scripts used in this work are available at Zenodo (https://zenodo.org/records/12636613) (Horsfield 2024b) and GitHub (https://github.com/bacpop/adaptive_sampling_scripts) under the GPL-3.0 license. This repository also contains 106 *S. pneumoniae* CBL sequences and associated sources (updated_cps.fasta, updated 19th December 2022) used as reference sequences for NAS. The updated version of PneumoKITy used in this manuscript is also available at Zenodo (https://doi.org/10.5281/zenodo.10590659) (Horsfield 2024c) under the GPL-3.0 license. All code is also available as Supplemental Code.

## Data access

All raw and processed sequencing data generated in this study have been submitted to the European Nucleotide Archive (ENA; http://www.ebi.ac.uk/ena) under accession number PRJEB72455.

## Supplemental Material

Supplement 1

Supplement 2

Supplement 3

Supplement 4
