# Supplementary material for: Optimizing nanopore adaptive sampling for pneumococcal serotype surveillance in complex samples using the graph-based GNASTy algorithm
Source: Genome Res. 2025 Apr;35(4):1025–40. doi: 10.1101/gr.279435.124 (PMC12047183; doi:10.1101/gr.279435.124)
Supplement: Supplement 2 [file Supplemental_Material.pdf]

A Supplementary Figures

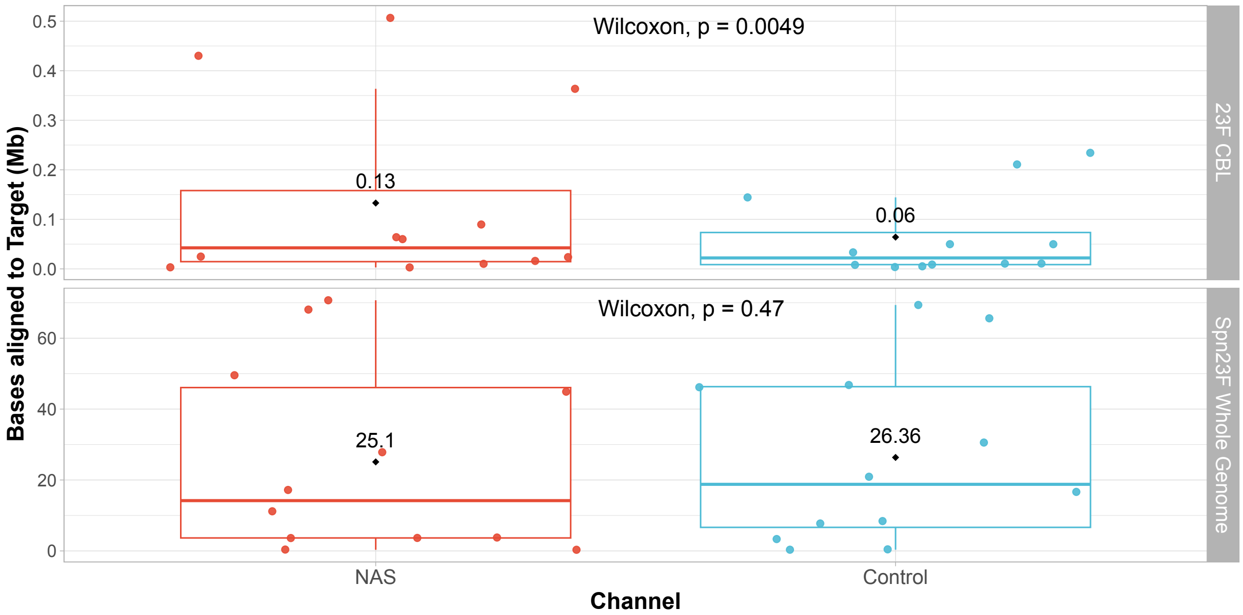

Supplementary Figure 1: Absolute yield in megabases (Mb) of bases aligning to the whole genome of Spn23F **(top)** and 23F CBL **(bottom)**. Each data point represents the enrichment of the Spn23F whole genome or 23F CBL found within each library. Distributions from control and NAS channels were compared using a paired Wilcoxon test.

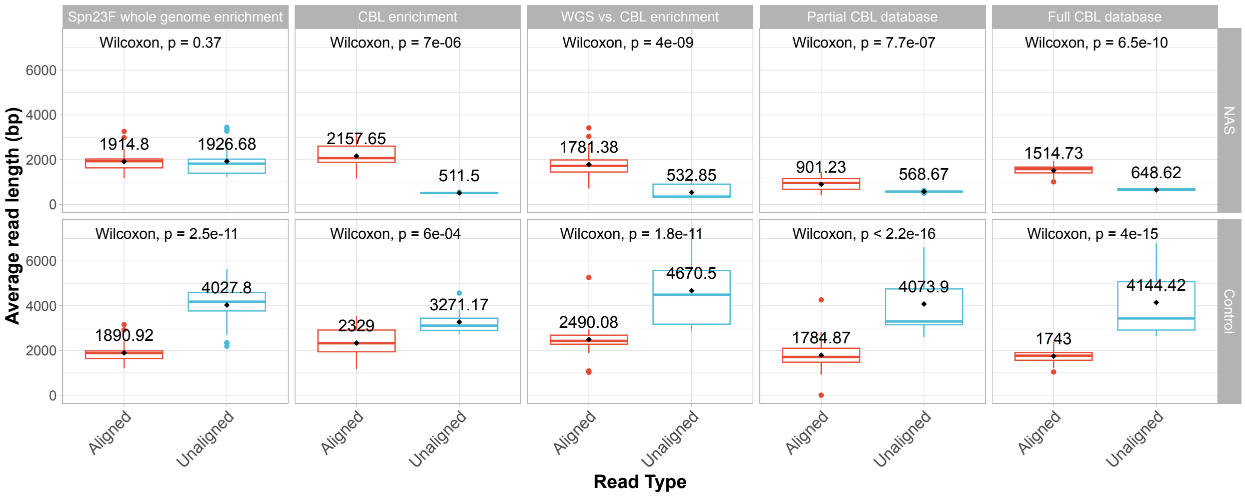

Supplementary Figure 2: Comparison of average read lengths between reads aligned and unaligned to target sequences between NAS and Control channels for all experiments with size selection. Boxplots represent average read lengths aligned or unaligned to a target with a given library. Distributions from aligned and unaligned reads were compared using a Wilcoxon test. Further information on the library composition in each experiment is available in Supplemental Data S1.

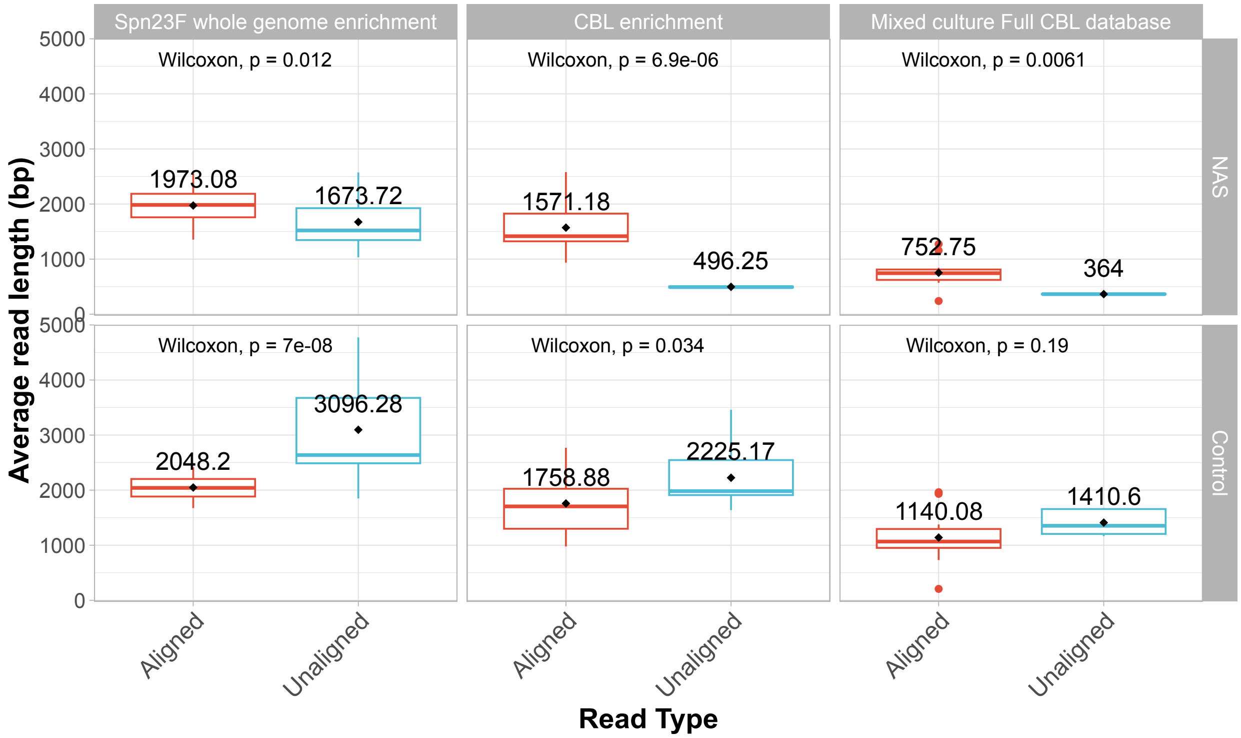

Supplementary Figure 3: Comparison of average read lengths between reads aligned and unaligned to target sequences between NAS and Control channels for all experiments without size selection. Boxplots represent average read lengths aligned or unaligned to a target with a given library. Distributions from aligned and unaligned reads were compared using a Wilcoxon test. Further information on the library composition in each experiment is available in Supplemental Data S1.

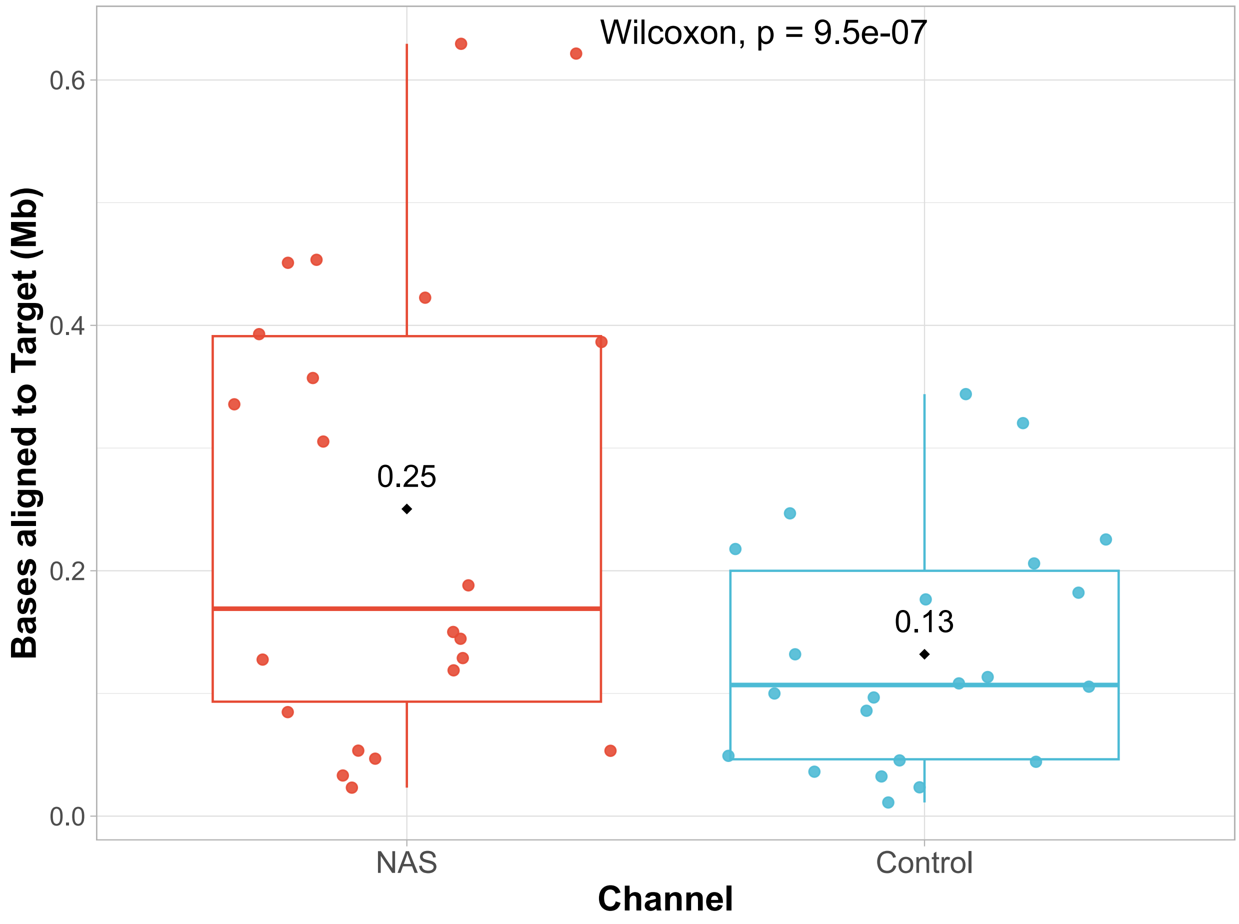

Supplementary Figure 4: Absolute yield in megabases (Mb) of bases aligning to the individual CBL in multi-serotype samples. Each data point represents the enrichment of a single CBL found within each library. Distributions from control and NAS channels were compared using a paired Wilcoxon test.

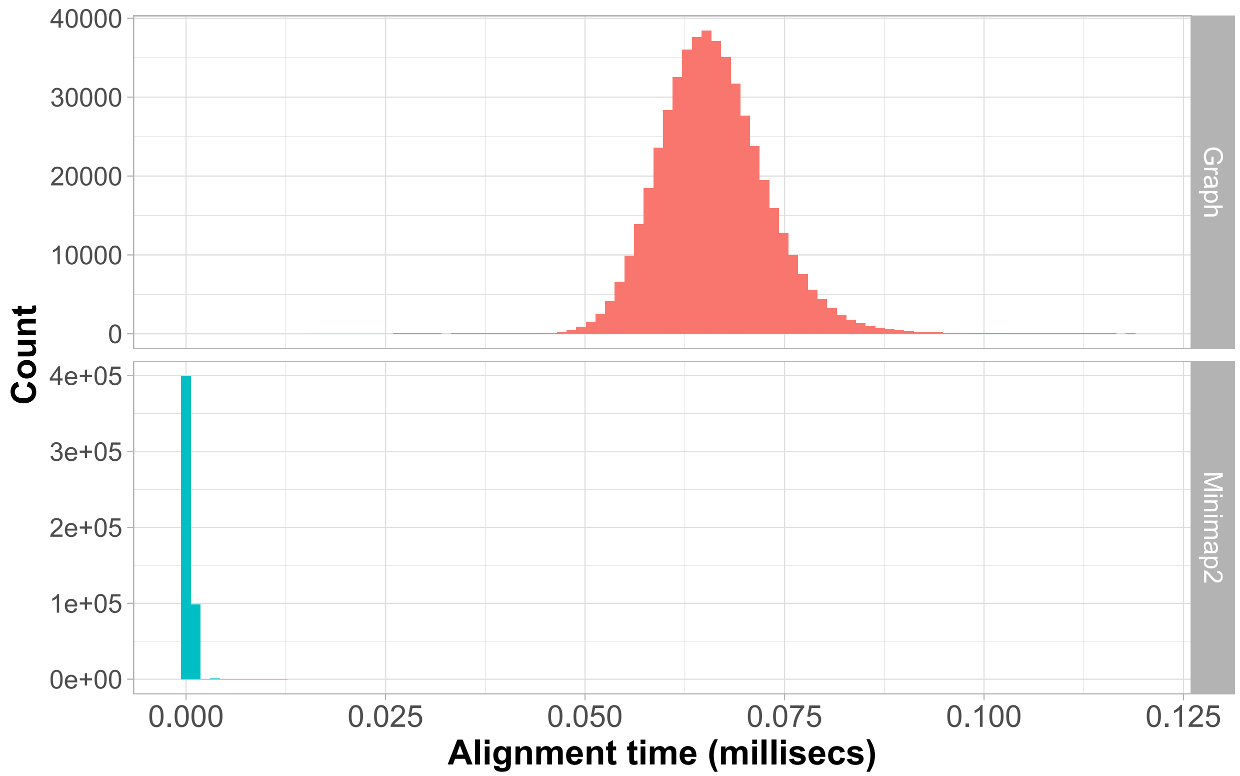

Supplementary Figure 5: Alignment speed comparison between graph pseudoalignment in GNASTy and minimap2.

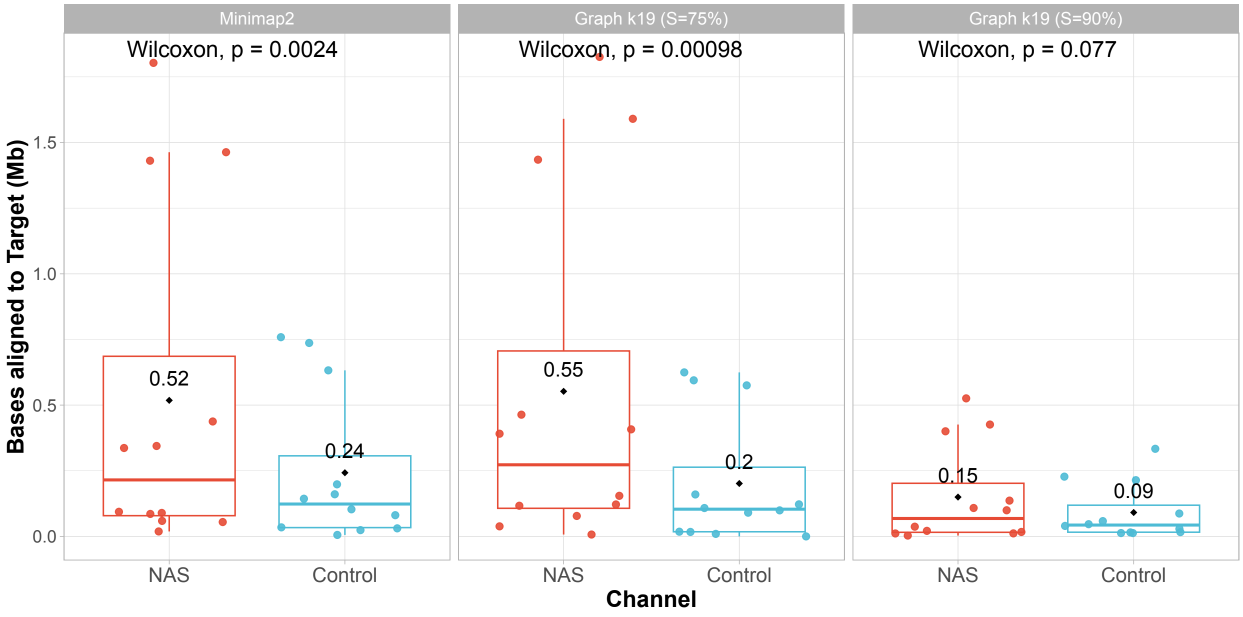

Supplementary Figure 6: Absolute yield in megabases (Mb) of bases aligning to the 23F CBL when aligning to a partial CBL database using minimap2 and GNASTy. Each data point represents the enrichment of the 23F CBL found within each library. Distributions from control and NAS channels were compared using a paired Wilcoxon test.

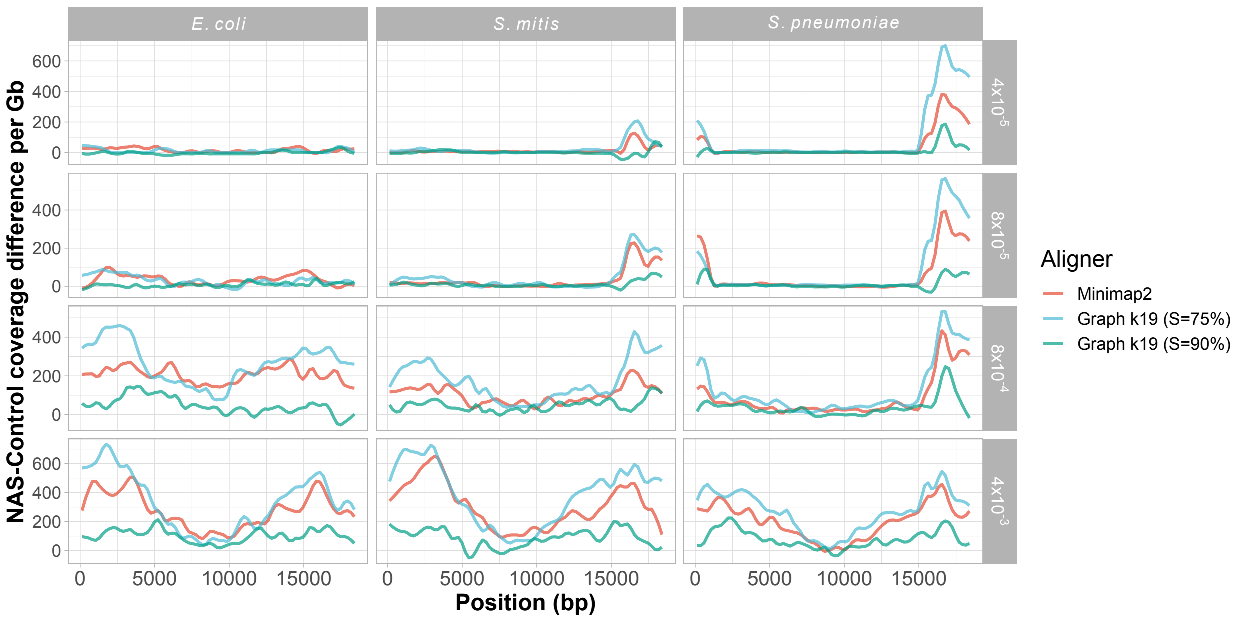

Supplementary Figure 7: Normalised coverage difference between NAS and control channels across 23F CBL using a partial CBL reference database using minimap2 and GNASTy. NAS-control coverage difference per gigabase (Gb) calculated by normalising the read coverage for each locus by the amount of data generated (in Gb) for each respective sample and channel, and then negating the normalised coverage for control channels from NAS channels for each locus. Columns describe the nontarget species. Rows describe the proportion of target DNA present in the sample.

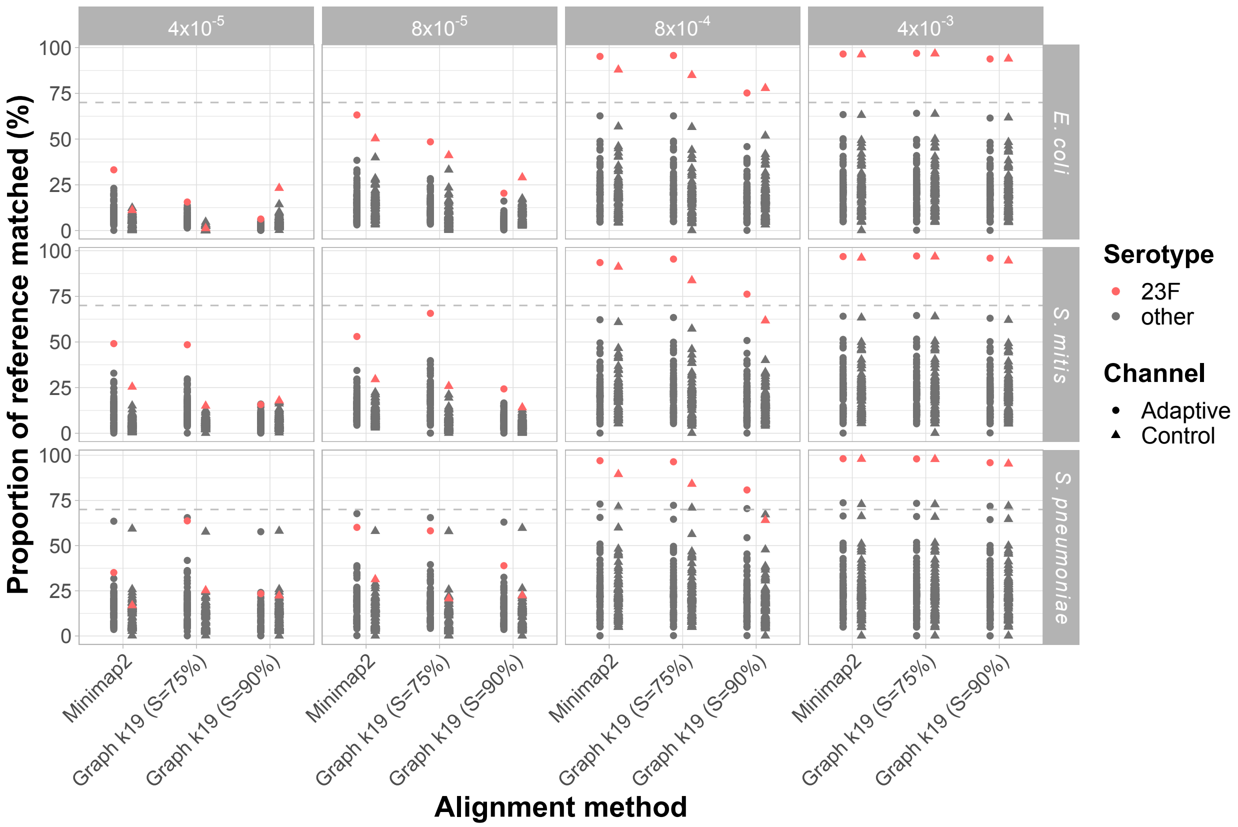

Supplementary Figure 8: Serotype 23F prediction using reads aligning a to partial CBL reference database using minimap2 and GNASTy. Y-axis describes the proportion of reference CBL *k*-mers matched by PneumoKITy (Sheppard *et al.* 2022), with each data point describing an individual CBL. The lower limit of matching *k*-mers used by PneumoKITy (70%) to identify as CBL as present is marked by the grey dotted line. Predictions for the 23F CBL are highlighted in red. Shapes described the channel type (adaptive or control). Columns describe the 23F CBL DNA proportion in the sample. Rows describe the nontarget species mixed with Spn23F. Sub-serotypes (e.g. 6A-I, 6A-II etc.) were removed from data to avoid redundancy.

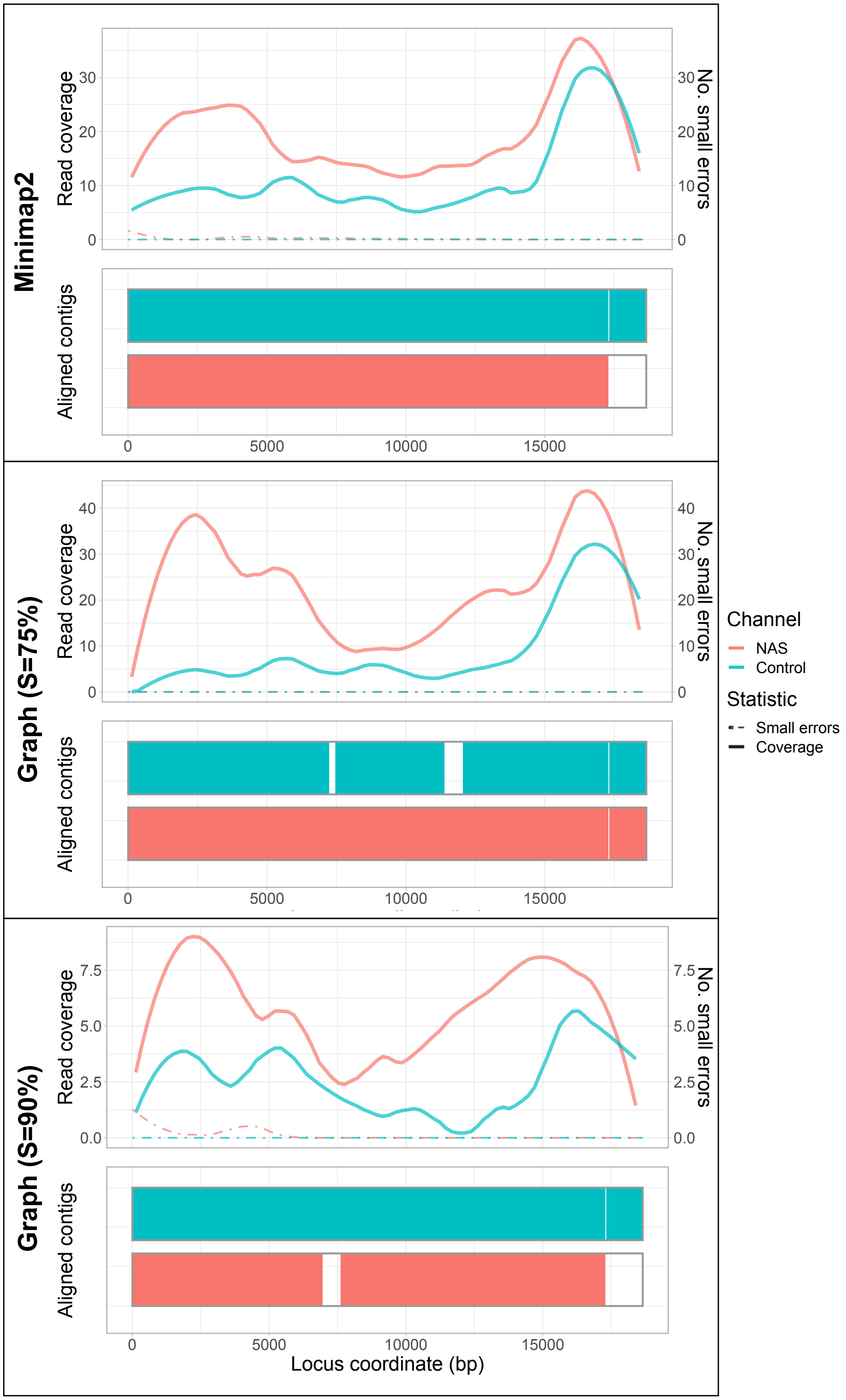

Supplementary Figure 9: Spn23F CBL assembly comparison across alignment methods using a partial CBL database during NAS using minimap2 and GNASTy. Each panel describes a 23F CBL assembly generated from 0.1 Spn23F dilutions with *S. mitis*. For each panel, the top plot shows the read coverage (solid), defined as the absolute number of bases aligning to a locus, and number of small errors ($\leq$ 50 bp, dashed), whilst the bottom plot shows aligned contigs (colours) and large errors ($>$ 50 bp) in each assembly.

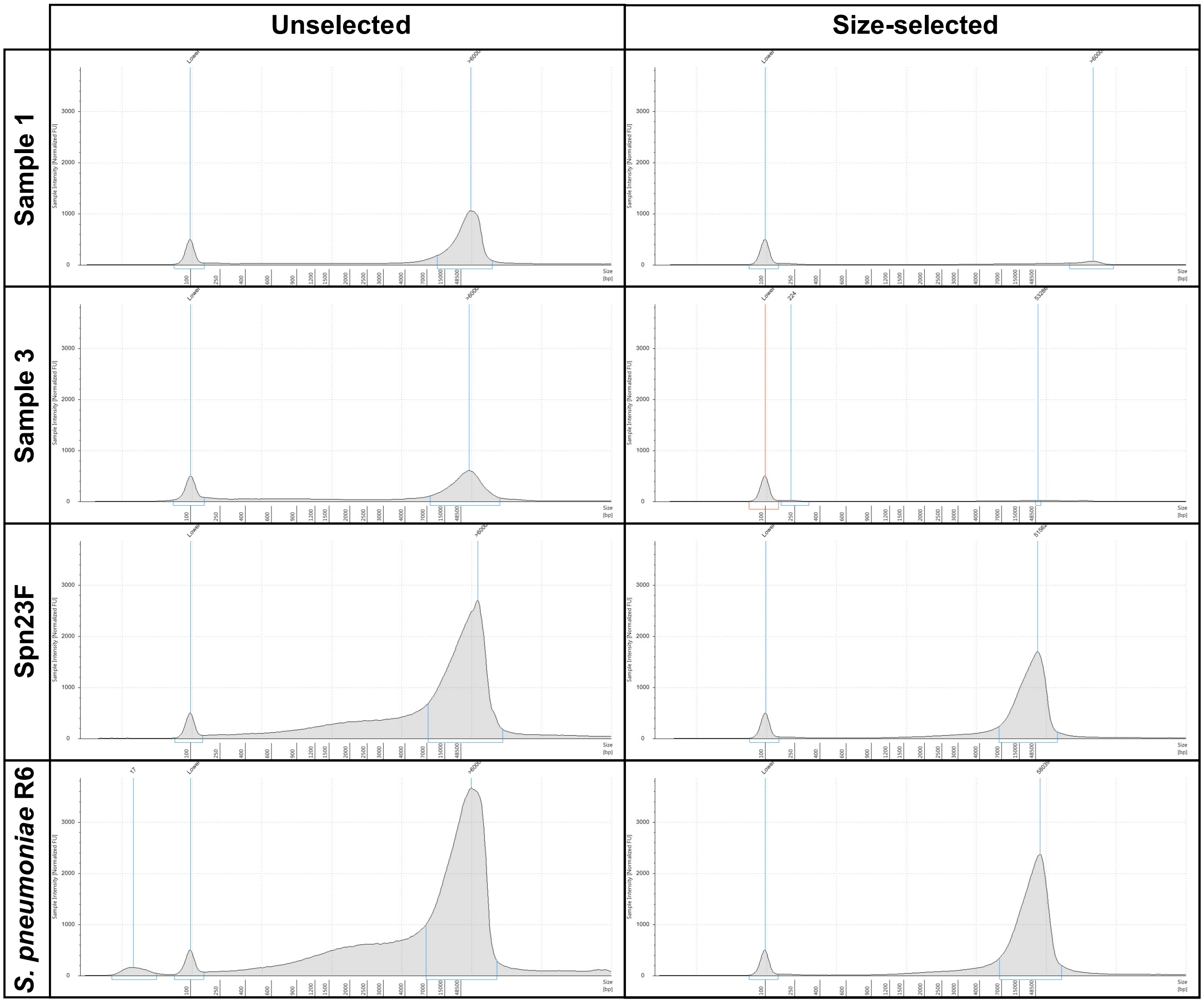

Supplementary Figure 10: Tapestation images for unselected and size-selected samples. Mixed cultures from nasopharyngeal samples are denoted ‘Sample X’. DNA extractions from single isolate cultures, Spn23F and *S. pneumoniae* R6, were included as positive controls. Y-axis denotes normalised flourescent units (fU). X-axis denotes DNA fragment size (bp). Starting DNA concentrations for size selection: Sample 1: 100 ng/µL, Sample 3: 40 ng/µL, Spn23F: 100 ng/µL, *S. pneumoniae* R6: 100 ng/µL

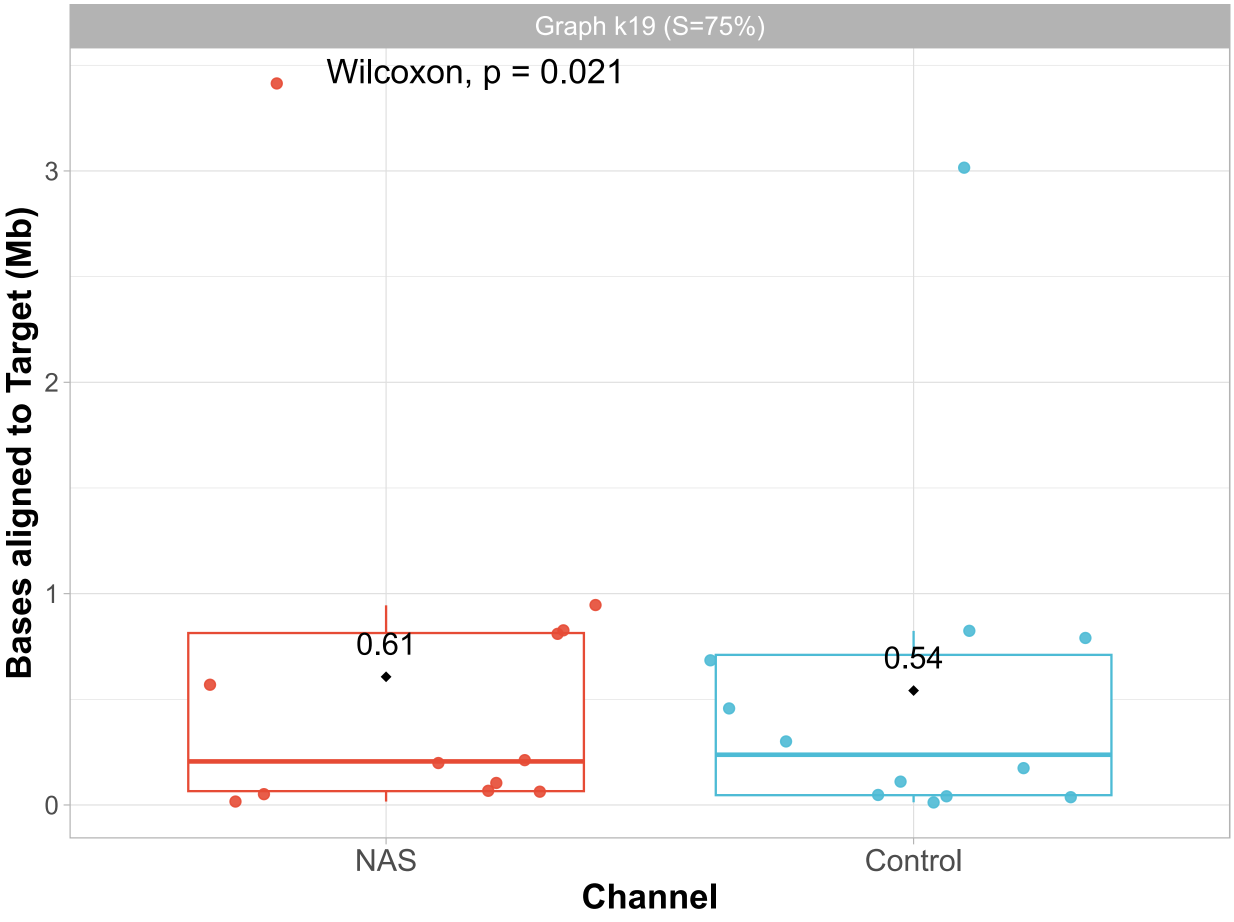

Supplementary Figure 11: Absolute yield in megabases (Mb) of bases aligning to the 23F CBL when aligning to a full CBL database for mock nasopharyngeal samples using minimap2 and GNASTy. Each data point represents the enrichment of a CBL identified by PnumoKITY as present within each library. Distributions from control and NAS channels were compared using a paired Wilcoxon test.

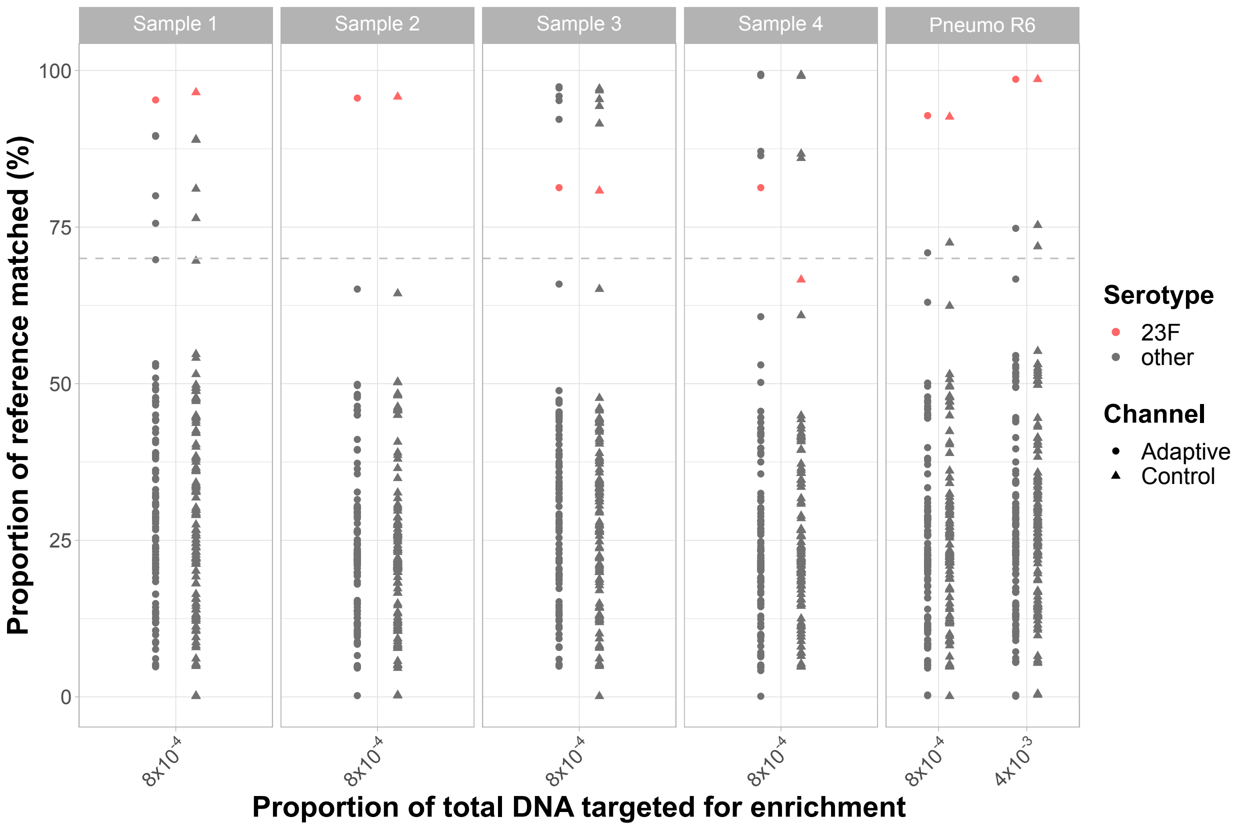

Supplementary Figure 12: Serotype 23F prediction from mock nasopharyngeal microbiomes using graph pseudoalignment in GNASTy for enrichment. Y-axis describes the proportion of reference CBL *k*-mers matched by Pneumokity (Sheppard *et al.* 2022), with each data point describing an individual CBL. The lower limit of matching *k*-mers used by PneumoKITy (70%) to identify as CBL as present is marked by the grey dotted line. Predictions for the 23F CBL are highlighted in red. Shapes described the channel type (adaptive or control). X-axis describes the 23F CBL DNA proportion in the sample. Columns describe the nasopharyngeal mixed culture (denoted as ‘Sample X’) or *S. pneumoniae* R6 sample mixed with Spn23F. Subtypes based on *wzy* variation (e.g. 6A-I, 6A-II etc.) were removed from data to avoid redundancy.

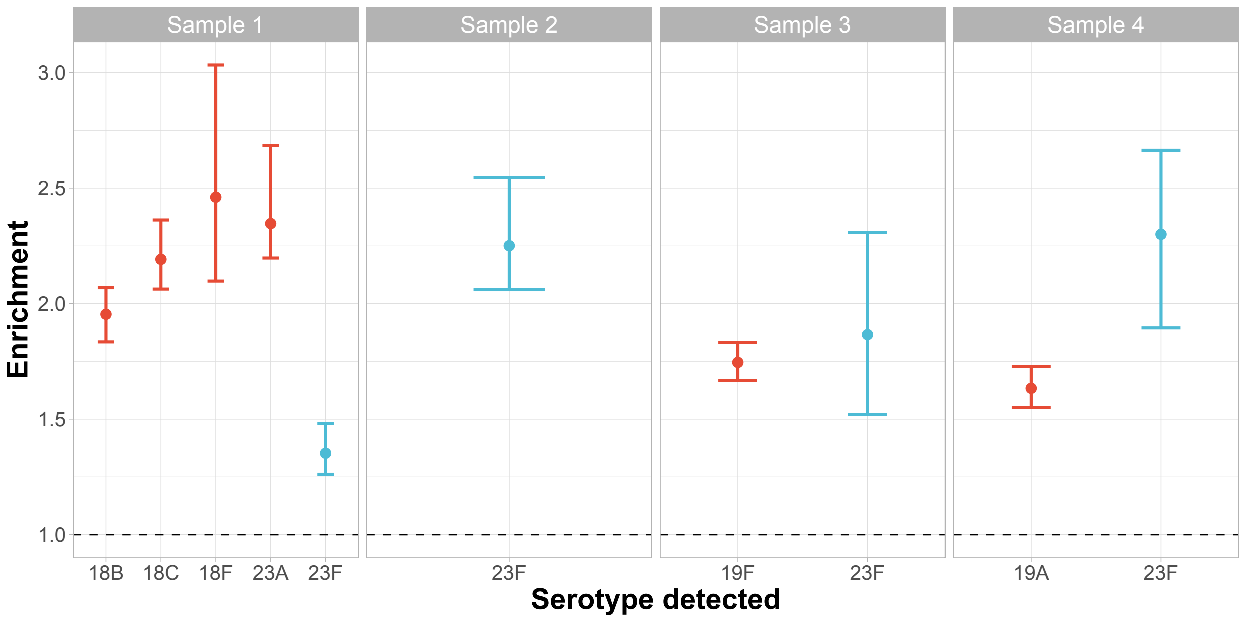

Supplementary Figure 13: Enrichment of multiple CBL from mock nasopharyngeal microbiomes using GNASTy. Serotypes shown are those with $\geq 70\%$ of their respective reference sequence mapped to by reads as calculated by PneumoKITY in Supplementary Figure 12. Bar ranges are inter-quartile range of enrichment from 100 bootstrap samples of reads. Columns describe the nasopharyngeal mixed culture (denoted as 'Sample X'). Results for the 23F CBL are highlighted in blue. Horizontal dashed line describes enrichment = 1 i.e. no enrichment has occurred.

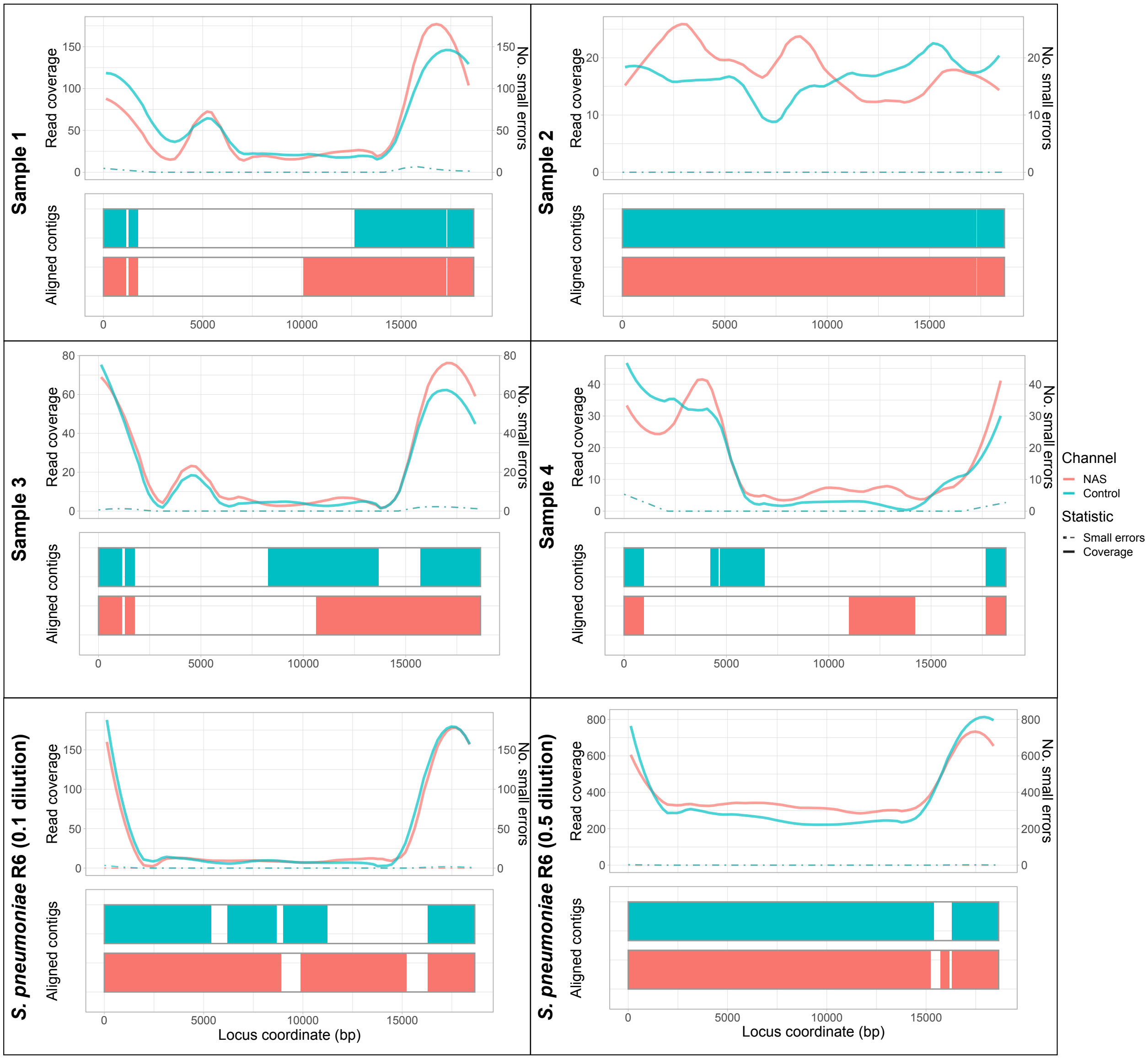

Supplementary Figure 14: Spn23F CBL assembly from mock nasopharyngeal microbiomes using graph pseudoalignment in GNASTy ($S = 75\%$) aligning to a full CBL database during NAS. Each panel describes a 23F CBL assembly generated from 0.1 Spn23F spike into mixed cultures (for 'Sample X') or 0.1 and 0.5 Spn23F spikes into *S. pneumoniae* R6. For each panel, the top plot shows the read coverage (solid), defined as the absolute number of bases aligning to a locus, and number of small errors (≤ 50 bp, dashed), whilst the bottom plot shows the aligned contigs (colours) and large errors (> 50 bp) in each assembly.

B Supplementary Tables

Supplemental Data S1: Per-sample statistics for: the number of bases mapped, the number of total bases, the number of reads mapped, the number of total reads, average lengths of mapped reads, average lengths of total reads, enrichment and flowcell yield. This table also includes a tab which details the statistics used to calculate per-sample enrichment.

Supplemental Data S2: Per-sample statistics for: assembly contiguity, read-to-assembly accuracy and assembly-to-reference accuracy.

Supplementary Table 1: Computational comparison of minimap2 index and graph building using $k = 19$ (referred to as 'graph') from 106 CBL sequences. Tools were run using 16 threads.

Tool	Runtime (seconds)	Peak memory (MB)	Index size (MB)
minimap2	0.3	20	5.01
graph	9.08	89	2.16

Supplementary Table 2: Number of reads aligning to 23F CBL when enriching for CBL using NAS.

Nontarget species	Channel	Target proportion	Reads mapped
E. coli	NAS	4×10^{-5}	1
		8×10^{-5}	3
		8×10^{-4}	44
		4×10^{-3}	203
	Control	4×10^{-5}	1
		8×10^{-5}	2
		8×10^{-4}	15
		4×10^{-3}	68
S. mitis	NAS	4×10^{-5}	2
		8×10^{-5}	6
		8×10^{-4}	37
		4×10^{-3}	301
	Control	4×10^{-5}	4
		8×10^{-5}	3
		8×10^{-4}	22
		4×10^{-3}	101
S. pneumoniae	NAS	4×10^{-5}	35
		8×10^{-5}	27
		8×10^{-4}	42
		4×10^{-3}	215
	Control	4×10^{-5}	10
		8×10^{-5}	8
		8×10^{-4}	19
		4×10^{-3}	78

C Supplemental Material

C.1 Optimising alignment identity threshold for calculation of enrichment.

To calculate enrichment, reads are aligned to the Spn23F reference twice: once during NAS, and once in post-sequencing analysis using a custom script (see Results, Section 'NAS performance depends on microbiome composition'). Therefore, calculatiFon of enrichment is dependent on the accuracy of read alignment both during NAS and in post-sequencing analysis. Alignment accuracy during NAS is dependent on the base-caller and aligner which are set *a priori*. However, in post-sequencing analysis, alignment accuracy is dictated by the minimum alignment identity between reads and the reference sequence. Minimum alignment identity controls whether a read aligning to the reference sequence is accepted as a true target read. A cutoff that is too low will incorrectly identify nontarget reads as target, whilst one that is too high may remove target reads that have high numbers of errors, with both scenarios resulting in lower estimates of enrichment.

We tested the effect of varying the minimum alignment identity on the calculated Spn23F enrichment across all mixed samples, in order to set a cutoff to be used throughout this work. We generated mock communities containing mixtures of genomic DNA of *S. pneumoniae* strain Spn23F (Croucher *et al.* 2009) with that of closely and distantly related nontarget species. Spn23F DNA was mixed with DNA from species from different phyla (*E. coli* and *M. catarrhalis* + *H. influenzae*), the same genus but different species (*S. mitis* and *S. oralis*) and the same species (*S. pneumoniae* R6 and *S. pneumoniae* 110.58) (Supplementary Table 3). Mixture of Spn23F with *M. catarrhalis* + *H. influenzae* was used to simulate the composition of the nasopharynx, where co-carriage with these three species is common (Kovács *et al.* 2020; Dunne *et al.* 2018; Chochua *et al.* 2016). Titrations ranged from 0.001-0.5 proportions ($0.1\% - 50\%$) of Spn23F DNA, to test the effect of lower target inputs on enrichment. Sequencing was carried out using the V12 sequencing chemistry using the Guppy v5.0.15 base-caller.

We found that enrichment was insensitive to minimum alignment identity in the range 80-90% (Supplementary Figure 15). At $> 90\%$ minimum alignment identity, enrichment was more variable (Supplementary Figure 16) and resulted in depletion of Spn23F DNA in many samples between 92-100%, where enrichment falls below one which should be avoided. This effect was independent of concentration or contaminating species, and is indicative of rejection of correct Spn23F reads containing sequencing errors. Based on these results, a cutoff of 84% minimum alignment identity was used for all further analysis, as this is equivalent to the minimum Phred score (Q8) used by the Guppy base-caller in fast mode to identify failed reads.

Supplementary Table 3: Experimental set-up of Spn23F whole genome enrichment. Spn23F DNA was diluted in proportion with nontarget DNA from members of different genera (Non-*Streptococcus*), the same genera (*Streptococcus*) and the same species (*S. pneumoniae*).

Nontarget type	Nontarget species	Spn23F proportion
Non-*Streptococcus*	*E. coli*	0.001 (0.1%)
		0.01 (1%)
		0.1 (10%)
		0.5 (50%)
	M. catarrhalis + *H. influenzae*	0.01 (1%)
		0.1 (10%)
		0.5 (50%)
Streptococcus	*S. mitis*	0.01 (1%)
		0.1 (10%)
		0.5 (50%)
	S. oralis	0.01 (1%)
		0.1 (10%)
		0.5 (50%)
Streptococcus pneumoniae	*S. pneumoniae* R6	0.001 (0.1%)
		0.01 (1%)
		0.1 (10%)
		0.5 (50%)
	S. pneumoniae 110.58	0.01 (1%)
		0.1 (10%)
		0.5 (50%)

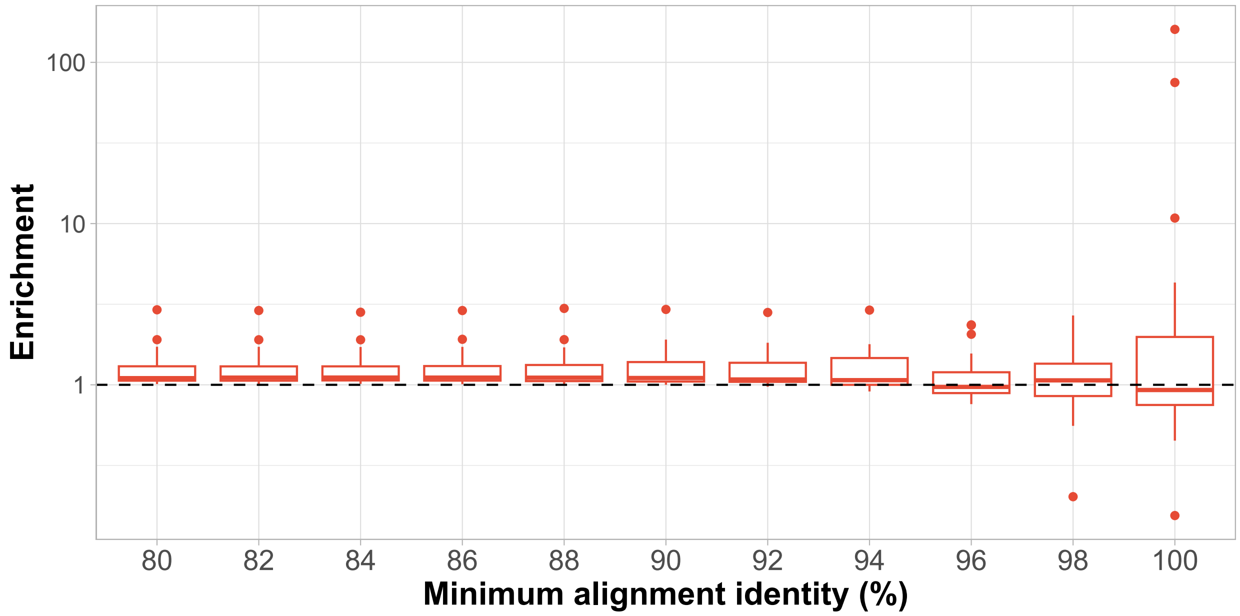

Supplementary Figure 15: Effect of minimum alignment identity on Spn23F whole genome enrichment. Boxplots represent distributions of enrichment values of Spn23F whole genome in all samples. Dashed line describes enrichment = 1 i.e. no enrichment has occurred.

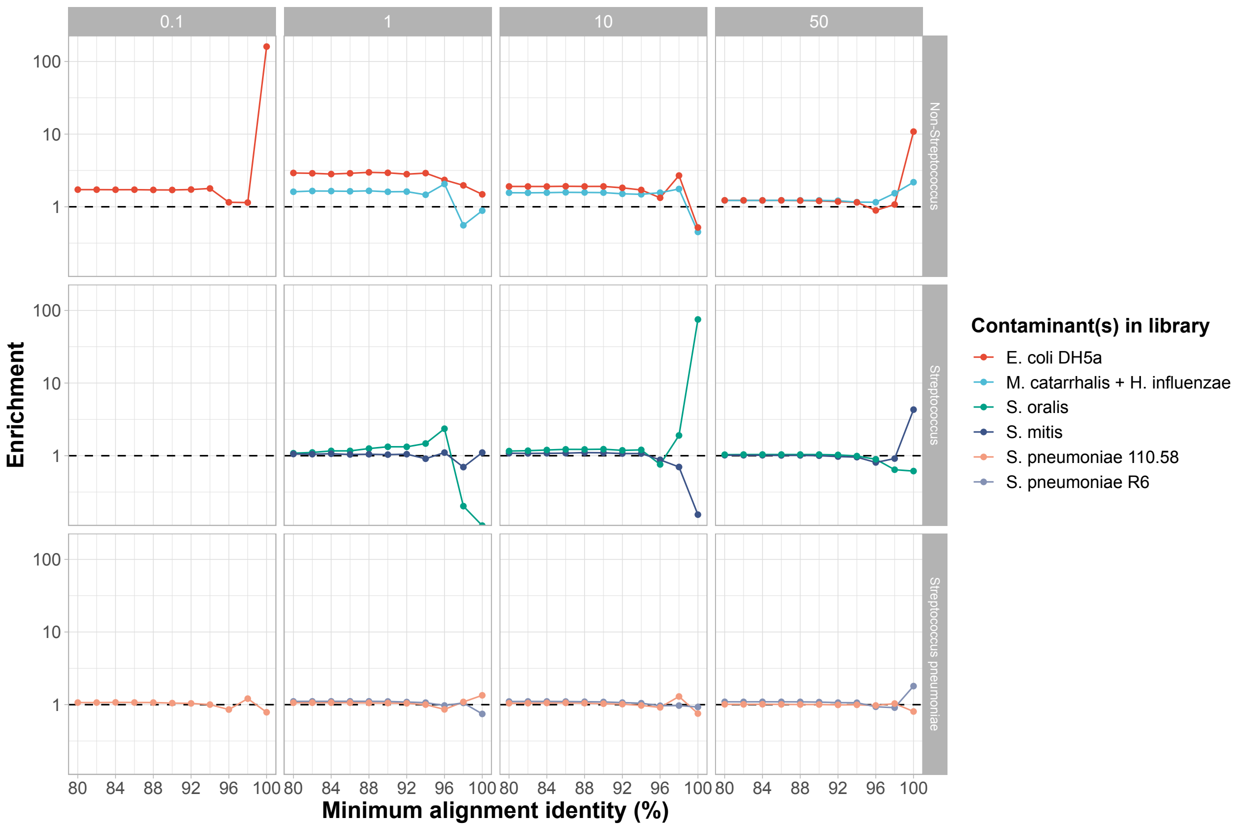

Supplementary Figure 16: Effect of minimum alignment identity on Spn23F whole genome enrichment across sample concentrations and contaminating species. Lines connect the same sample with different minimum alignment identity thresholds. Columns indicate the % Spn23F DNA in the sample, rows indicate the nontarget type. Colours describe the species of the nontarget species. No library was sequenced at 0.1% Spn23F DNA with a *Streptococcus* nontarget species. Dashed line describes enrichment = 1 i.e. no enrichment has occurred.

C.2 Removal of short DNA fragments from libraries using size selection increases target enrichment.

Short DNA fragments are sequenced too quickly to be rejected by NAS, resulting in reduced target enrichment if many short nontarget sequences are present in a sample. Martin *et al.* (2022) showed that removal of short fragments improves both enrichment and absolute yield of target DNA. To determine whether this effect could be reproduced, size selection was used on all libraries in Supplementary Table 3, in which DNA fragments < 10 kb were removed from each sample.

Comparison of read length distributions before and after size selection highlighted read length proportions were shifted to higher values for accepted reads in adaptive channels and all reads in control channels (Supplementary Figure 17). Mean and median read lengths were still below 10 kb (Supplementary Table 4), indicating size selection was effective but imperfect. The size of rejected reads was similar between both experimental conditions, indicating that size selection had no impact on rejection speed, with faster read rejection expected to result in shorter rejected reads (Payne *et al.* 2021). size selection had a small but significant effect on enrichment (Supplementary Figure 18), increasing average enrichment from 1.31 to 1.5. Overall, size selection increased the size of accepted reads, although the observed effect on enrichment was small.

Supplementary Table 4: Read length statistics for Spn23F whole genome enrichment. All statistics are in basepairs.

Channel type	Read type	Experiment	Mean	Lower Quartile	Median	Upper Quartile
Adaptive	Accepted	Unselected	1863	589	1027	2035
		Size selection	2447	780	1444	3076
	Rejected	Unselected	1378	1222	1360	1522
		Size selection	1360	1203	1333	1489
Control	All	Unselected	2407	693	1380	2813
		Size selection	3212	993	2102	4145

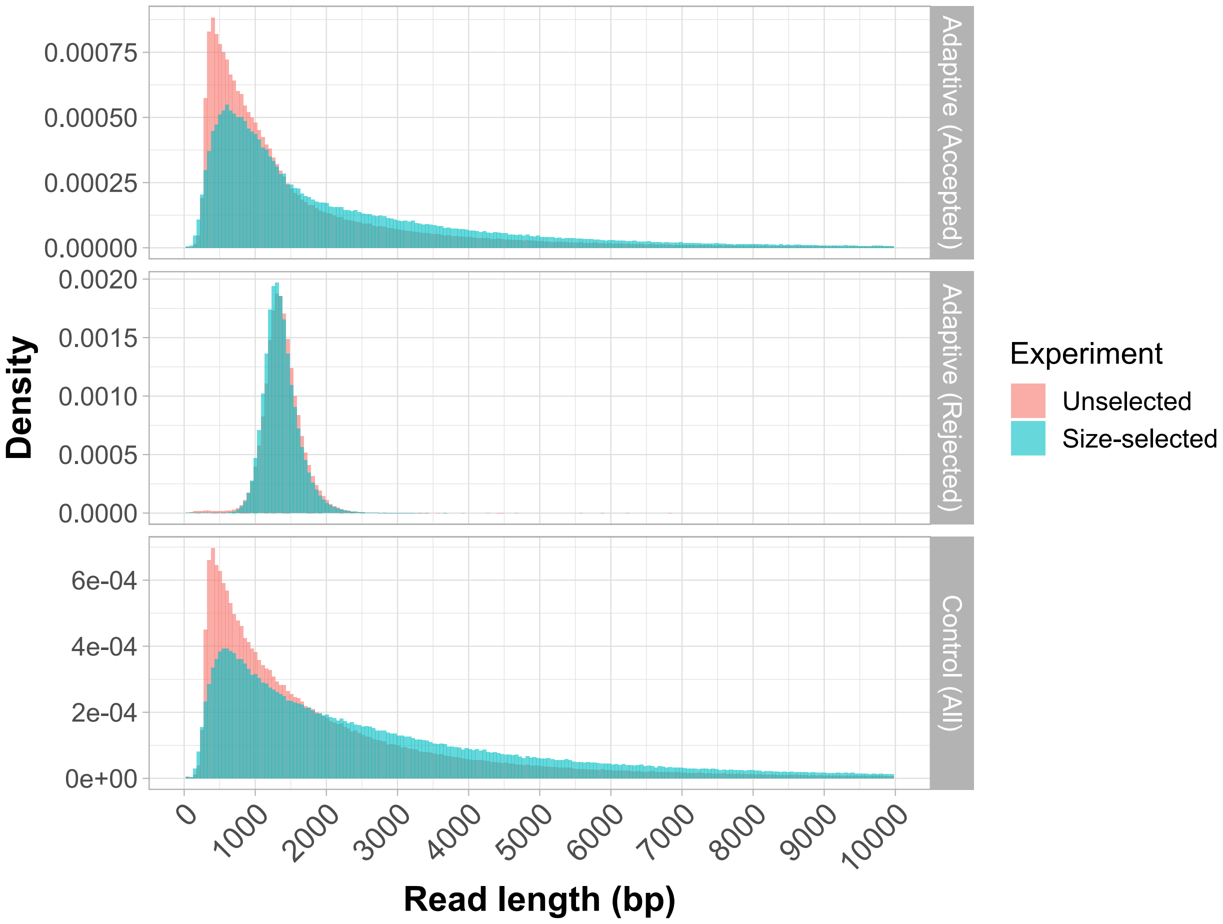

Supplementary Figure 17: Effect of size selection on read length distributions for Spn23F whole genome enrichment. Histograms for reads accepted (**top**) and rejected (**middle**) by NAS in adaptive channels, and all reads in control channels (**bottom**).

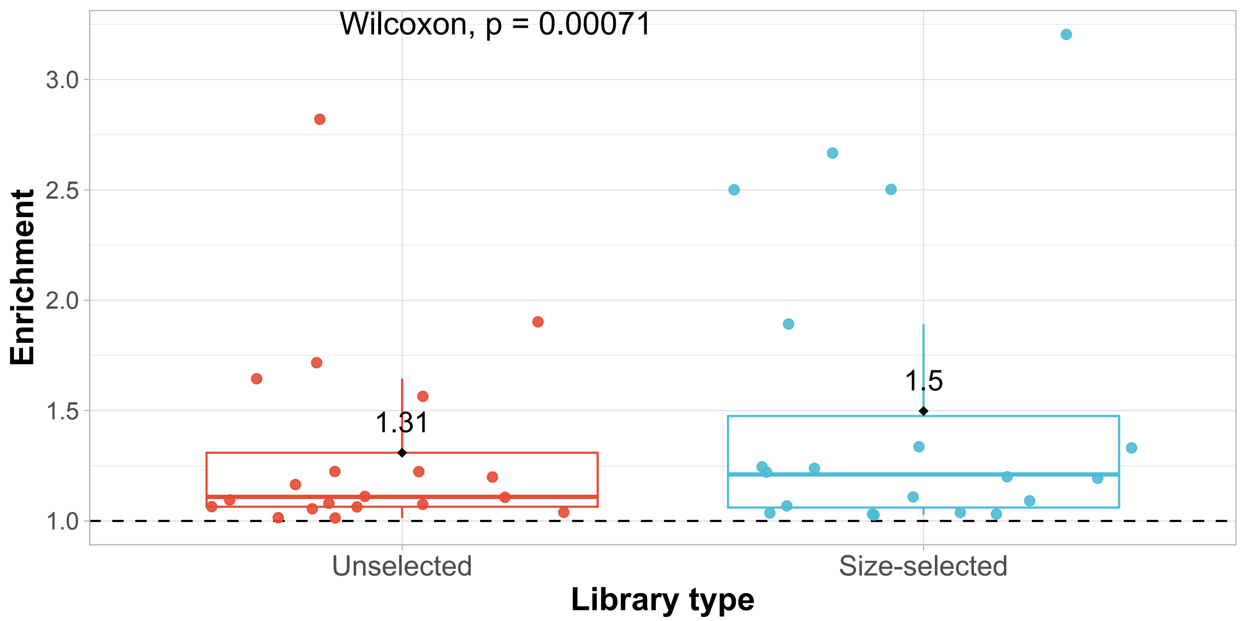

Supplementary Figure 18: Enrichment comparison of Spn23F genome with and without size selection, which removed DNA fragments < 10 kb in length. Each data point represents the enrichment of Spn23F in a single barcoded library. Dashed line describes enrichment = 1 i.e. no enrichment has occurred. Black points highlight distributions means. Distributions were compared using a paired Wilcoxon test.

We additionally tested the effectiveness of size selection on enrichment of CBL. Previously, Viehweger *et al.* (2023) showed that enrichment of loci, in this case AMR genes, was possible using NAS, although performance was dependent on the library fragment length. Paradoxically, the authors suggested that when enriching for genes, libraries with a greater proportion of short fragments provide better enrichment, in contrast to our observation that size selection improves enrichment. Viehweger *et al.* (2023) postulated that this was due to the higher probability of a short target locus being found in the middle of a longer read, rather than at the start, which is the region aligned during NAS. In longer DNA fragments, it is therefore more likely that the start region of a read will align outside of the target locus, and will therefore be incorrectly rejected, despite the target sequence being present later in the read. CBL contain ~ 20 genes, and so are substantially longer than individual genes. Therefore it is unknown whether read length has the same effect on CBL enrichment.

To determine the effectiveness of CBL enrichment with size selection, libraries containing single isolate DNA from *S. pneumoniae* strains were generated, as well as mixtures with Spn23F DNA with a variety of serotypes and genotypes (Supplementary Table 5). Strains with the same serotype but different genotypes, and strains with the same strain background but different serotypes, were included to determine whether CBL are sufficiently different from the rest of the genome and each other to be effectively enriched. Sequencing was carried out using the Guppy v6.3.8 base-caller, which has higher base-calling speed over Guppy v5.0.15 used in the previous section according to the ONT release notes, with V12 sequencing chemistry.

Supplementary Table 5: Experimental set-up of CBL enrichment. All strains are members of the *S. pneumoniae* species. Samples with 50% proportion were diluted with Spn23F DNA.

Strain name	Strain genotype	Serotype	Proportion
Spn23F	GPSC16	23F	100%
8140	GPSC16	19A	100%
			50%
Mal M6	GPSC16	19F	100%
			50%
Tw01_0057	GPSC1	19F	100%
			50%
99_4038	GPSC3	03	100%
			50%
K13_0810	GPSC23	06B	100%
			50%
R6	GPSC622	NT	50%

We observed longer reads in size-selected than unselected libraries for accepted reads in adaptive channels, and all reads in control channels (Supplementary Figure 19, Supplementary Table 6), as seen before with whole genome enrichment. However, accepted and rejected reads in adaptive channels for CBL enrichment were notably shorter than those in whole genome enrichment, whilst control channel read lengths were similar (Supplementary Table 4). This effect may be due to accepting of shorter reads when aligning to shorter targets, as observed in Viehweger *et al.* (2023). Size selection also had a positive significant effect on enrichment when targeting CBL, increasing the average enrichment from 3.4 to 4.8 (Supplementary Figure 20).

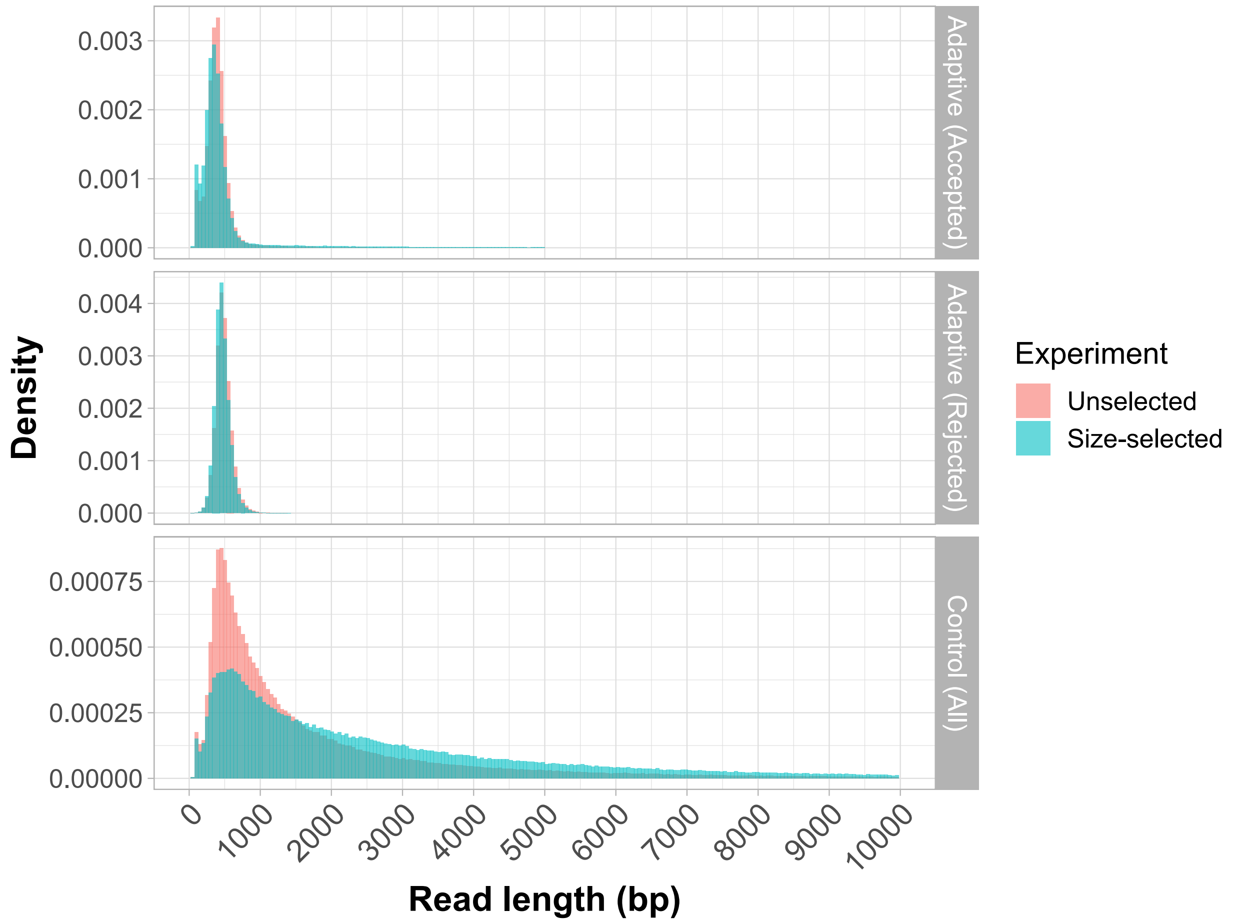

Supplementary Figure 19: Effect of size selection on read length distributions for CBL enrichment. Histograms for accepted (**top**) and rejected reads (**middle**) in adaptive channels, and all reads in control channels (**bottom**).

Read alignment to the Spn23F genome for the Spn23F-only sample revealed that adaptive channels had lower coverage across genome compared to control channels, with the exception of the CBL locus (located at $\sim$ 0.3 Mbp) where coverage reached $\sim 4-5$ fold higher than the rest of the genome (Supplementary Figure 21). Moreover, size selection increased CBL normalised coverage by $\sim$ 1.5 fold over that of the unselected library, whilst coverage of the remainder of the genome was similar between the two libraries. The positive effect of size selection on 23F CBL enrichment was also clear from normalised read coverage across the Spn23F genome, generating a larger spike at the 0.3 Mb position where the CBL is located, relative to the unselected library (Supplementary Figure 21). Therefore, size selection notably increased enrichment when targeting the 23F CBL, in contrast to results from Viehweger *et al.* (2023).

Supplementary Table 6: Read length statistics for CBL enrichment.

Channel type	Read type	Experiment	Mean	Lower Quartile	Median	Upper Quartile
Adaptive	Accepted	Unselected	506	305	386	472
		Size selection	703	271	362	475
	Rejected	Unselected	485	414	475	546
		Size selection	468	400	457	526
Control	All	Unselected	2026	560	1051	2281
		Size selection	3092	874	1963	4045

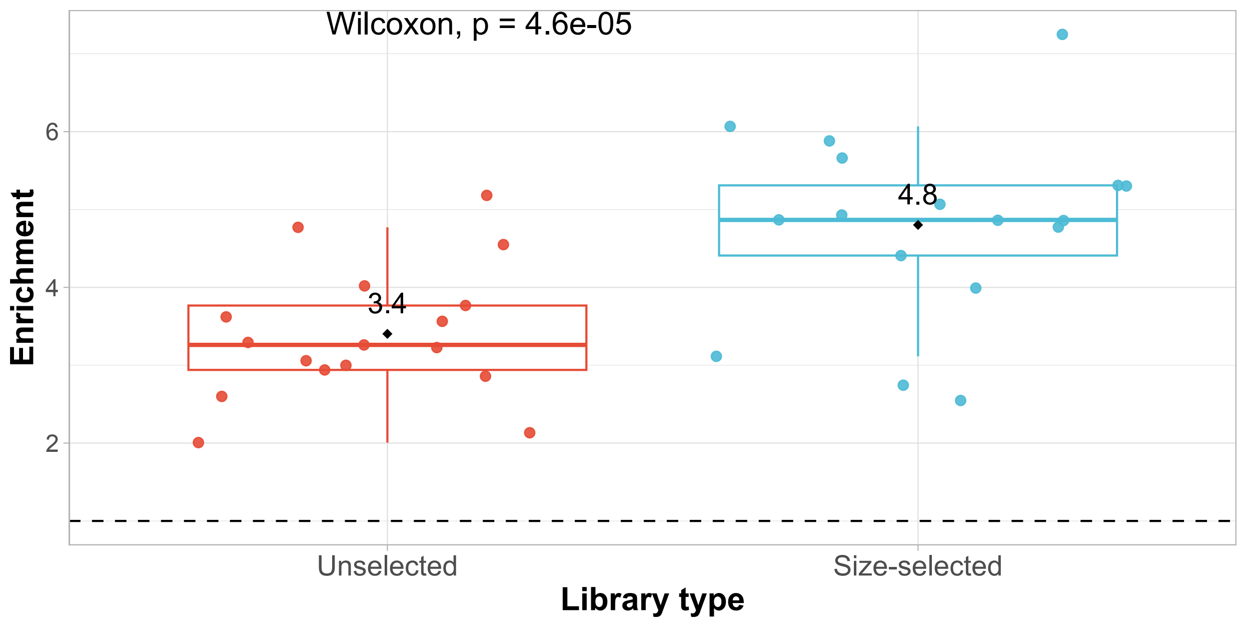

Supplementary Figure 20: Enrichment comparison of pneumococcal CBL with and without size selection, which removed DNA fragments < 10 kb in length. Each data point represents the enrichment of a single CBL found within each library. In libraries containing one strain, only one CBL is present, in 50:50 mixtures, one or more CBL are present. Dashed line describes enrichment = 1 i.e. no enrichment has occurred. Black points highlight distributions means. Distributions were compared using a paired Wilcoxon test.

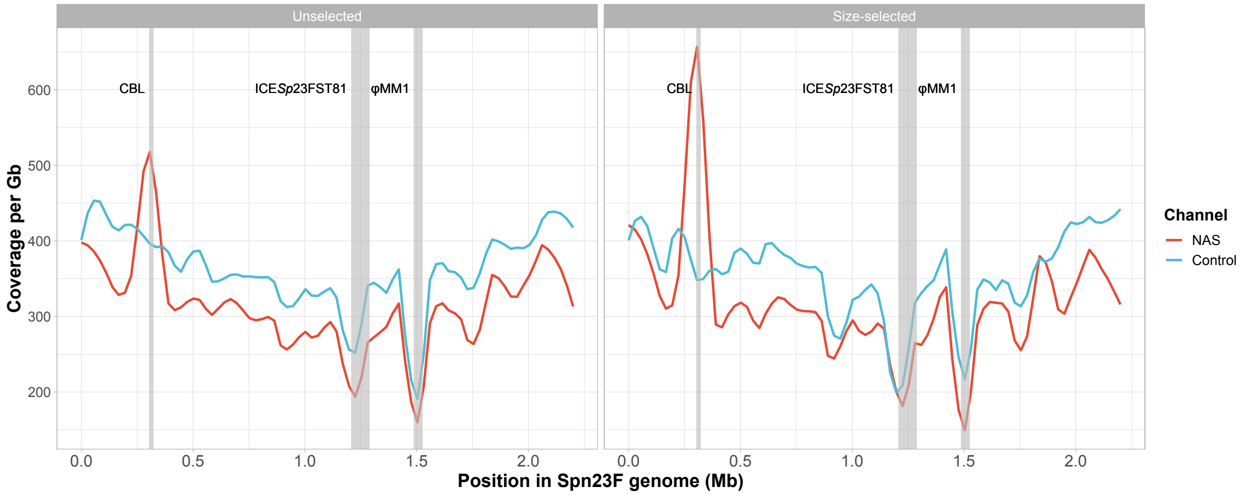

Supplementary Figure 21: Normalised coverage across Spn23F genome for enrichment of 23F CBL for the Spn23F-only sample. Coverage was normalised by dividing the number of read bases aligned at each position in the Spn23F genome by the total number of bases generated for a sample by adaptive or control channels in gigabases (Gb). Columns describe library type; unselected or size-selected. Loci of interest are annotated by grey bars; CBL, as well as ICE*Sp*23FST81 and ψMM1 prophage which are missing in this isolate of Spn23F (Croucher *et al.* 2012).

C.3 Implementing graph pseudoalignment in NAS to capture previously unobserved structural variants.

As NAS effectiveness is impacted by how closely related reads are to the reference sequences (Martin *et al.* 2022; Viehweger *et al.* 2023), using references that are not closely related to the query can result in reference bias, whereby reads will either map in the wrong location, or not map at all (Garrison *et al.* 2018; Eizenga *et al.* 2020), ultimately reducing enrichment. Reference bias can be overcome by including more sequences within the database, capturing a greater amount of diversity and increasing the chance of a match between the read and a reference sequence. However, alignment is still linearly restricted, meaning structural variation, such as reshuffling of genes seen in CBL (Bentley *et al.* 2006), will not be captured and therefore previously unobserved structural variants cannot be enriched for (Supplementary Figure 22a).

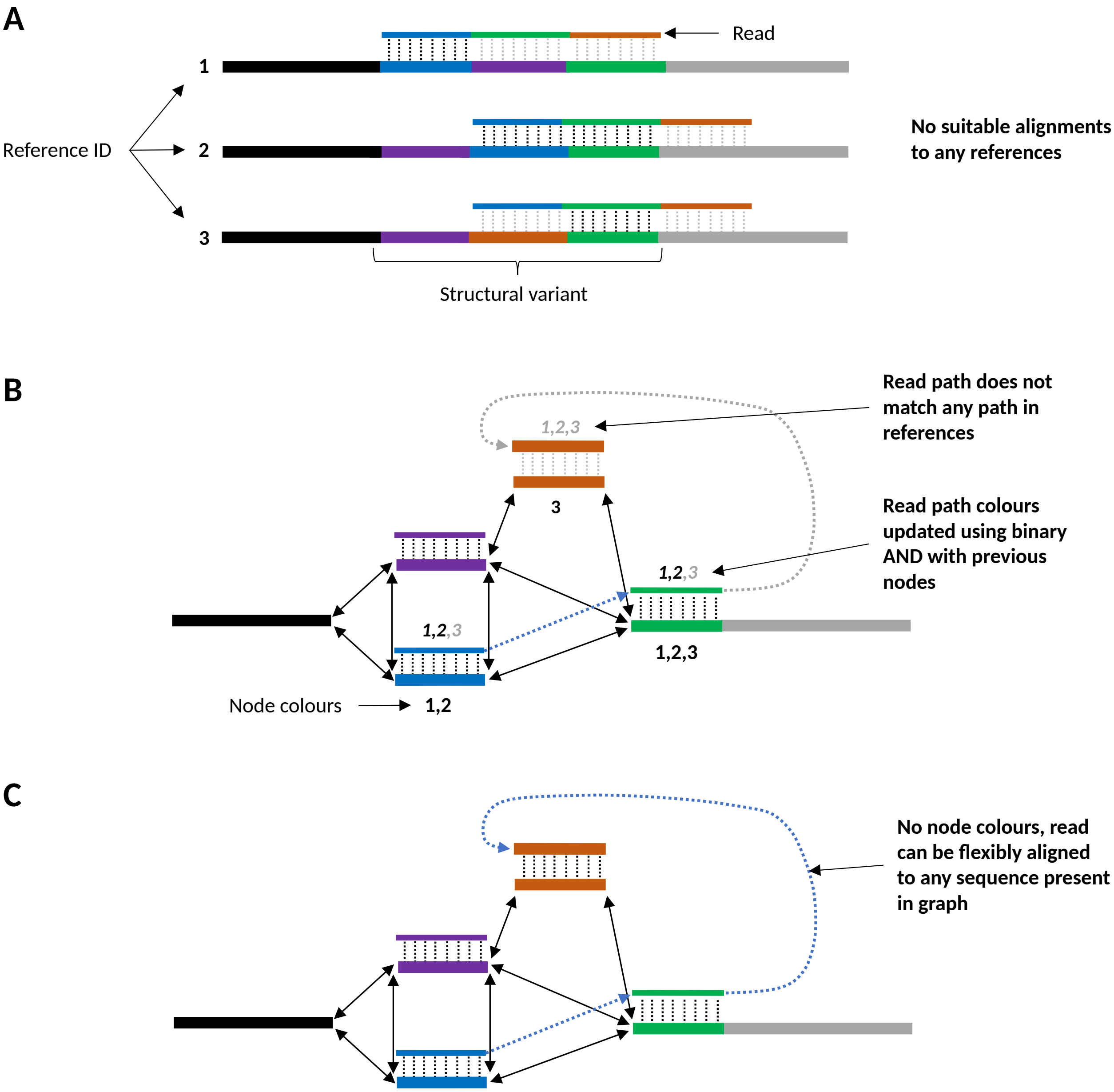

Supplementary Figure 22: Example of alignment of a read containing a previously unobserved structural variant. (**a**) When aligning to a collection of linear reference sequences, the read does not align, despite the references containing the correct sequence blocks. (**b**) Using alignment with a coloured de Bruijn graph, the read is able to align more flexibly, however, as no colours contain all the same sequence blocks as the read, it cannot align fully. (**c**) Using alignment with an uncoloured de Bruijn graph, there are no restrictions on the path the read can take to align to the graph, therefore the read is able to align fully.

Alignment to pangenome graphs has been shown to improve recall of structural variants which are not present in reference sequences (Hickey *et al.* 2020; Sibbesen *et al.* 2023; Dilthey *et al.* 2015). Therefore, graph-based alignment can be integrated into NAS to aid in enrichment of new haplotypes. To achieve this, we developed a read-to-graph *k*-mer-matching method, referred to as graph pseudoalignment, using Bifrost (Holley and Melsted 2020) due to its scalability, fast *k*-mer matching functionality and intuitive C++ API. Pseudoalignment between reads and coloured DBGs has been used previously in transcriptomic and metagenomic studies to identify the likely source of reads (Bray *et al.* 2016; Mäklin *et al.* 2021). However, pseudoalignment to coloured DBGs is still restrictive, for example, if a read contains a set of sequences that are not found collectively in any single reference (Supplementary Figure 22b). Therefore, we implemented a method using pseudoalignment in uncoloured DBGs called GNASTy, which provides the greatest flexibility for identifying novel structural variants (Figure 22c).

As pseudoalignment operates in nucleotide-space, this method requires reads to be base-called from raw current signals generated by the sequencer before pseudoalignment. Therefore, we modified Readfish (Payne *et al.* 2021) to use graph pseudoalignment. Readfish takes raw current signals from a sequencer in 'chunks' (defined as a set period to allow sufficient time for reads to pass through a pore, default = 0.4 seconds), passes them to a base-caller, and then uses minimap2 (Li 2018) to align the reads to linear references. Readfish then sends an accept or reject signal back to the sequencer for each read depending on the result of alignment. We replaced minimap2 with the Bifrost-based DBG pseudoaligner in GNASTy, meaning Readfish can be run in either linear-alignment or graph pseudoalignment modes.

An overview of the graph pseudoalignment in GNASTy is shown in Figure 6. Before a sequencing run, an uncoloured DBG is built using Bifrost, with a user-specified value of k (default = 19 bp). At the start of a sequencing run, Readfish is initialised in graph-alignment mode. As each fragment is sequenced, Readfish passes the raw current to a base-caller, generating a nucleotide sequence (**Step 1**), which is then passed to the graph-pseudoaligner. The read must be above a length cutoff (default = 50 bp) to be aligned, otherwise a 'proceed' signal is sent to allow the read to be sequenced for another chunk. This step is designed to ensure that reads are sufficiently long to avoid false positive or negative mappings. If above the length cutoff, the read is split into its constituent *k*-mers (**Step 2**), with each *k*-mer being queried in the graph (**Step 3**). Once all *k*-mers in the read have been queried, a measure of similarity between the read and graph is calculated. This is either the proportion of matching *k*-mers, P (Equation 2), or a Jaccard index, J (Equation 3), based on the number of matching *k*-mers, N_{match}, and mismatching *k*-mers, N_{mismatch}, between the read and the hypothetical path in the graph. The Jaccard index assumes that for each mismatching *k*-mer in the read, there is also a respective mismatching *k*-mer in the path, meaning that for each mismatch the denominator increases by 2. The Jaccard index can be used to calculate a mash-like distance, D, as used in Mash and SKA (Ondov *et al.* 2016; Harris 2018) (Equation 4). Alternatively, taking $1 - D$ gives the mash-like index, S (Equation 5), an estimate of the sequence identity between the read and the graph, assuming a Poisson distribution of mutations within *k*-mers (Fan *et al.* 2015). Equation 4 can be simplified to Equation 5 to give S in terms of P.

$$P = \frac{N_{\text{match}}}{N_{\text{match}} + N_{\text{mismatch}}} \tag{2}$$

$$J = \frac{N_{\text{match}}}{N_{\text{match}} + 2(N_{\text{mismatch}})} \tag{3}$$

$$D = \begin{cases} 1 & J = 0 \\ \frac{-1}{k} \cdot ln\left(\frac{2J}{(1+J)}\right) & 0 < J \leq 1 \end{cases} \tag{4}$$

$$S = \begin{cases} 0 & P = 0 \\ 1 + \frac{1}{k} \cdot ln(P) & 0 < P \leq 1 \end{cases} \tag{5}$$

S is a more intuitive measure of similarity than P, which is difficult to interpret due to sensitivity to both k and identity between two sequences (Fan *et al.* 2015). We use S in simulations and empirical experiments below. If the similarity between the read and graph is greater than the identity cutoff, a 'stop receiving' signal is sent to allow the rest of the read to be sequenced, otherwise an 'unblock' signal is sent to reject the read (**Step 4**).

C.4 Evaluating mash-like index, *S*, as a similarity measure for graph pseudoalignment.

We evaluated use of the mash-like index, S (Equation 5), which provides an estimate of the real identity between a read and a corresponding path in the DBG. Using simulations, we investigated the effect of varying an S-cutoff on accuracy of target and nontarget assignment at varying k and mutation rate, μ. Reads were generated from a *k*-mer database generated from 100 sequences of 20 kb with 39.49% GC content, matching *S. pneumoniae* (Croucher *et al.* 2009), and mimicking the database used for pneumococcal CBL enrichment (See Methods). 100 reads were generated from the reference database and randomly mutated at different rates before being decomposed into respective *k*-mers with varying size. The *k*-mers were then matched back the reference database to determine the effect of mutation rate, μ, and *k*-mer size on the sensitivity of pseudoalignment. Read length for all simulations was set to 200 bp, which approximately corresponds to a single chunk of sequence generated by Readfish (Payne *et al.* 2021).

Recall of target reads at varying cutoffs of S is shown in Supplementary Figure 23. At $\mu \leq 0.01$, recall was 100% at all values of k, with the exception of high cutoffs of 98% or greater. At higher μ, k size had a large effect on recall of target reads, with large drops between cutoffs of $0 < S \leq 0.02$, followed by 'plateaus' where no further reads were rejected, and finally by a sharp decline at cutoffs of $0.6 \leq S \leq 1.0$. However, the S cutoff at which the second decline in target read assignment occurred was dependent on k, but not μ. These distributions can be explained by the proportion of matching *k*-mers in each scenario. Higher μ values cause a greater number of reads to have a no matching *k*-mers ($S = 0$), which is more likely at larger values of k, resulting in the initial sharp decline at $0 < S \leq 0.02$. The plateau corresponds to the lower bound of S sensitivity at a given μ and k, when only a single *k*-mer matches between the read and the reference. At $0.6 \leq S \leq 1.0$, the cutoff is sensitive to reads that have more than one matching *k*-mer. This sensitive range varies with k but not μ: for $k = 19$ the sensitive range begins at cutoff=0.75 for $\mu = 0.1$ and $\mu = 0.16$. In comparison, for $k = 31$ the sensitive range begins at a S cutoff of 0.85 for $\mu = 0.1$ and $\mu = 0.16$. Therefore, for read classification during pseudoalignment to be sensitive to sequence identity, choice of S must sit within the μ sensitive range for a given value of k.

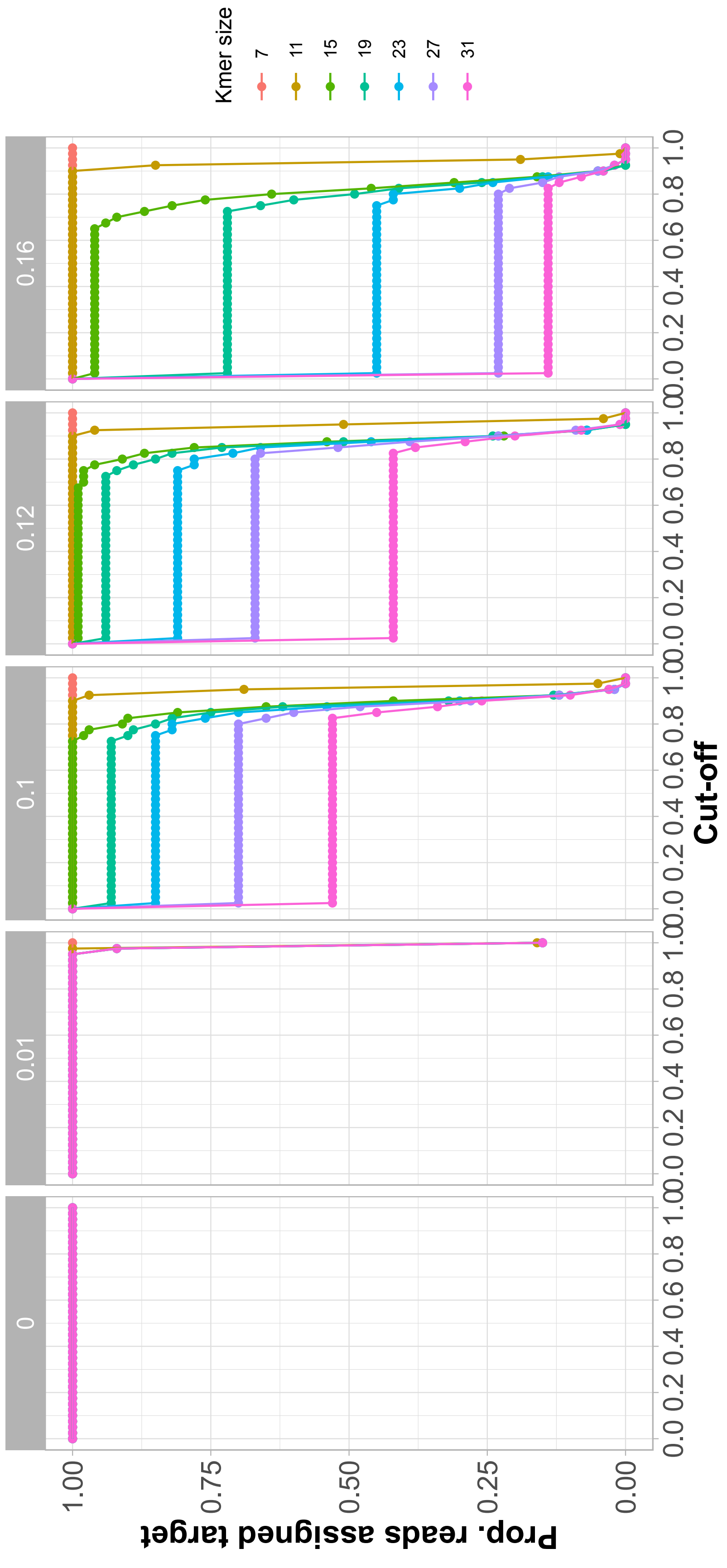

Supplementary Figure 23: Effect of cutoff of mash-like similarity, S, and μ on recall of target reads. Plots show the proportion of reads identified as a target based on a set minimum cutoff of S between the read and reference database. Columns indicate the mutation rate in the reads per base, μ. Read length set at 200 bp.

We then investigated the concordance of S with sequence identity when varying k (Supplementary Figure 24) using LOESS regression, a form of linear regression with fits to data locally, resulting in a smooth regression line. Results highlighted that the agreement between S and pairwise identity is dependent on k. When $k < 15$, sequence identity is always overestimated due to random k-mer matching. For $k \geq 15$, when the read and reference match well (identity ≥ 0.9), S estimates pairwise identity correctly. At ≤ 0.9 identity, S underestimates the pairwise identity, with a larger underestimation at higher k due to increased k-mer mismatching.

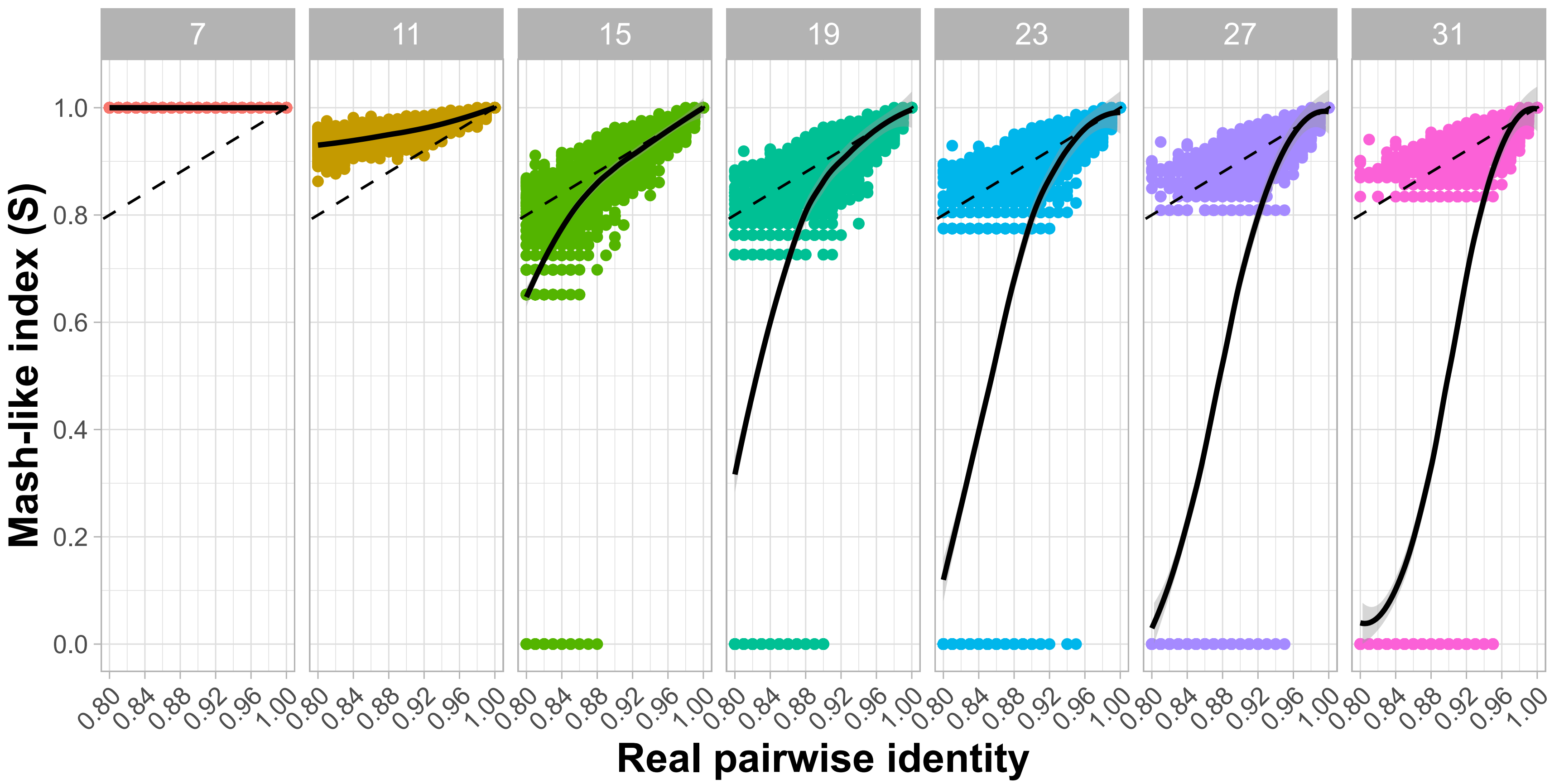

Supplementary Figure 24: Concordance of mash-like distance, S, and identity between reads and reference sequence with varying k size. Each point describes a single read with a given value of μ. Columns describe k size. Dashed line represents identity line, y=x. Solid line represents LOESS regression between S and real identity, with grey shading for 95% confidence intervals.

We then calculated the root mean square error (RMSE) between S and the identity line, y=x, which is the ideal relationship between S and real pairwise identity, for real pairwise identity in range $0.9 - 1.0$ at varying read lengths (Supplementary Figure 25). Results show that RMSE is reduced as read-length increases at all $k \geq 15$, due to the increased probability of k-mer matching in longer reads, and is lowest at $k = 15$. At $k < 15$, random k-mer matching means read-length has no impact on RMSE. Overall, agreement between S and the real pairwise identity of a read and reference sequence is dependent on k, read length and the underlying rate of mutation, μ. When μ is too large, S will tend to underestimate the real pairwise identity at $k \geq 15$, which can be remedied by increasing alignment length.

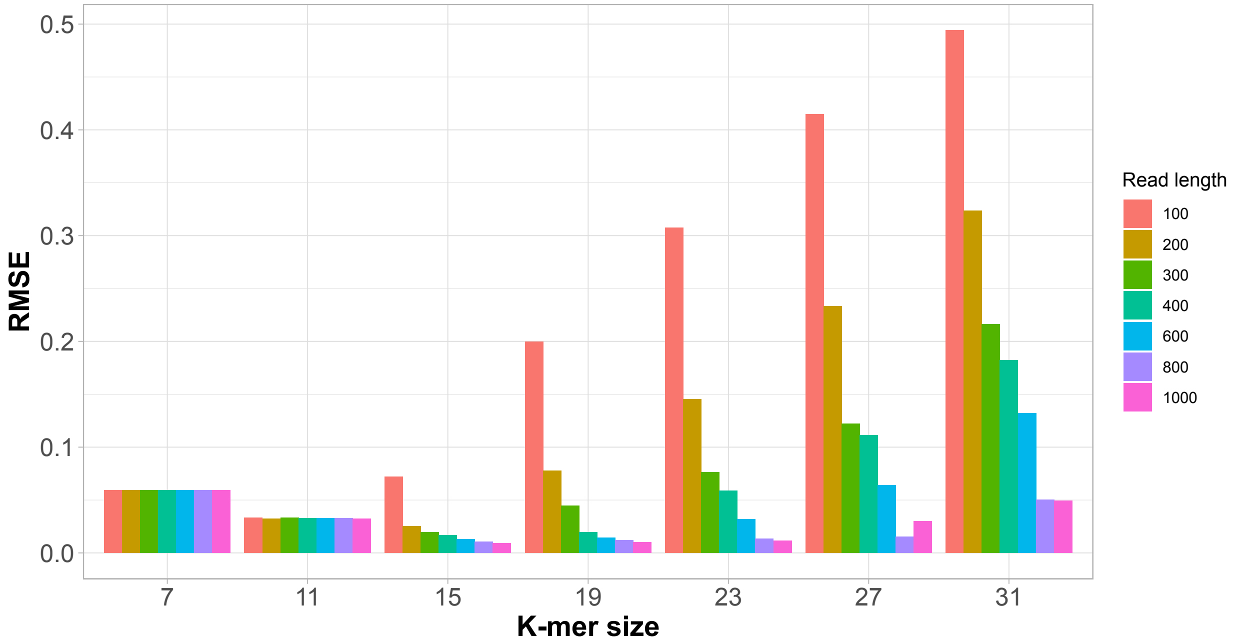

Supplementary Figure 25: RMSE between identity line, y=x, and pairwise identity vs. S relationship for pairwise identity in range $0.9 - 1.0$ for varying k size and read length.

We then considered the effect of the S cutoff and k on the specificity of pseudoalignment. 100 nontarget reads were randomly generated from a different 2.2 Mbp sequence with the same GC content as before, and matched to the same reference k-mer database as used above. These reads represent nontarget sequences, and so ideally should not match the original reference with high identity. At $k > 15$, almost no reads had $S > 0$, indicating that no k-mers matched between the nontarget reads and the graph (Supplementary Figure 26). Contrastingly, for $k \leq 15$ there were a large proportion of nontarget reads that matched with $S \geq 0.5$, indicating lower specificity of graph pseudoalignment with lower values of k.

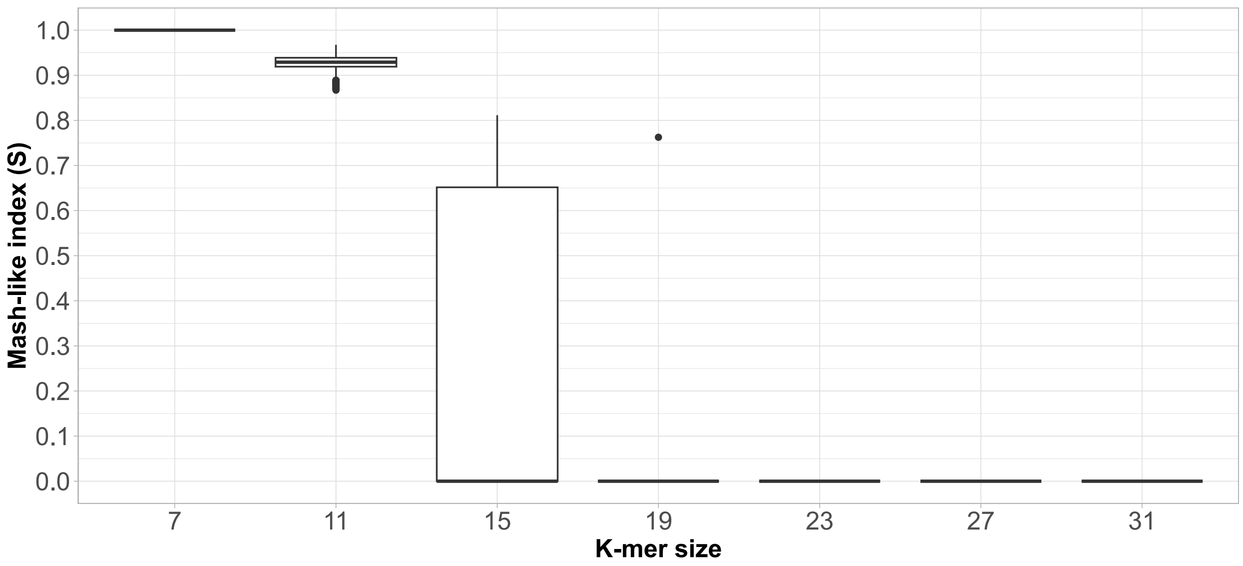

Supplementary Figure 26: Effect of k-mer size on mash-like index, S, between nontarget reads and reference. Boxplots show the proportion of random matching k-mers per read. Read length set at 200 bp.

To directly compare sensitivity and specificity of varying S cutoffs, we generated receiver operating characteristic (ROC) curves and conducted area-under-curve (AUC) analysis (Supplementary Figures 27 and 28). These visualisations compare the sensitivity and specificity of a parameter set as a cutoff is increased. An ideally performing parameter set should follow the top left corner of the ROC curve plot with an AUC of 1, indicating a high true positive rate and low false positive rate. A poorly performing parameter set will follow the line $y = x$ and have an AUC of 0, which is equivalent to a random assignment of target or nontarget. At $\mu \leq 0.01$, all parameter sets with $k \geq 7$ had the ideal relationship between true positives and false positives. As μ increased, $k = 15$ had the best performance, with $k < 15$ having reduced specificity, and $k > 15$ having reduced sensitivity.

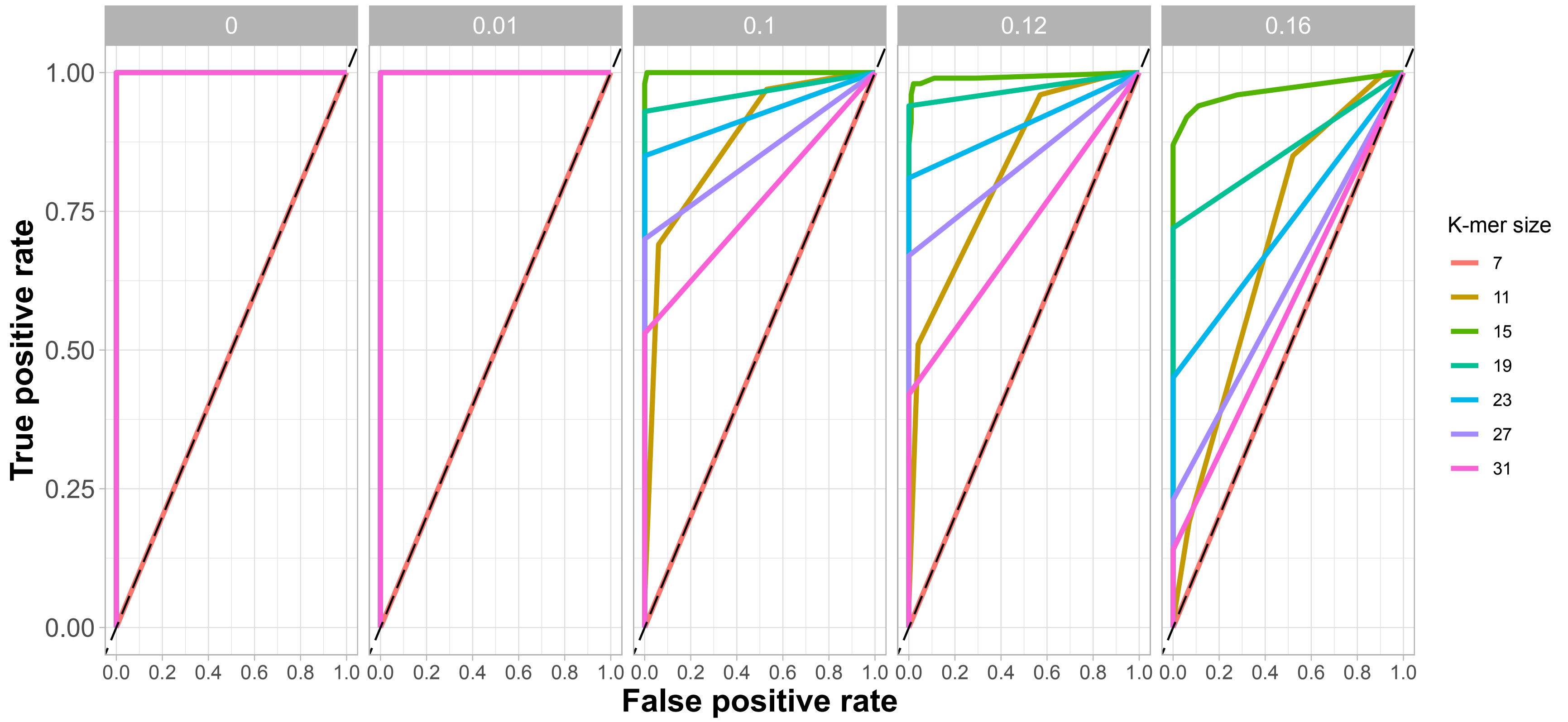

Supplementary Figure 27: ROC curves for varying S cutoff and μ. Columns indicate the mutation rate in the reads per base, μ. Dashed lines represent line $y = x$. Read length set at 200 bp.

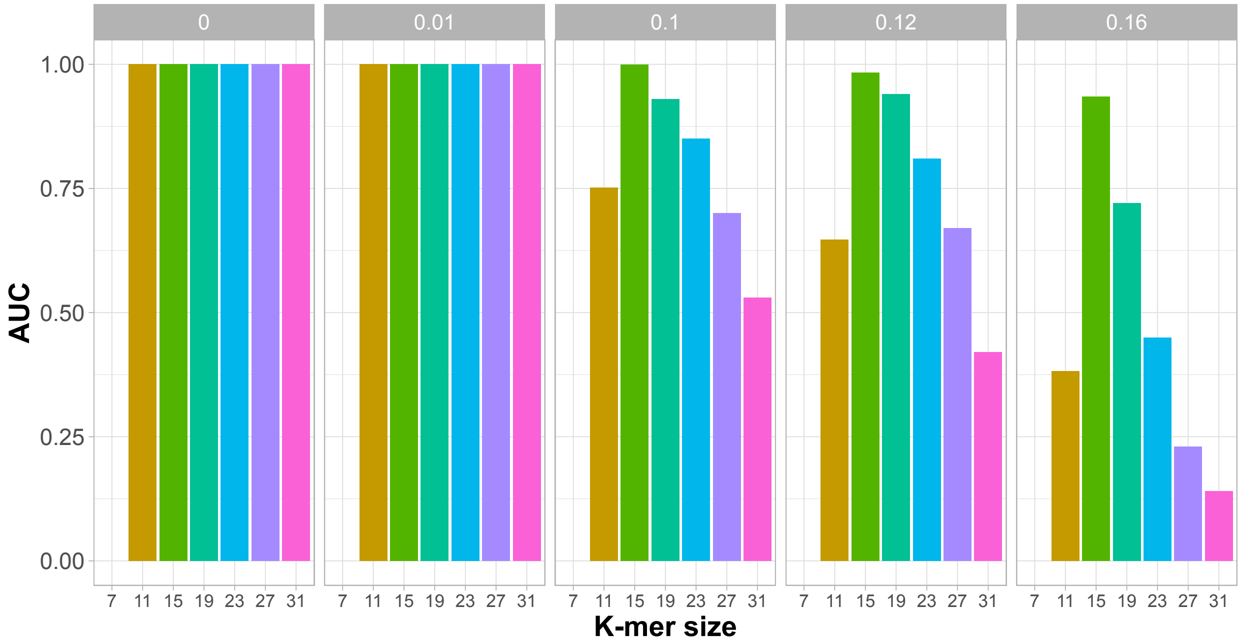

Supplementary Figure 28: AUC from ROC curve for varying k-mer size and μ based on mash-index, S. Columns indicate the mutation rate in the reads per base, μ. Read length set at 200 bp.

Overall, concordance of S with μ was shown to be dependent on k, with $k \sim 15$ shown to have a good trade off of sensitivity and specificity even at $\mu = 0.16$. However, measures of sensitivity and specificity were limited in these simulations, as nontarget reads in real metagenomes will likely come from a greater diversity of genomes than the single 2.2 Mbp genome simulated here. Therefore, graph pseudoalignment parameters required further simulation-based optimisation to determine the best combination to use empirically.

C.5 Evaluating Graph Pseudoalignment using simulated sequencing runs.

The simulations in the Section C.4 are useful for understanding how pseuodalignment parameters impact sensitivity and specificity, and guided down-selection of k to ~ 15. However, there was no clear choice of k and S cutoff for optimal performance. Therefore, before testing graph pseudoalignment empirically, we ran more realistic simulations where the process of sequencing and read rejection were simulated, whilst pseudoalignment parameters were varied for down-selection.

For simulations, we used a 'recording' of the Spn23F whole genome enrichment sequencing experiment using a size-selected library containing a 50-50 mixture of Spn23F and *E. coli* (see Results, Section 'NAS performance depends on microbiome composition'). This recording generated simulated target and nontarget reads, which were then aligned using graph pseudoalignment or minimap2 to the database containing 106 CBL sequences (See Methods), enriching for the 23F CBL. These simulations result in rejected reads being 'chopped' into smaller reads, rather than being rejected, as would occur in a normal sequencing run (Munro *et al.* 2024). Therefore, in order to compare the effect of graph pseudoalignment parameters, we analysed the read lengths of four read categories based on the rejection decision and post-sequencing alignment to the 23F CBL: true positives (TPs), reads that were correctly accepted; false positives (FPs), reads that were incorrectly accepted; true negatives (TNs), reads that were correctly rejected; and false negatives (FNs), reads that were incorrectly rejected. Ideally, TPs should be long relative to the other categories, as these reads have been accepted and therefore sequenced fully. FPs and TNs should be as short as possible; FPs should ideally be reads too short to have been rejected, whilst short TNs indicate fast rejection time. FNs should not be present, as they result from acceptance parameters that are too conservative.

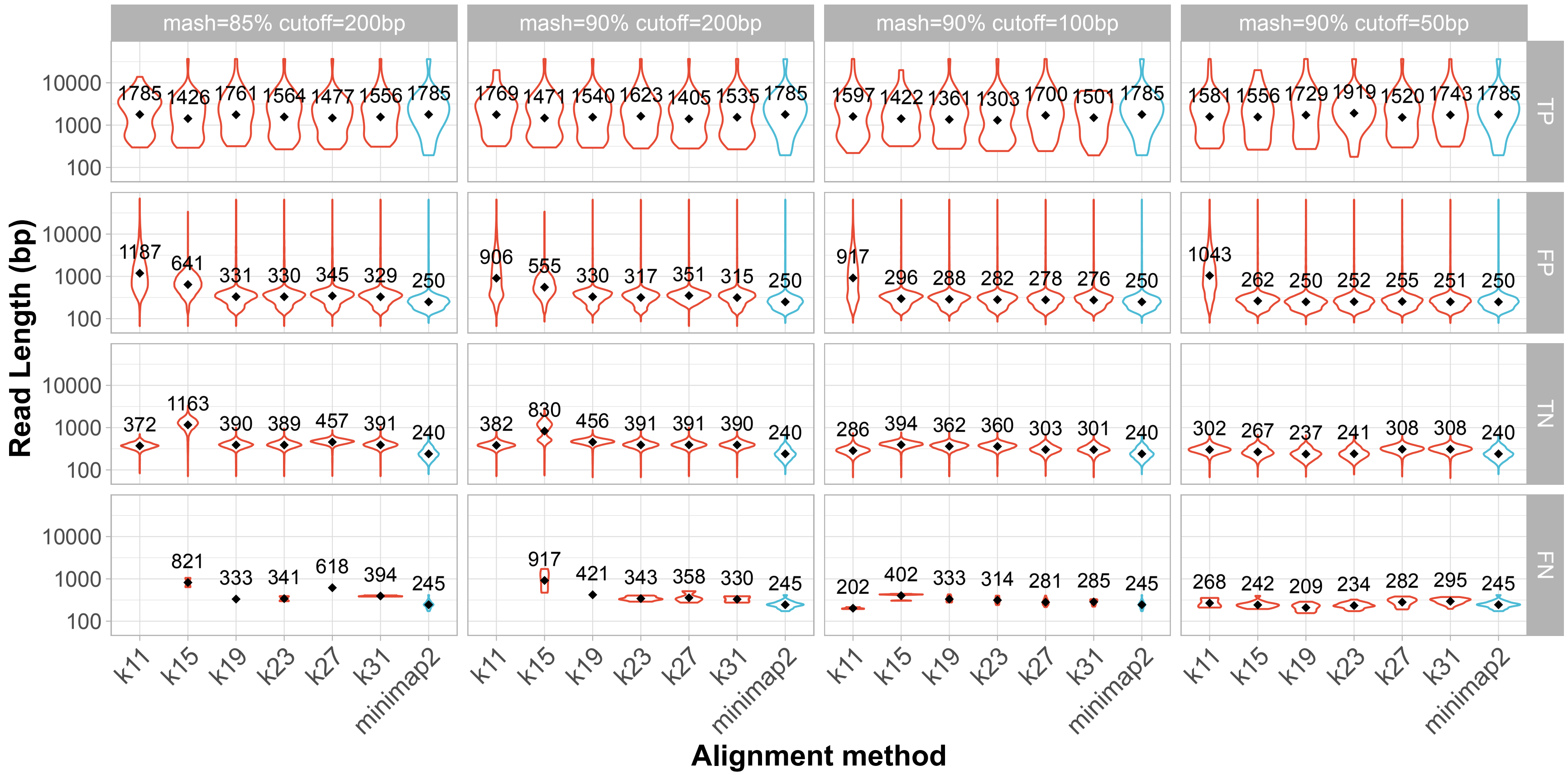

Supplementary Figure 29: Simulation read length comparison of graph pseudoalignment parameters and minimap2. X-axis describes either the k size used in graph pseudoalignment or minimap2 alignment. Columns describe pseudoalignment parameters (mash: minimum S, cutoff: minimum read length). Rows describe categories of reads based on accept/reject decision and post-alignment to 23F CBL; TPs, reads that were correctly accepted, FPs, reads that were incorrectly accepted, TNs, reads that were correctly rejected, and FNs, reads that were incorrectly rejected.

We initially tested $S = 85\%$, as simulations in the previous section highlighted this was within the sensitive range of all k values (Supplementary Figure 23), as well as a minimum length cutoff of 200 bp (approximate size of one chunk from Payne *et al.* (2021)), which sets the minimum length of a read that can be aligned. An appropriate minimum length cutoff ensures that reads are not rejected prematurely if the initial sequence generated in the first chunk aligns poorly. TP read lengths were similar across all k values, and comparable to minimap2 (Supplementary Figure 29, left). However, FP and TN reads were longer on average than minimap2 for all values of k, indicating either slower rejection speed or lower specificity. Only $k = 11$ resulted in no FN reads due to low specificity but high sensitivity, resulting in no reads being rejected incorrectly.

As longer FP reads indicated graph pseudoalignment may have lower specificity than minimap2, we increased S to 90% (Supplementary Figure 29, center-left). This change reduced the average size of FP reads without affecting TPs for all values of k, however, TNs were still longer than minimap2. We therefore decreased the minimum length cutoff to 100 bp and 50 bp with the aim of increasing rejection speed (Supplementary Figure 29, center-right and right respectively). Reducing this parameter to 50 bp had no impact on TP read lengths, but reduced the FP and TN lengths to similar ranges observed with minimap2.

To determine the best pseudoalignment parameter set, we compared the proportion of bases assigned to TP and TN categories, rather than read lengths. We ignored FNs and FPs, as these are equivalent to $1-$TP and $1-$TN respectively. Graph pseudoalignment performed similarly to, or outperformed, minimap2 at $k > 15$ (Supplementary Figure 30). Proportions of TP bases were always higher for graph pseudoalignment than minimap2, but were reduced as parameters became more stringent (increasing S, decreasing length cutoff). Proportions of TN bases were also the same or greater for $k > 15$ for all parameters, and did not change substantially at $k \geq 19$.

Additionally, we compared parameters based on their F_1 score based on base proportions:

$$F_1 = \frac{2\text{TP}}{2\text{TP} + \text{FP} + \text{FN}} \tag{6}$$

which describes the trade off between sensitivity and specificity, with a value of 1 indicating recall of all TPs with no FPs. Based on F_1, the most stringent parameter set ($k = 31$, $S = 90\%$, minimum length cutoff 50 bp) performed the best, also outperforming minimap2 (Supplementary Figure 31).

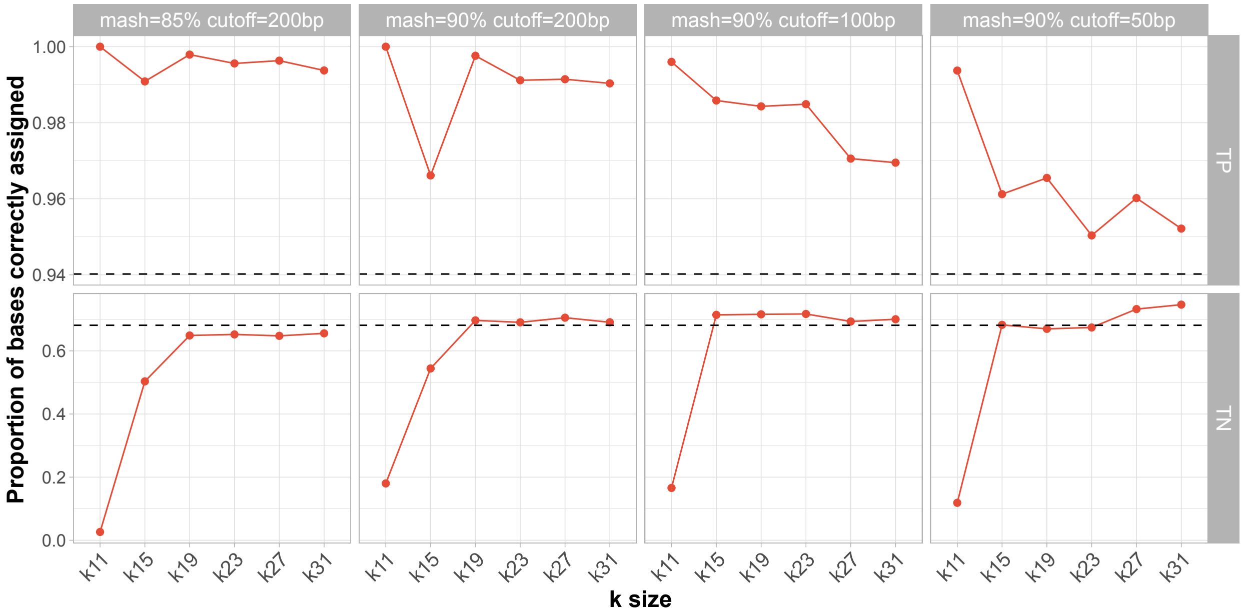

Supplementary Figure 30: Comparison of graph pseudoalignment parameters and minimap2 based on proportions of bases correctly assigned to positive and negative categories. Minimap2 results are indicated by the black dashed line on each facet. Columns describe pseudoalignment parameters (mash: minimum S, cutoff: minimum read length). Rows describe categories of reads based on accept/reject decision and post-alignment to 23F CBL: TPs, reads that were correctly accepted; TNs, reads that were correctly rejected.

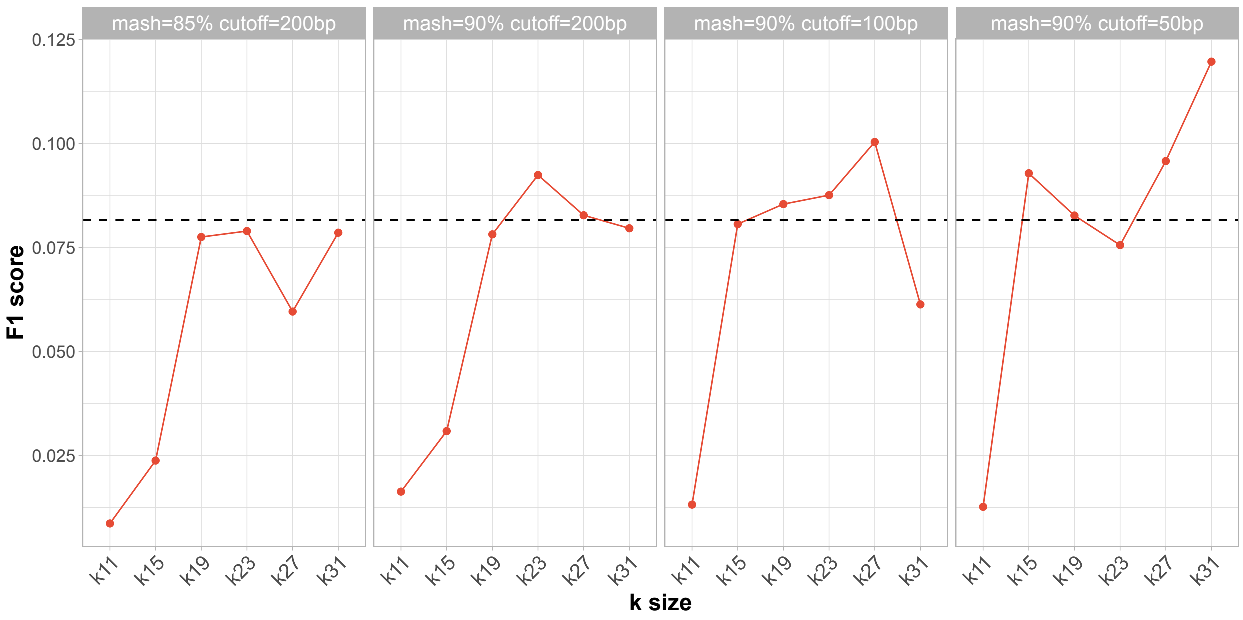

Supplementary Figure 31: Comparison of F_1 scores of graph pseudoalignment parameters and minimap2 based on base proportions. X-axis describes either the k size used in graph pseudoalignment. Minimap2 results are indicated by the black dashed line on each facet. Columns describe pseudoalignment parameters (mash: minimum S, cutoff: minimum read length).

We hypothesised that graph pseudoalignment will outperform minimap2 when enriching for an unobserved sequence due to more flexible alignment (Supplementary Figure 22). To compare the performance of graph pseudoalignment and linear alignment with a reference database where the target sequence is missing, we generated a partial reference database containing divergent sequences from 23F CBL. Using cluster assignments from Mavroidi *et al.* (2007), we included sequences from CBL outside cluster two only, which contains the 23F CBL. As shown in Supplementary Figure 32, the presence of homologous blocks between 23F and CBL in different clusters varies between clusters. For example, the 23A CBL, in the same cluster as 23F, has 12 blocks in common, covering $\sim$ 17.5 kb, whilst 25F, belonging to cluster 3, has only two blocks in common, covering $\sim$ 3.5 kb. By combining CBL sequences from different clusters apart from cluster two, we were able to test the ability of graph pseudoalignment to enrich for haplotypes missing from the reference database.

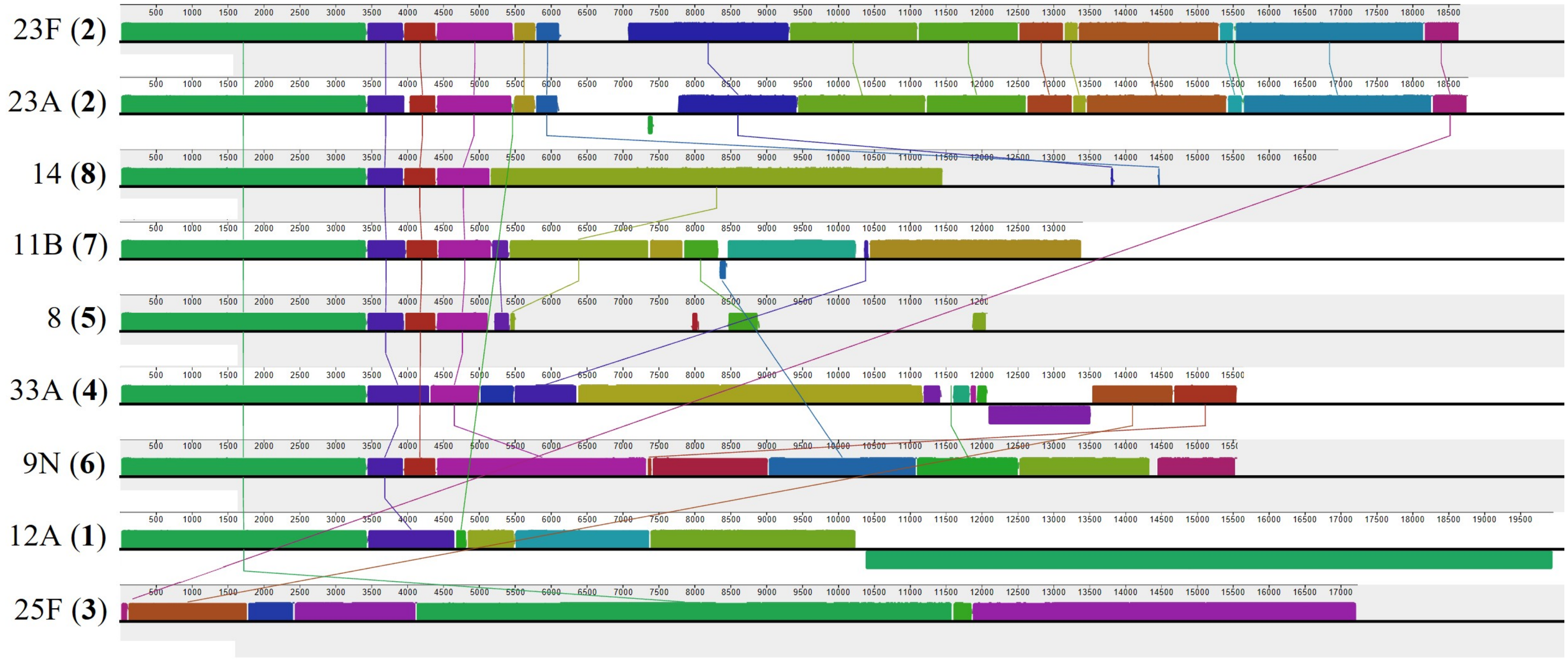

Supplementary Figure 32: Structural alignment of representative pneumococcal CBL. Labels describe the serotype, followed by the cluster assignment from Mavroidi *et al.* (2007) in brackets. Alignments were generated using ProgressiveMauve and visualised by MauveViewer (Darling *et al.* 2010). Same coloured blocks were identified as homologous by ProgressiveMauve between CBL and are connected by coloured lines. CBL coordinates are given for each CBL sequence.

We re-ran simulations using the same run recording used above, aligning to the partial CBL reference database using minimap2 and graph pseudoalignment, aiming to enrich for the 23F CBL. We varied k, keeping S and read-length cutoff at the best performing parameters from previous simulations ($S = 90\%$, read length cutoff 50 bp). Results showed that with a partial database, $k = 19$ performed best of all parameters, with a combination of high TP and TN, leading to the only F_1 score above that of minimap2 (Supplementary Figures 33 and 34). These results indicate that reduced stringency should be used when a target is not present in the reference database, as larger values of k lead to incorrect rejections due to k-mer mismatching.

Overall, we have shown that graph pseudoalignment can theoretically increase the sensitivity of target read identification. The best performing parameters from these simulations did not agree with results from Section C.4, with $k = 19$ and $k = 31$ shown to perform best, rather than $k = 15$. Therefore, alignment identity parameters, k and S, can have a large effect on NAS accuracy, which cannot be entirely modelled using simple simulations.

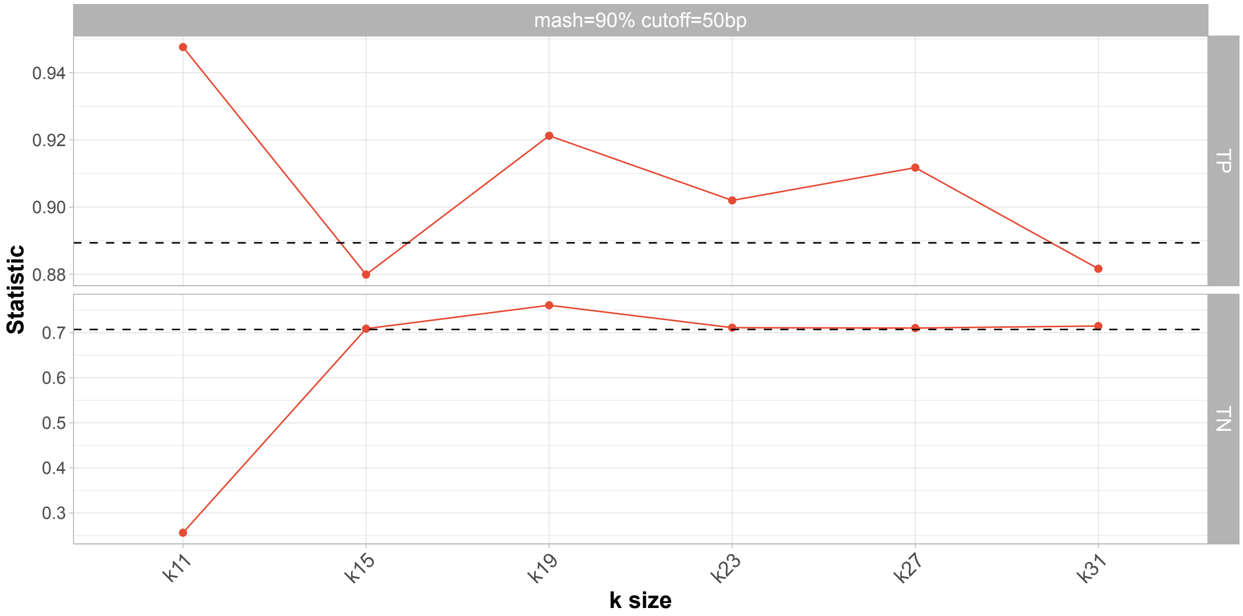

Supplementary Figure 33: Comparison of graph pseudoalignment parameters and minimap2 based on base proportions. X-axis describes either the k size used in graph pseudoalignment. Minimap2 results are indicated by the black dashed line on each facet. Facet heading describes pseudoalignment parameters. Rows describe categories of reads based on accept/reject decision and post-alignment to 23F CBL; TPs, reads that were correctly accepted, TNs, reads that were correctly rejected.

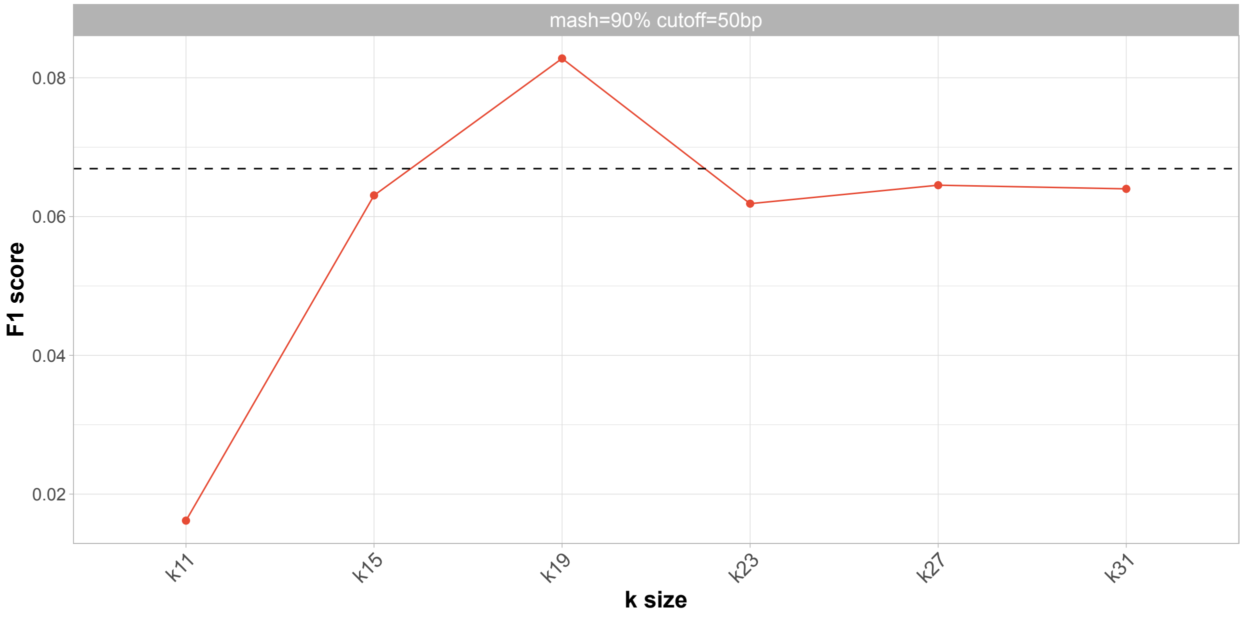

Supplementary Figure 34: Comparison of F_1 scores of graph pseudoalignment parameters and minimap2 using a partial CBL database. X-axis describes either the k size used in graph pseudoalignment. Minimap2 results are indicated by the black dashed line on each facet. Facet heading describes pseudoalignment parameters.

C.6 Graph pseudoalignment performs similarly to linear alignment when enriching for an observed locus

To determine whether graph pseudoalignment in GNASTy could perform equally well using a full CBL database, we enriched for the 23F CBL using a reference database containing all 106 CBL sequences, including 23F. The experimental setup was the same as described in Figure 1a and b, with GNASTy using parameters $k = 19, S = 75\%$.

GNASTy performed similarly to minimap2, with either method performing better at specific target concentrations and nontarget species (Supplementary Figure 35). Both methods significantly increased the absolute yield of 23F bases compared to control channels by 2.8- and 3.4-fold on average for minimap2 and GNASTy respectively (Supplementary Figure 36). Normalised coverage was similar between the two methods (Supplementary Figure 37). Moreover, coverage was more even across the 23F CBL using the full database relative to the partial database (Supplementary Figure 7), particularly when comparing the samples containing CBL proportion at 4×10^{-3}. This finding suggests that the drop in coverage in the central region of the 23F CBL was caused by absence of similar sequences in the reference database. Therefore, fragments originating from this central region were incorrectly rejected, observed when using minimap2 or graph pseudoalignment, although graph pseudoalignment maintained greater coverage across the remainder of the CBL.

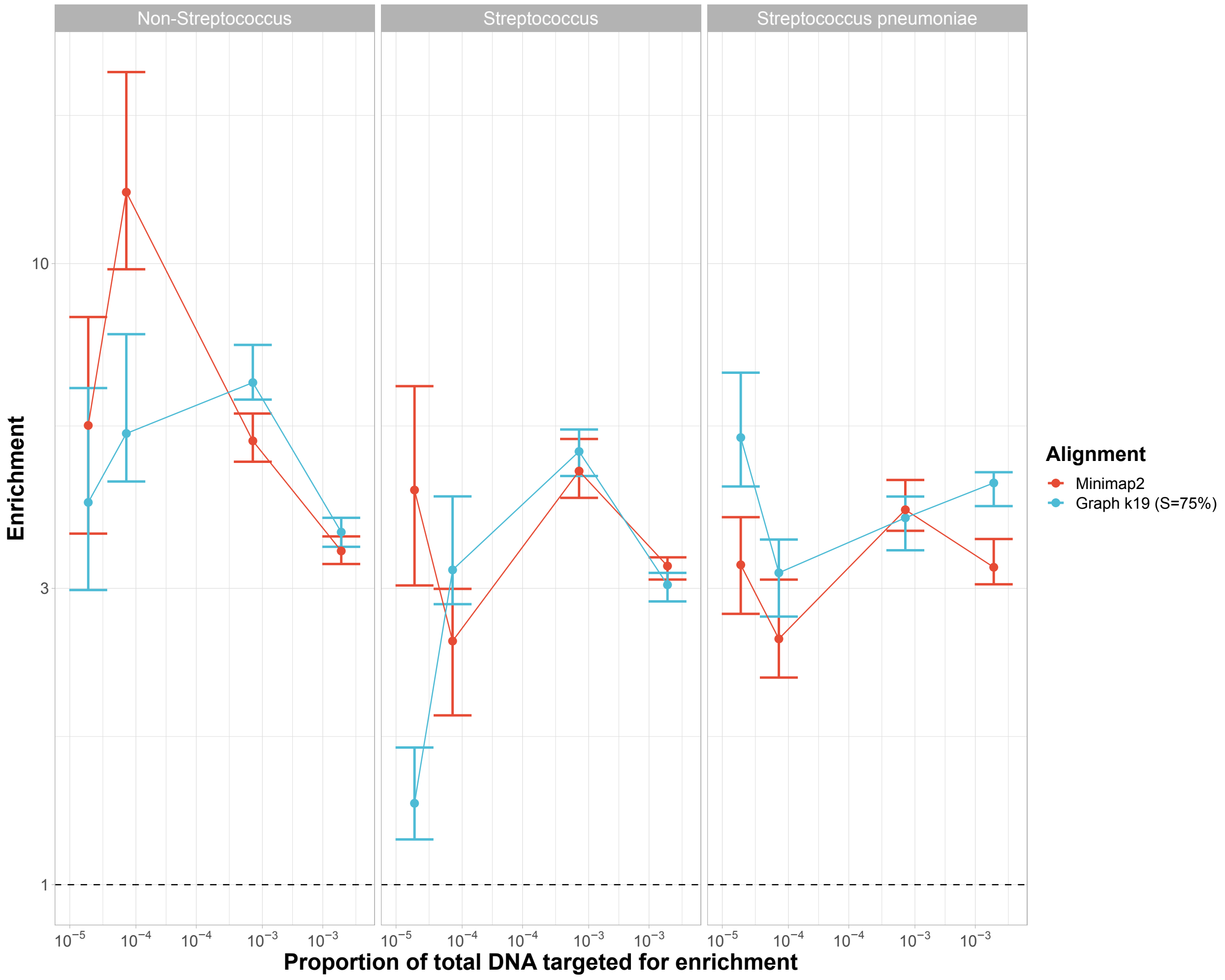

Supplementary Figure 35: Enrichment comparison of 23F CBL at different concentrations of target between minimap2 and graph pseudoalignment in GNASTy when aligning to a full CBL reference database using V14 chemistry. Bar ranges are inter-quartile range of enrichment from 100 bootstrap samples of reads. Data points connected by lines are observed enrichment values for each library, with solid lines connecting the same genome diluted at different concentrations. Columns describe the type of nontarget species (Non-*Streptococcus* = *E. coli*, *Streptococcus* = *S. mitis* and *S. pneumoniae* = *S. pneumoniae* R6). Dashed line describes enrichment = 1 i.e. no enrichment has occurred.

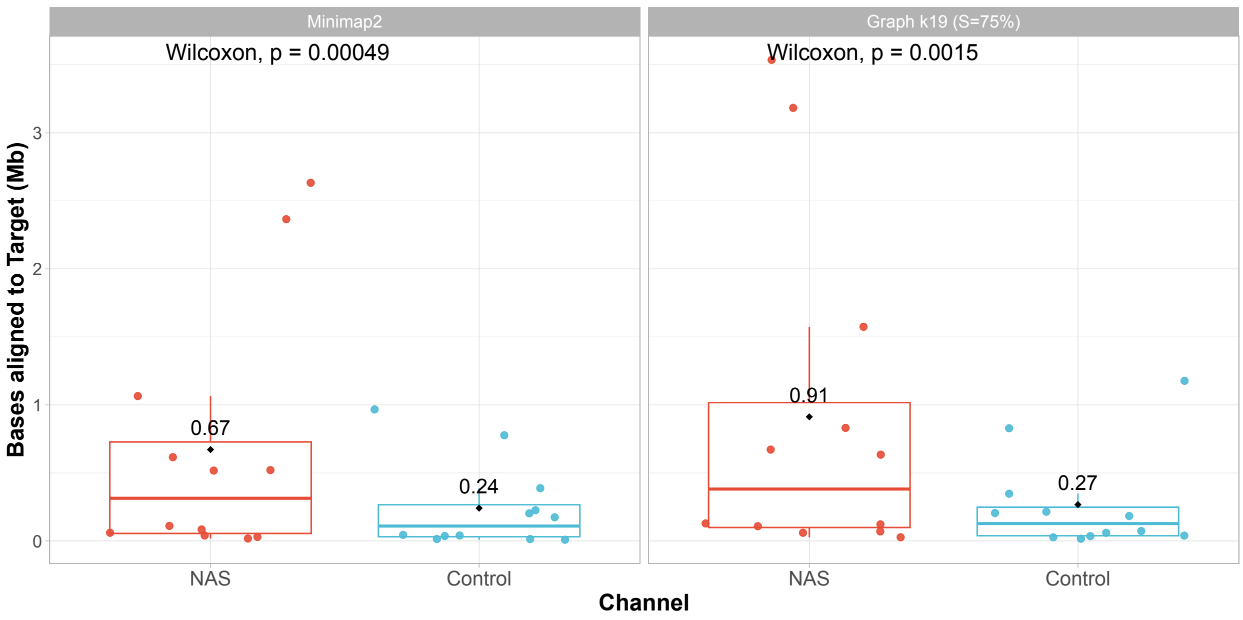

Supplementary Figure 36: Absolute yield (in megabases) of bases aligning to the 23F CBL when aligning to a full CBL database. Each data point represents the enrichment of the 23F CBL found within each library. Distributions from control and NAS channels were compared using a paired Wilcoxon test.

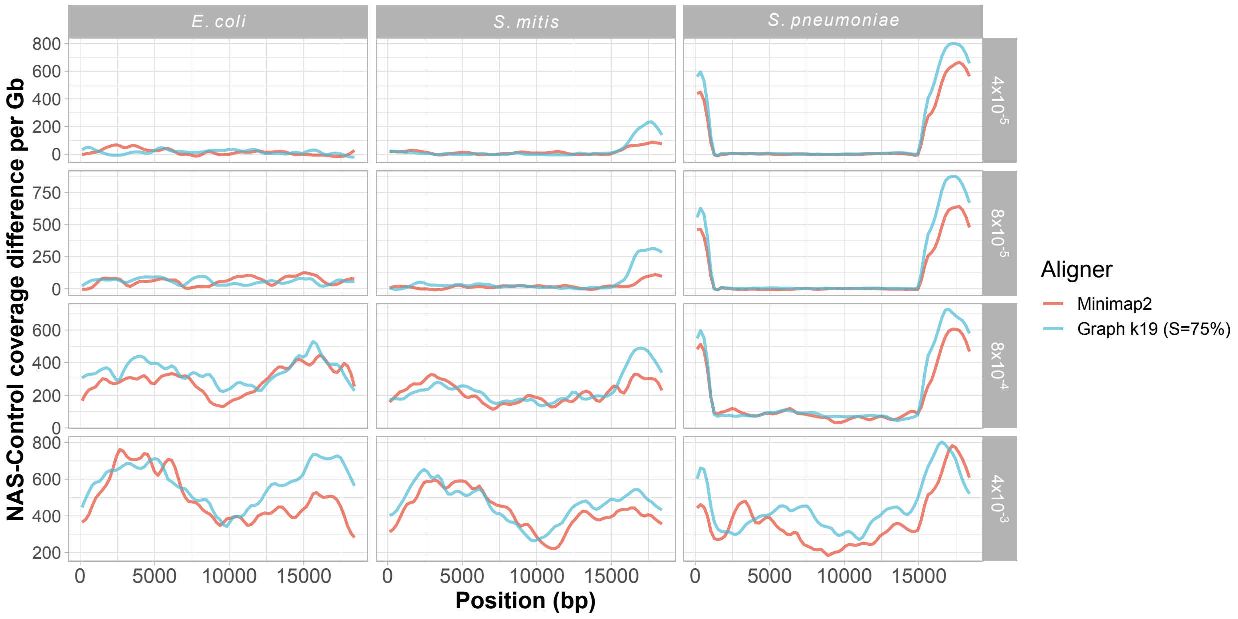

Supplementary Figure 37: Normalised coverage difference between NAS and control channels across 23F CBL using a full CBL reference database. NAS-control coverage difference per gigabase (Gb) calculated by normalising the read coverage for each locus by the amount of data generated (in Gb) for each respective sample and channel, and then negating the normalised coverage for control channels from NAS channels for each locus. Rows describe the proportion of target DNA present in the sample.

Comparison of assembly quality highlighted similar performance between minimap2 and graph pseudoalignment in GNASTy (Supplementary Figure 38). Read coverage was higher for adaptive channels than control channels, although assembly quality was similar between control and NAS channels for both tools. For minimap2, assembly quality was

identical between NAS and control channels. For GNASTy, a gap present in the centre of the assembly from control channels was covered in the NAS channel assembly, as observed when using the partial CBL database for alignment (Supplementary Figure 9). However, the NAS channel assembly was missing a section at the 18 kb end of the CBL, which was captured by the control assembly, despite read coverage being similar or higher from adaptive channels. This effect was also observed in Supplementary Figure 9 and discussed in the Results (see Section ‘Graph-based alignment facilitates the discovery of novel CBL’).

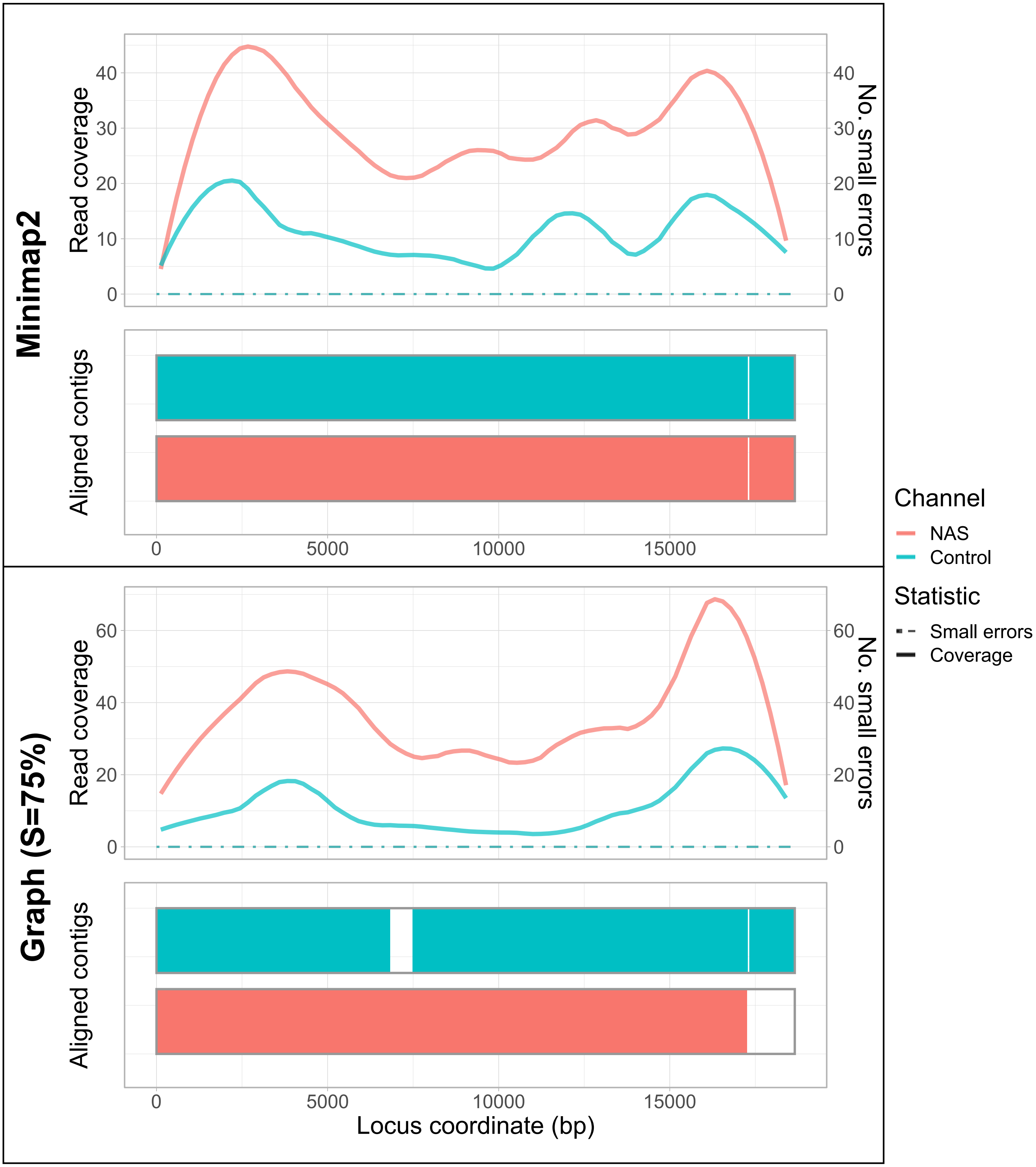

Supplementary Figure 38: Spn23F CBL assembly comparison across alignment methods using a full CBL database during NAS. Each panel describes a Spn23F assembly generated from 0.1 Spn23F dilutions with *S. mitis*. For each panel, the top plot shows the read coverage (solid), defined as the absolute number of bases aligning to a locus, and number of small errors ($\leq$ 50 bp, dashed), whilst the bottom plot shows aligned contigs (colours) and large errors ($>$ 50 bp) in each assembly.

Supplementary References

Bentley SD, Aanensen DM, Mavroidi A, Saunders D, Rabbinowitsch E, Collins M, Donohoe K, Harris D, Murphy L, Quail MA *et al.* 2006. Genetic analysis of the capsular biosynthetic locus from all 90 pneumococcal serotypes. *PLoS Genetics*. **2**: 0262–0269.

Bray NL, Pimentel H, Melsted P and Pachter L. 2016. Near-optimal probabilistic RNA-seq quantification. *Nature biotechnology*. **34**: 525–527.

Chochua S, D'Acremont V, Hanke C, Alfa D, Shak J, Kilowoko M, Kyungu E, Kaiser L, Genton B, Klugman KP *et al.* 2016. Increased Nasopharyngeal Density and Concurrent Carriage of Streptococcus pneumoniae, Haemophilus influenzae, and Moraxella catarrhalis Are Associated with Pneumonia in Febrile Children. *PLOS ONE*. **11**: e0167725.

Croucher NJ, Harris SR, Barquist L, Parkhill J and Bentley SD. 2012. A high-resolution view of genome-wide pneumococcal transformation. *PLoS Pathogens*. **8**:

Croucher NJ, Walker D, Romero P, Lennard N, Paterson GK, Bason NC, Mitchell AM, Quail MA, Andrew PW, Parkhill J *et al.* 2009. Role of Conjugative Elements in the Evolution of the Multidrug-Resistant Pandemic Clone Streptococcus pneumoniae Spain23F ST81. *Journal of Bacteriology*. **191**: 1480.

Darling AE, Mau B and Perna NT. 2010. progressiveMauve: Multiple Genome Alignment with Gene Gain, Loss and Rearrangement. *PLoS ONE*. **5**: e11147.

Dilthey A, Cox C, Iqbal Z, Nelson MR and McVean G. 2015. Improved genome inference in the MHC using a population reference graph. *Nature Genetics*. **47**: 682–688.

Dunne EM, Murad C, Sudigdoadi S, Fadlyana E, Tarigan R, Indriyani SAK, Pell CL, Watts E, Satzke C, Hinds J *et al.* 2018. Carriage of streptococcus pneumoniae, haemophilus influenzae, moraxella catarrhalis, and staphylococcus aureus in Indonesian children: A cross-sectional study. *PLoS ONE*. **13**:

Eizenga JM, Novak AM, Sibbesen JA, Heumos S, Ghaffaari A, Hickey G, Chang X, Seaman JD, Rounthwaite R, Ebler J *et al.* 2020. Pangenome Graphs. *Annual Review of Genomics and Human Genetics*. **21**:

Fan H, Ives AR, Surget-Groba Y and Cannon CH. 2015. An assembly and alignment-free method of phylogeny reconstruction from next-generation sequencing data. *BMC Genomics*. **16**:

Garrison E, Sirén J, Novak AM, Hickey G, Eizenga JM, Dawson ET, Jones W, Garg S, Markello C, Lin MF *et al.* 2018. Variation graph toolkit improves read mapping by representing genetic variation in the reference. *Nature Biotechnology*. **36**: 875–881.

Harris SR. 2018. SKA: Split Kmer Analysis Toolkit for Bacterial Genomic Epidemiology. *bioRxiv*. 453142. DOI: 10.1101/453142.

Hickey G, Heller D, Monlong J, Sibbesen JA, Sirén J, Eizenga J, Dawson ET, Garrison E, Novak AM and Paten B. 2020. Genotyping structural variants in pangenome graphs using the vg toolkit. *Genome Biology*. **21**: 35.

Holley G and Melsted P. 2020. Bifrost: highly parallel construction and indexing of colored and compacted de Bruijn graphs. *Genome Biology*. **21**: 249.

Kovács E, Sahin-Tóth J, Tóthpál A, van der Linden M, Tirczka T and Dobay O. 2020. Co-carriage of Staphylococcus aureus, Streptococcus pneumoniae, Haemophilus influenzae and Moraxella catarrhalis among three different age categories of children in Hungary. *PLoS ONE*. **15**:

Li H. 2018. Minimap2: pairwise alignment for nucleotide sequences. *Bioinformatics*. **34**: 3094–3100.

Mäklin T, Kallonen T, Alanko J, Samuelsen Ø, Hegstad K, Mäkinen V, Corander J, Heinz E and Honkela A. 2021. Bacterial genomic epidemiology with mixed samples. *Microbial Genomics*. **7**: 691.

Martin S, Heavens D, Lan Y, Horsfield S, Clark MD and Leggett RM. 2022. Nanopore adaptive sampling: a tool for enrichment of low abundance species in metagenomic samples. *Genome Biology*. **23**: 1–27.

Mavroidi A, Aanensen DM, Godoy D, Skovsted IC, Kaltoft MS, Reeves PR, Bentley SD and Spratt BG. 2007. Genetic Relatedness of the Streptococcus pneumoniae Capsular Biosynthetic Loci. *Journal of Bacteriology*. **189**: 7841.

Munro R, Wibowo S, Payne A and Loose M. 2024. Icarust, a real-time simulator for Oxford Nanopore adaptive sampling. *Bioinformatics*. **40**:

Ondov BD, Treangen TJ, Melsted P, Mallonee AB, Bergman NH, Koren S and Phillippy AM. 2016. Mash: Fast genome and metagenome distance estimation using MinHash. *Genome Biology*. **17**:

Payne A, Holmes N, Clarke T, Munro R, Debebe BJ and Loose M. 2021. Readfish enables targeted nanopore sequencing of gigabase-sized genomes. *Nature Biotechnology*. **39**: 442.

Sheppard CL, Manna S, Groves N, Litt DJ, Amin-Chowdhury Z, Bertran M, Ladhani S, Satzke C and Fry NK. 2022. PneumoKITy: A fast, flexible, specific, and sensitive tool for Streptococcus pneumoniae serotype screening and mixed serotype detection from genome sequence data. *Microbial Genomics*. **8**:

Sibbesen JA, Eizenga JM, Novak AM, Sirén J, Chang X, Garrison E and Paten B. 2023. Haplotype-aware pantranscriptome analyses using spliced pangenome graphs. *Nature Methods*. **20**: 239–247.

Viehweger A, Marquet M, Hölzer M, Dietze N, Pletz MW and Brandt C. 2023. Nanopore based enrichment of antimicrobial resistance genes - a case-based study. *GigaByte*. 1–15.
